# Design and In Vitro Evaluation of Novel GC373-like SARS-CoV-2 Main Protease Inhibitors

**DOI:** 10.3390/cimb48020142

**Published:** 2026-01-28

**Authors:** Aleksandra A. Kuznetsova, Aleksandr P. Makhin, Anatoliy A. Bulygin, Anastasia A. Andrianova, Vasily S. Miturich, Renata I. Zagitova, Vladimir I. Shmygarev, Anastasia A. Fadeeva, Oleg N. Yatskin, Olga A. Belozerova, Ivan V. Smirnov, Ilia V. Yampolsky, Zinaida M. Kaskova, Nikita A. Kuznetsov

**Affiliations:** 1 Institute of Chemical Biology and Fundamental Medicine, Siberian Branch of Russian Academy of Sciences, 630090 Novosibirsk, Russia; sandra-k@1bio.ru (A.A.K.); abulygin@1bio.ru (A.A.B.); 2 Shemyakin–Ovchinnikov Institute of Bioorganic Chemistry, Russian Academy of Sciences, 117997 Moscow, Russia; makhin.aleks@gmail.com (A.P.M.); nandrianova2000@gmail.com (A.A.A.); vsmiturich@gmail.com (V.S.M.); zagitova.renata97@gmail.com (R.I.Z.); vishmygarev@gmail.com (V.I.S.); nefi.nf99@gmail.com (A.A.F.); oleg.yatskin@mail.ru (O.N.Y.); o.belozyorova@gmail.com (O.A.B.); ivansmr@inbox.ru (I.V.S.); ivyamp@gmail.com (I.V.Y.); 3 Institute of Translational Medicine, Pirogov Russian National Research Medical University, 117997 Moscow, Russia; 4 Department of Natural Sciences, Novosibirsk State University, 630090 Novosibirsk, Russia

**Keywords:** SARS-CoV-2, COVID-19, peptidomimetic inhibitor, synthesis, antiviral drug design

## Abstract

Significant advances in coronavirus immunoprophylaxis have enabled the control of the SARS-CoV-2 pandemic. However, the continued emergence of SARS-CoV-2 variants with immune escape potential highlights the need for effective direct-acting antivirals targeting conserved viral enzymes. The SARS-CoV-2 main protease (M^pro^) remains one of the most promising antiviral drug targets due to its essential role in viral replication and the high conservation of its active site across coronavirus variants. Building upon the established GC373 scaffold, we designed, synthesized, and biochemically evaluated two novel GC373-like peptidomimetic inhibitors incorporated modified glutamine-mimic residues. These analogs were designed to enhance solubility and metabolic resilience while retaining key recognition features within the M^pro^ active site. Both compounds demonstrated micromolar inhibitory activity in enzymatic assays, supported by molecular docking and MM-PBSA analyses consistent with stable binding. The proposed inhibitors represent viable scaffolds for further optimization of electrophilic warheads and S1/S2 residue interactions. These findings contribute to the rational design of next-generation M^pro^ inhibitors and align with ongoing efforts to expand the chemical space of SARS-CoV-2 antiviral agents.

## 1. Introduction

The COVID-19 pandemic represents the third major coronavirus outbreak [1,2]. SARS-CoV-2 is a highly mutable virus that, since its emergence in 2019, has evolved and become more contagious, although less fatal [3,4]. New variants have necessitated ongoing development of effective vaccines [5]. An alternative approach to combating SARS-CoV-2 is the direct inhibition of its essential enzymes, particularly the main protease (M^pro^), which plays a critical role in viral replication. Currently, a few enzyme-targeted drugs have been approved: remdesivir [6], molnupiravir [7], nirmatrelvir [8,9], and ibuzatrelvir [10,11]. Among these, nirmatrelvir, a M^pro^ inhibitor, has proven to be the most successful. It is now available in tablet form, typically combined with ritonavir [12].

Despite its efficacy, as a prototype, nirmatrelvir (PF-07321332, PF-332) had certain limitations, including moderate solubility and bioavailability, susceptibility to metabolic degradation, and reduced effectiveness against certain M^pro^ polymorphs (e.g., L50F, E166V, L167F) [8,13,14,15,16]. Efforts to overcome these limitations have included the development of compounds with improved pharmacokinetic properties and reduced sensitivity to protease mutations [10,17,18,19,20]. Similarly, potent nanomolar M^pro^ inhibitors, such as PF-00835231 (PF-231) [21,22,23] and GC373 [24,25,26], as well as their analogs [27], remain limited by solubility and oral bioavailability.

Most peptidomimetic M^pro^ inhibitors currently under study incorporate a glutamic (pyrrolidin-2-one) residue, which is essential for protease recognition [28,29,30,31]. However, there remains a need for novel derivatives, featuring Gln mimic residues, that maintain high inhibitory potency while potentially improving pharmacokinetic and solubility properties [32,33].

In this study, we designed and synthesized new GC373-inspired M^pro^ inhibitors incorporating modifications predicted to influence solubility and without sacrificing binding properties. The compounds were evaluated using a combination of molecular modeling, including molecular dynamics simulations and in vitro enzymatic assays. This approach aims to provide a framework for the rational design of next-generation peptidomimetic SARS-CoV-2 M^pro^ inhibitors.

## 2. Materials and Methods

### 2.1. Computational Analysis

Initial structures of the complexes of SC2M^pro^ dimer with the inhibitors were obtained from the Protein Data Bank: 6XHM with PF-231 [21], 7SI9 with PF-332 [34], and 7CB7 with GC373 [35]. The dimeric form of M^pro^ from 6XHM entry corresponding to the Wuhan strain of SARS-CoV-2 was used as a basic structure for the modeling of new compounds. New compounds were docked to the protein by aligning with GC373. The simulations were carried out for the complexes without a covalent bond between the enzyme and an inhibitor. The parameters of all ligands were calculated with the help of the Acpype online service (www.bio2byte.be/acpype/ (accessed on 02 January 2026)).

The simulations were performed with GROMACS (version 2020.6)^39^ and the AMBER ff99SB-ILDN force field [36]. Preparation of the structures was performed using GROMACS built-in programs. The simulations were performed at 300 K with histidine residues protonated at the ε-positions. The protonation state was chosen according to the protein crystal structures, in particular 6XHM, where histidine residues form hydrogen bonds as they are protonated in ε-positions. The protonation states and formal charges of all inhibitors were assigned by analogy to GC373, assuming the neutral form. The Verlet cutoff scheme [37] was chosen with a cutoff of 1.2 nm for both van der Waals and electrostatic interactions, whereas H-bonds were constrained by the LINCS method [37]. Electrostatic interactions were computed in PME [38]. The solvated systems were minimized through steepest-descent minimization. After that, the systems were equilibrated in two stages with restraints on DNA and protein heavy atoms: for 200 ps in the NVT ensemble and for subsequent 200 ps in the NPT ensemble. Productive dynamics were implemented with a 2 fs time step in the NPT ensemble. All the simulations were carried out in duplicate. Final structures in two trajectories of each complex were the same within a fluctuation magnitude.

Binding free energies of complexes were calculated by combining MD data with the molecular mechanic/Poisson–Boltzmann surface area method. The “g_mmpbsa” script (version 3.0) for GROMACS data [39] was used to calculate the free energies of MM-PBSA. Using this script, electrostatic, VdW, polar solvation, and solvent accessible surface area (SASA) energies can be calculated, which combined give the total binding energy. For calculations, 500 consecutive frames from the last 100 ns of each trajectory were used. Those were the frames where each compound had the maximum number of contacts with the enzyme. Although the g_mmpbsa script provides relative binding energies, the method is appropriate for a comparative analysis and ranking of closely related ligands.

### 2.2. Synthesis of GC373-OxIm and GC373-Hyd

Full information of the synthetic procedures for **GC373-OxIm** and **GC373-Hyd** is available in the Supporting Information file.

#### 2.2.1. Synthesis of Benzyl (4-Methyl-1-oxo-1-((1-oxo-3-(2-oxoimidazolidin-1-yl)propan-2-yl)amino) pentan-2-yl)carbamate (GC373-OxIm)

To the solution of S-ethyl 2-(2-(((benzyloxy)carbonyl)amino)-4-methylpentanamido)-3-(2-oxoimid-azolidin-1-yl)propanoate (49 mg, 0.10 mmol, 1 eq.) in 1,4-dioxane (2 mL) under an argon atmosphere, triethylsilane (48 μL, 0.42 mmol, 4 eq.) and 5% Pd/C (11 mg, 5 mol%) were added. The mixture was stirred at r.t. for 30 min. The formation of aldehyde was monitored by LC-MS. Then Pd/C was removed by filtration through Celite. After the removal of Pd/C by filtration through Celite, the reaction mixture was evaporated. The resulting residue was purified by column chromatography (SiO_2_; CHCl_3_-MeOH gradient from 98:2 to 95:5) to yield **GC373-OxIm** as a colorless oil (8 mg; 19%). Due to the low stability of the resulting aldehyde **GC373-OxIm** at r.t., it was isolated and purified as quickly as possible and stored in the freezer at –80 °C.

#### 2.2.2. Analytical Data for GC373-OxIm

R*_f_* = 0.45 (CHCl_3_–MeOH, 9:1). ^1^H NMR (700 MHz, CDCl_3_): δ 9.16 (s, 1H), 7.39–7.29 (m, 5H), 5.24–4.98 (m, 2H), 4.53–3.16 (m, 8H), 1.83–1.45 (m, 3H), 1.05–0.79 (m, 6H). HRMS (ESI+) m/z: calc. for C_20_H_29_N_4_O_5_^+^ ([M+H]^+^) 405.2132, found: 405.2137.

#### 2.2.3. Synthesis of Benzyl (1-((1-(2,4-Dioxoimidazolidin-1-yl)-3-oxopropan-2-yl)amino)-4-methyl-1-oxopentan-2-yl)carbamate (GC373-Hyd)

To the mixture of THF (200 μL) and DMSO (50 μL, 0.70 mmol, 8 eq.) at −78 °C under an argon atmosphere, oxalyl chloride (50 μL, 0.35 mmol, 4 eq.) was added dropwise with stirring. The reaction mixture was stirred at −78 °C for 30 min, after which a solution of benzyl (1-((1-(2,4-dioxoimidazolidin-1-yl)-3-hydroxypropan-2-yl)amino)-4-methyl-1-oxopentan-2-yl)carbamate (37 mg, 0.09 mmol, 1 eq.) THF (1 mL) was added dropwise. The reaction mixture was stirred at −78 °C for 1 h, followed by the addition of triethylamine (98 μL, 0.70 mmol; 8 eq.). The reaction was then gradually warmed to −30 °C over 30 min, stirred at −30 °C for an additional 1 h, then gradually warmed up to 0 °C over 30 min, diluted with THF (2 mL), and quenched with saturated ammonium chloride solution (2 mL). The mixture was extracted with THF (3 × 5 mL). The organic layer was dried over anhydrous Na_2_SO_4_, and then the solvent was evaporated. The crude residue was purified by column chromatography (SiO_2_; CHCl_3_–MeOH gradient from 98:2 to 95:5) to yield product **GC373-Hyd** (5 mg, 13%) as a colorless oil. Due to the low stability of the resulting aldehyde **GC373-Hyd** at r.t., it was isolated and purified as quickly as possible and stored in the freezer at –80 °C.

#### 2.2.4. Analytical Data for GC373-Hyd

R*_f_* = 0.31 (CHCl_3_–MeOH = 9:1). ^1^H NMR (300 MHz, CDCl_3_): δ 9.58 (d, *J* = 10.4 Hz, 1H), 9.15–8.78 (m, 1H), 7.75 (dd, *J* = 22.0, 7.1 Hz, 1H), 7.38–7.23 (m, 5H), 5.50 (d, *J* = 8.8 Hz, 1H), 5.16–5.01 (m, 2H), 4.74–3.56 (m, 6H), 1.64 (s, 3H), 1.03–0.54 (m, 6H). ^13^C NMR (75 MHz, CDCl_3_) δ 173.9, 170.7, 157.8, 156.7, 156.6, 136.1, 128.7, 128.5, 128.4, 67.6, 58.3, 53.7, 51.4, 41.6, 41.2, 24.9, 23.0, 21.9. HRMS (ESI+) m/z: calc. for C_20_H_27_N_4_O_6_^+^ ([M+H]^+^) 419.1925, found: 419.1936.

### 2.3. Protease Expression and Purification

The full-length M^pro^ protein was purified as described [40]. M^pro^ was concentrated up to 4 mg/mL in 50 mM Tris pH 7.5, glycerol solution was added to make up 50% of the volume, and enzyme solution was stored at–80 °C.

### 2.4. The Peptide Substrate and Standard Inhibitors

The kinetic assays were implemented using the FRET substrate (FRET-S), i.e., Dabcyl-KTSAVLQ↓SGFRKM-E(Edans)-NH_2_ (Institute of Bioorganic Chemistry, Russian Academy of Sciences (RAS), Moscow, Russia), and standard covalent inhibitor PF-00835231 (Selleckchem, Houston, TX, USA). FRET-S contains a main-protease cleavage site indicated by the arrow and was utilized as the substrate in the FRET-based cleavage assay. Stock solution of PF-00835231 was prepared in DMSO (final concentration 5.0 mM). Stock solutions of GC373-OxIm and GC373-Hyd were prepared in acetonitrile (final concentration 10 mM).

### 2.5. Pre-Steady-State Kinetic Assay

For the preliminary screening of M^pro^ inhibitors, stopped-flow measurements with fluorescence detection were carried out. An SX.20 stopped-flow spectrometer (Applied Photophysics Ltd., Leatherhead, Surrey, UK) equipped with a 150 W Xe arc lamp and an optical cell with a 2 mm path length was used, and the dead time is 1.0 ms. The fluorescence of Edans was excited at λ_ex_ = 340 nm and monitored at λ_em_ > 435 nm as transmitted by filter GG-435 (Schott, Mainz, Germany). All the experiments were conducted in reaction buffer consisting of 20 mM TrisHCl pH 7.3, 100 mM NaCl, 1.0 mM EDTA, and 1.0 mM DTT. M^pro^ (2.5 µM) was incubated with inhibitor (2.5 µM PF-231, 25 µM GC373, boceprevir, telaprevir, GC373-OxIm or GC373-Hyd) or the same volume of DMSO/acetonitrile for 15 min at 4 °C in reaction buffer. The solutions containing the enzyme or enzyme/inhibitor and substrate were loaded into two separate syringes of the stopped-flow instrument and were incubated for an additional 5 min at 25 °C prior to mixing. The reported concentrations are those in the reaction chamber after the mixing. The trace shown is an average of three or more individual experiments.

### 2.6. Steady-State Kinetic Assay

In order to determine the inhibition constant *K*_I_, 150 nM M^pro^ was incubated with different concentrations of inhibitor (from 1 µM to 0.5 mM) for 15 min at 4 °C in reaction buffer. The dilutions of inhibitor were made in such way that the final adding volume was the same for each concentration of inhibitor. Then, an equal volume of FRET-S solution (15–35 μM) was added to initiate the reaction (final volume 32 μL). The fluorescence signal of the reaction was monitored for 30 min at λ_ex_/λ_em_ = 355/460 nm (Thermo Scientific Varioskan Lux fluorimeter, Thermo Fisher Scientific Inc., Waltham, MA, USA). The initial rate of FRET-S cleavage by M^pro^ in the presence and absence of inhibitors at several substrate concentrations was calculated by linear regression for the initial region of the kinetic progress curves. Kinetic constants (V_max_ and *K*_M_) were derived by fitting the data without inhibitor to the Michaelis–Menten equation, V = V_max_ × [S]/(*K*_M_ + [S]), *k*_cat_ = V_max_/[E]. The inhibition constant *K*_I_ was derived by fitting the kinetic data with inhibitor to the equation V = *k*_cat_ × [E_0_] × [S_0_]/([S_0_] + (*K*_M_ × (1 + [I_0_]/*K*_I_))).

## 3. Results and Discussion

### 3.1. Design of New Covalent Inhibitors

**Figure 1:**
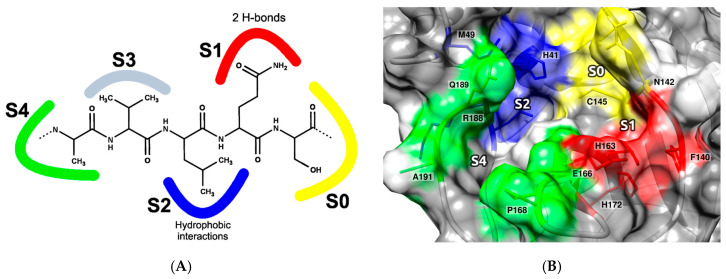
SARS-CoV-2 M^pro^ active site. (**A**) Schematic representation of natural peptide substrate in the active site sub-pockets S0-S4; (**B**) spatial structure of the M^pro^ active site pockets S0–S4.

**Figure 2:**
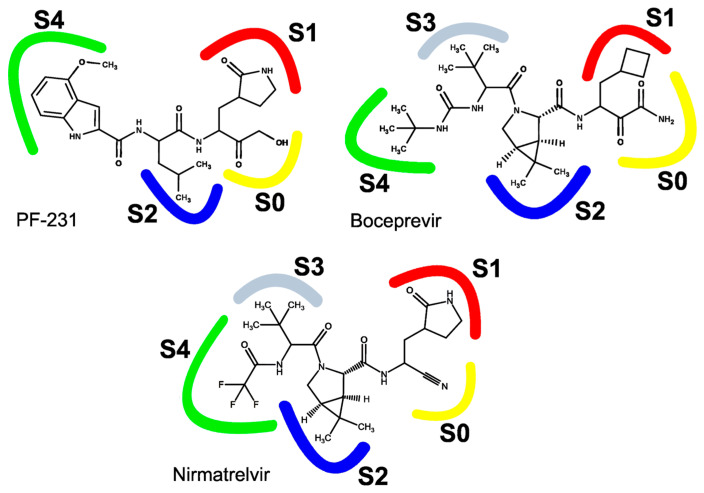
Position of side-chains of peptidomimetic inhibitors PF-231, boceprevir, and nirmatrelvir in the sub-pockets S0-S4 of the M^pro^ active site.

Each peptidomimetic M^pro^ inhibitor has four essential residues/substituents that correspond to the four active site pockets of the enzyme occupied by its natural substrate. In the case of M^pro^, the substrate has an AVLQS-sequence (1A), where A, L, Q, and S are the most important. S4, S2, S1, and S0 are the corresponding pockets of the protease (Figure 1B). In covalent inhibitors, S0-substituent, called the “warhead”, is responsible for covalent binding with Cys145 (Figure 1A). S1-substituent must resemble glutamine and have one H-bond acceptor and one H-bond donor to bind His163 and Glu166, respectively (Figure 1A,B). S2-substituent should be hydrophobic and resemble leucine. And, finally, S4-substituent may have various shapes as the S4-pocket is relatively flexible and can accommodate large aromatic structures, such as substituted indole in PF-231, long and bulky aliphatic structures such as that of boceprevir, or relatively small groups like trifluorinated carbon in nirmatrelvir (Figure 2).

Results of numerous studies of different substituents confirm that there is a strong correlation between hydrophobicity of the chemical group of the warhead and bioavailability of the whole compound. A bulky hydrophobic warhead, such as arylketone, gives better bioavailability but severely decreases solubility and binding ability [21,41,42], whereas very polar warheads, such as hydroxyketone or ketoamide, decrease bioavailability [8,23,43]. S1- and S2-substituents were not extensively varied as S1- and S2-pockets impose significant restrictions on corresponding substituents. It has been shown, however, that S1-substituent does not resemble glutamine and does not have an H-bond acceptor or H-bond donor group, and it is far inferior to glutamine or its analogs [44,45]. A non-hydrophobic, significantly bigger or significantly smaller S2-substituent also disrupts binding [9,27,40]. S4-substituent is the most variable part of all studied M^pro^ inhibitors. There is a moderate correlation between its hydrophobicity and bioavailability of the compound [9,40,46], but usually, the main purpose of varying S4-substituent is to achieve better binding. Currently, several very different moieties, from bulky and aromatic to small and aliphatic, have been found to ensure strong binding [9,21,40,46,47,48].

**Scheme 1:**
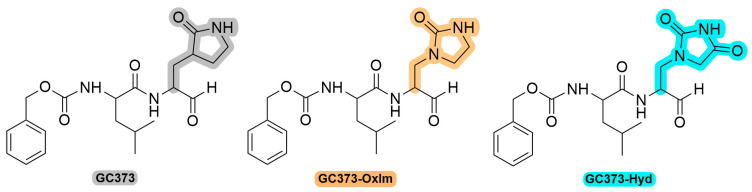
Structures of GC373 and two new derivatives of GC373 compounds, **GC373-OxIm** and **GC373-Hyd**, containing β-(S-2-oxoimidazolidine-3-yl)-alaninal or β-(S-2,4-imidazolidinedione-3-yl)-alaninal S1-substituents, respectively.

Based on the data above, we designed new GC373-like S1-substituents featuring a larger polar surface area, which should presumably be more water-soluble compared to the conventional β-(S-2-oxopyrrolidine-3-yl)-alaninal moiety, as supported by in silico SwissADME predictions (see SI [49,50,51,52,53,54,55,56,57,58], Table S1) [59], namely β-(S-2-oxoimidazolidine-3-yl)-alaninal (**GC373-OxIm**) and β-(S-2,4-imidazolidinedione-3-yl)-alaninal (**GC373-Hyd**) moieties (Scheme 1).

### 3.2. Computational Analysis of New M^pro^ Inhibitors

**Figure 3:**
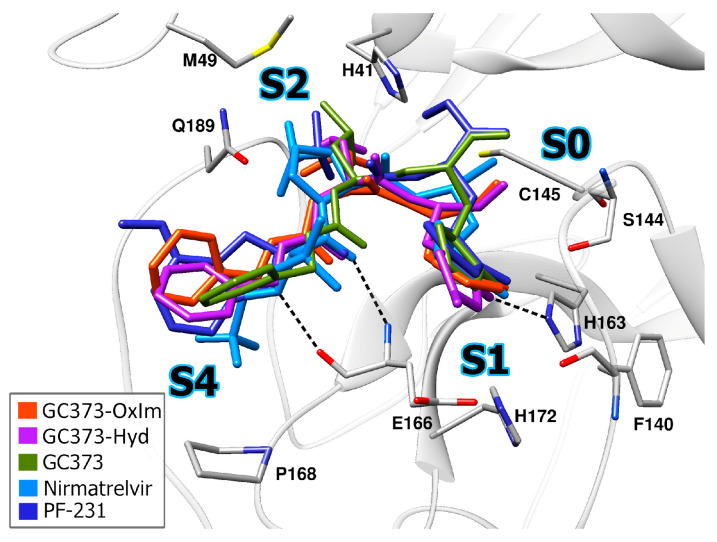
The complexes of M^pro^ with GC373, nirmatrelvir, PF-231, and two new compounds, **GC373-OxIm** and **GC373-Hyd**. Typical H-bonds are depicted by the dashed black lines. Heteroatoms in enzyme amino acid residues are depicted in blue (nitrogen), red (oxygen), and yellow (sulfur).

In order to predict the inhibitory capacity of the proposed compounds, the binding efficiency of inhibitors was determined using molecular dynamics computer modeling. Known protease inhibitors (PF-231, PF-332, and GC373 (an active form of GC376)) were selected for comparative analysis. The crystal structure of the complex of M^pro^ protease with the PF-231 inhibitor (PDB ID: 6XHM) was used as a starting structure for modeling the complex of M^pro^ protease with the chosen compounds (Figure 3). Molecules of all new compounds were located at the site of PF-231, which was possible due to their structural similarity. After obtaining the initial complexes with the new compound, MD simulations were performed for at least 300 ns. Simulation of complexes with known inhibitors also lasted at least 300 ns. Then, the last 5 ns was selected from the obtained molecular dynamics trajectories to calculate the binding energy of the inhibitor to the protease using the MM/PBSA (molecular mechanics/Poisson–Boltzmann surface area) method.

The molecular dynamics simulations and MM-PBSA calculations of PF-231, PF-332, and GC373 inhibitors as well as new compounds were performed (Table 1). We found that average binding energies of the two new compounds indicate a comparable or slightly improved binding efficacy relative to GC373: −137 and −139 kJ/mol for **GC373-OxIm** and **GC373-Hyd**, respectively, versus −130 kJ/mol for GC373.

**Figure 4:**
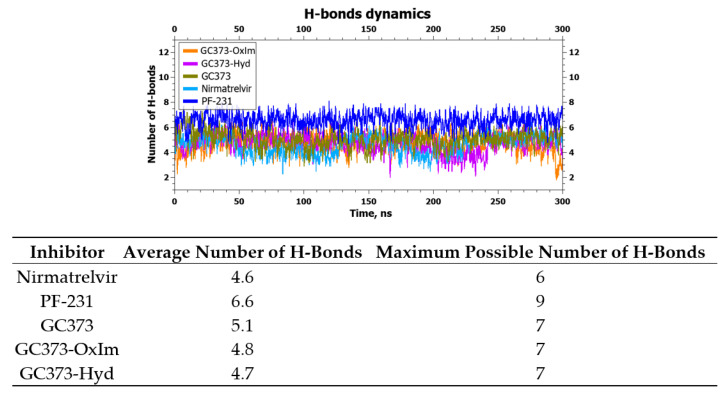
Number of H-bonds along MD simulation trajectory. The maximum and average numbers of H-bonds formed by inhibitors are shown in the left panel.

Analysis of structures in the course of MD trajectories of M^pro^ complexes with **GC373-OxIm** and **GC373-Hyd** (Figure 4) revealed that positionings of the new compounds are very similar to that of GC373. We noted only a slight difference in position of C^β^ of S1-substituent that, however, did not affect the H-bond configuration of this region of the inhibitor molecule. Indeed, the overall H-bond configuration of **GC373-OxIm** and **GC373-Hyd** was completely the same as in the case of GC373. However, the average number of H-bonds between the protein and new compounds slightly decreased to 4.8 and 4.7, respectively, if compared to GC373. Despite this decrease in the average number of H-bonds, this number is enough to stabilize the inhibitor molecule in the active site of protease, as supported by this parameter for nirmatrelvir (4.6 H-bonds over MD trajectory).

### 3.3. Synthesis of Inhibitor Compounds

**Scheme 2:**
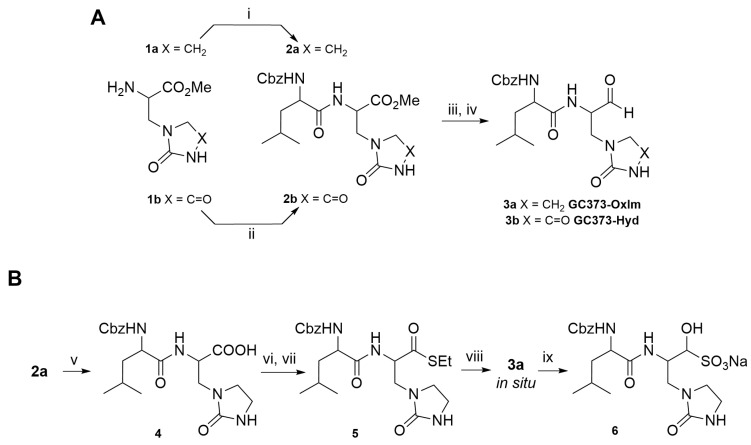
Synthesis of target aldehyde inhibitors **GC373-OxIm** and **GC373-Hyd**. (**A**) Synthesis of target aldehydes: (i) Cbz-*L*-Leu-OSu, Et_3_N, CH_3_CN, r.t.; (ii) Cbz-*L*-Leu-OSu, Et_3_N, DMF, r.t.; (iii) NaBH_4_, CH_3_OH, THF; (iv) oxalyl chloride, dimethyl sulfoxide, Et_3_N, THF, Ar, −78 °C; (**B**) Fukuyama synthesis of GC373-OxIm and in situ preparation of bisulfite adduct **6**: (v) NaOH aq., 1,4-dioxane, r.t.; (vi) ethyl chloroformate, Et_3_N, CH_2_Cl_2_, Ar, −10 °C; (vii) EtSH, DMAP; (viii) Et_3_SiH, Pd/C, 1,4-dioxane, Ar, r.t.; (ix) NaHSO_3_ aq., 1,4-dioxane, r.t.

Target compounds **GC373-OxIm** and **GC373-Hyd** were synthesized from previously obtained non-natural amino acid methyl esters methyl 2-amino-3-(2-oxoimidazolidin-1-yl)propanoate and 2-amino-3-(2,4-dioxoimidazolidin-1-yl)propanoate [60], respectively, in 4–8 steps. Both aldehydes **GC373-OxIm** and **GC373-Hyd** were highly unstable in the reaction mixture and during isolation, undergoing acid- and base-catalyzed condensation, which complicated their synthesis and purification (Scheme 2A). For inhibition activity tests, small amounts of synthetic aldehydes **GC373-OxIm** and **GC373-Hyd** were thawed immediately prior to measurements.

Conventional approaches for generating aldehyde **GC373-OxIm**, including direct DIBAL-H reduction of **2a** and various alcohol oxidation protocols (Swern [61], Parikh–Doering [62], Corey–Kim [63], Dess–Martin [64,65,66]), resulted in low yields (see Supporting Information for more detail). To overcome these limitations, a Fukuyama reduction [67,68,69] strategy was employed, with ethanethiol ester **5** serving as a key intermediate. Notably, reversed-phase chromatography was introduced as a critical purification step of **5**, preventing palladium catalyst deactivation in Fukuyama reduction. Furthermore, an unreported side reaction in Fukuyama reductions, wherein aldehyde **3a** undergoes acetone-mediated condensation, was identified and successfully mitigated by replacing acetone with 1,4-dioxane as a solvent. The bisulfite trapping method was implemented, allowing the in situ conversion of aldehyde **3a** (**GC373-OxIm**) to its bisulfite adduct **6** (Scheme 2B). Unlike aldehyde, the bisulfite derivative **6** was stable at room temperature, highly water-soluble, and readily purified via reversed-phase chromatography. Due to these advantages, in gram-scale procedures, aldehyde **3a** (**GC373-OxIm**) was exclusively used in its bisulfite-bound form **6**. These methodological advancements provide a scalable and robust synthetic route for highly reactive aldehydes, offering practical solutions for challenges associated with their isolation and handling. Although experimental solubility measurements could not be performed due to their low stability, in silico predicted LogS and LogP values suggest improved aqueous compatibility relative to GC373 (Table S1 in SI). Literature data suggest that under physiological conditions, the highly soluble bisulfite derivative **6** can revert to its active aldehyde form [70].

### 3.4. In Vitro Evaluation of Inhibition Activity

Our previous studies demonstrated that pre-steady-state analysis is an effective platform for screening small-molecule compounds to reveal their inhibitory potential [71]. A pre-steady-state kinetic assay, utilizing Förster resonance energy transfer (FRET), was conducted to characterize the catalytic cleavage of a peptide substrate (FRET-S) in the presence of inhibitors (PF-231, GC373, boceprevir, telaprevir, **GC373-OxIm 3a**, and **GC373-Hyd 3b**). The M^pro^ enzyme was pre-incubated with an inhibitor and kept on ice for 15 min to form an enzyme–inhibitor complex. Substrate hydrolysis was then initiated using stopped-flow fast mixing with this complex, and the FRET signal was monitored over 1000 s. Catalytic cleavage of the FRET-S peptide by M^pro^ increases the FRET signal due to the peptide bond hydrolysis and the release of the reaction product.

**Figure 5:**
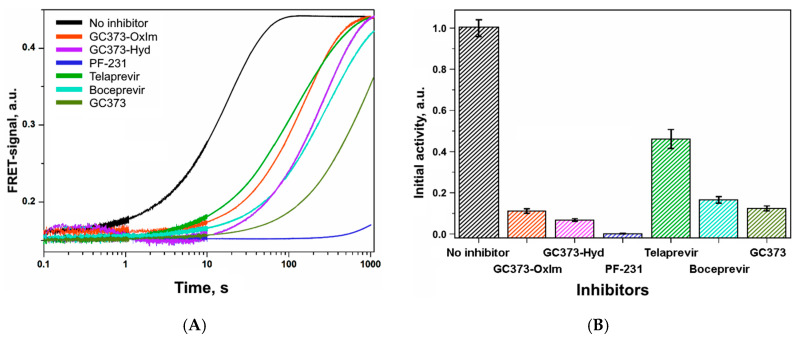
Comparative analysis of M^pro^ inhibition by inhibitors. (**A**) Inhibition profiles of various inhibitors; (**B**) residual enzymatic activity calculated from the initial slope of kinetic curves. The activity of free enzyme was normalized to 1.0. Concentrations used were 2.5 µM for FRET-S and enzyme, and 25 µM for inhibitors, except for PF-231 (2.5 µM).

The kinetics of FRET-S cleavage by free M^pro^ enzyme and its complexes with known protease inhibitors (PF-231, GC-373, boceprevir, and telaprevir) as well as novel compounds (**GC373-OxIm 3a** and **GC373-Hyd 3b**) were compared (Figure 5A). Residual enzymatic activity was quantified by calculating the initial slope of the FRET signal increase (Figure 5B). Comparative analysis of the data for known and new inhibitors revealed that both **GC373-OxIm** and **GC373-Hyd** significantly inhibit M^pro^ enzymatic activity, demonstrating their potency as effective M^pro^ inhibitors.

Steady-state analysis of the inhibition efficacy of the compounds enabled the determination of the inhibition constant *K*_I_. The inhibition constant was calculated from the dependence of the initial hydrolysis rate on inhibitor concentration (see Figures S58 and S59 in SM for kinetic experiments). The *K*_I_ values were determined as 0.92 ± 0.08 µM for **GC373-OxIm** and 2.0 ± 0.1 µM for **GC373-Hyd**. Micromolar *K*_I_ values reflect the first iteration of structural optimization. While these inhibitors are less potent than clinical M^pro^ blockers, they establish a viable scaffold for further tuning of electrophilic warheads and P1/P2 residues. Suboptimal synthetic yields indicate the need for alternative and more robust electrophilic motifs in future analogs, such as nitriles and ketoamides, which may also mitigate potential off-target effects associated with aldehyde warheads.

## 4. Conclusions

In this study, we designed and characterized two novel GC373-inspired peptidomimetic inhibitors of SARS-CoV-2 main protease (M^pro^), **GC373-OxIm** and **GC373-Hyd**. Guided by the structural analysis of the M^pro^ active site, we introduced glutamine-mimicking residues aimed at modulating physicochemical properties while preserving favorable binding interactions. Both compounds were synthesized and subjected to a pre-steady-state kinetic assay [71], which provided preliminary insight into their inhibition of M^pro^. While their potency remains moderate (*K*_I_ = 0.9 μM and 2.0 μM) compared with clinically approved inhibitors, these results serve primarily to validate the applied design strategy rather than to propose immediate therapeutic candidates. Potential improvements in solubility and other pharmacokinetic properties were supported by in silico calculations. Overall, this work provides a rational, methodology-driven framework for the development of next-generation M^pro^ inhibitors incorporating glutamine-like residues, contributing to ongoing research on SARS-CoV-2 and related coronaviruses.

## Figures and Tables

**Table 1:** MM-PBSA calculations of the complexes of dimeric M^pro^ with the known and new inhibitors (average ± SD, kJ/mol). The best from two duplicate trajectories of each complex result of calculated binding energies is provided.

Inhibitor	Electrostatic Energy	SASA Energy	Polar Solvation Energy	Van Der Waals Energy	Binding Energy
PF-00835231	−106 ± 10	−23 ± 1	245 ± 10	−257 ± 12	−141 ± 13
PF-07321332 (nirmatrelvir)	−86 ± 11	−21 ± 1	206 ± 11	−236 ± 13	−138 ± 13
GC373	−86 ± 11	−21 ± 1	206 ± 11	−229 ± 14	−130 ± 13
GC373-OxIm	−85 ± 10	−20 ± 1	208 ± 11	−240 ± 12	−137 ± 12
GC373-Hyd	−87 ± 11	−21 ± 1	205 ± 12	−236 ± 13	−139 ± 13

## Data Availability

The original contributions presented in this study are included in the article/Supplementary Material. Further inquiries can be directed to the corresponding authors.

## Supplementary Materials

The following supporting information can be downloaded at https://www.mdpi.com/article/10.3390/cimb48020142/s1.

## Abbreviations

The following abbreviations are used in this manuscript:

ADME	Absorption, Distribution, Metabolism, and Excretion
AMBER	Assisted Model Building with Energy Refinement
COVID	Coronavirus Disease
DIBAL-H	Diisobutylaluminium Hydride
DMSO	Dimethyl Sulfoxide
DNA	Deoxyribonucleic Acid
DTT	Dithiothreitol
EDTA	Ethylenediaminetetraacetic Acid
ESI	Electrospray Ionization
FRET	Förster Resonance Energy Transfer
GROMACS	GROningen MAchine for Chemical Simulations
HRMS	High-Resolution Mass Spectrometry
LC-MS	Liquid Chromatography–Mass Spectrometry
LINCS	Linear Constraint Solver
MD	Molecular Dynamics
MM/PBSA	Molecular Mechanics/Poisson–Boltzmann Surface Area
NMR	Nuclear Magnetic Resonance
NPT	Constant Number of Particles, Pressure, and Temperature Ensemble
NVT	Constant Number of Particles, Volume, and Temperature Ensemble
PDB	Protein Data Bank
PME	Particle Mesh Ewald
SARS-CoV-2	Severe Acute Respiratory Syndrome Coronavirus 2
SASA	Solvent Accessible Surface Area
THF	Tetrahydrofuran
VdW	Van der Waals
