# Supplementary material for: Design and In Vitro Evaluation of Novel GC373-like SARS-CoV-2 Main Protease Inhibitors
Source: Curr Issues Mol Biol. 2026 Jan 28;48(2):142. doi: 10.3390/cimb48020142 (PMC12939465; doi:10.3390/cimb48020142)
Supplement: Supplementary file 1 [file cimb-48-00142-s001.zip › cimb-4092054-supplementary.pdf]

Supporting Information for

Design and In Vitro Evaluation of Novel GC373-Like SARS-CoV-2 Main Protease Inhibitors

Aleksandra A. Kuznetsova, Aleksandr P. Makhin, Anatoly A. Bulygin, Anastasia A. Andrianova, Vasily S. Miturich, Renata I. Zagitova, Vladimir I. Shmygarev, Anastasia A. Fadeeva, Oleg N. Yatskin, Olga A. Belozerova, Ivan V. Smirnov, Ilia V. Yampolsky, Zinaida M. Kaskova, Nikita A. Kuznetsov

Contents

Prediction of ADME properties for target compounds 1
Materials and Methods 3
Synthesis of target compounds 4
NMR spectra of synthetic compounds 13
Kinetic Experiments (Figures) 70
References 71

Notes:

Substance numbering in *Supporting information* is **independent** from the main text.

NMR FID information for synthetic compounds is available free of charge at https://doi.org/10.5281/zenodo.15554221 (906.6 Mb).

Optical configuration of the chiral center at the *α*-carbon atom for compounds was not investigated.

Prediction of ADME properties for target compounds

The SwissADME online tool was used for the prediction of ADME properties [1]. This online tool allows the prediction of a number of important parameters, including solubility by the Ali [2], ESOL [3], and SILICOS-IT (www.silicos-it.be) methods, gastrointestinal absorption and brain penetration by the boiled egg method [4], and the possibility of cytochrome P450 inhibition. Moreover, several physicochemical properties are calculated, including flexibility, topological polar surface area [5], molecular weight, and others, which provides the possibility of estimating the drug likeness by the Lipinski [6], Ghose [7], Veber [8], Egan [9], and Muegge [10] criteria. Particular attention was given to solubility and GI absorption. The Ali method for solubility prediction was found to be the most valuable after various compounds with known parameters were tested, although other methods were also considered [11].

Table S1. Structures of known and newly designed inhibitors and their main predicted pharmacokinetic properties.

	Structure	SMILES	Binding energy, kJ/mol	Solubility, log(S)		GI absorption
				ESOL	Ali	
**PF-231**	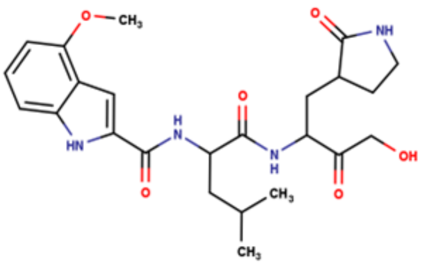	COC1=C2C=C(NC2=CC=C1)C(=O)NC(CC(C)C)C(=O)NC(CC1CCNC1=O)C(=O)CO	−141 ± 13	−3.1	−4.4	Low
**PF-332**	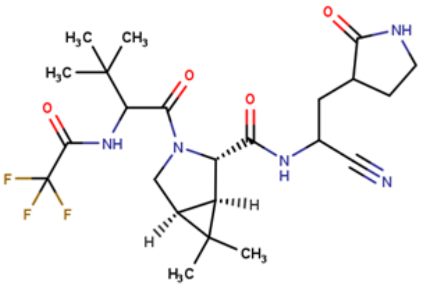	CC1([C@@H]2[C@H]1[C@H](N(C2)C(=O)[C@H](C(C)(C)C)NC(=O)C(F)(F)F)C(=O)N[C@@H](C[C@@H]3CCNC3=O)C#N)C	−138 ± 13	−3.6	−4.6	High

**GC373**	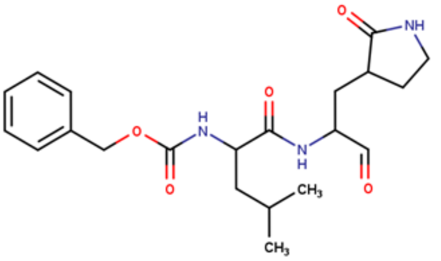	CC(C)CC(NC(=O)OCC1=CC=CC=C1)C(=O)NC(CC1CCNC1=O)C=O	−130 ± 13	−2.8	−3.9	High
**GC373-OxIm**	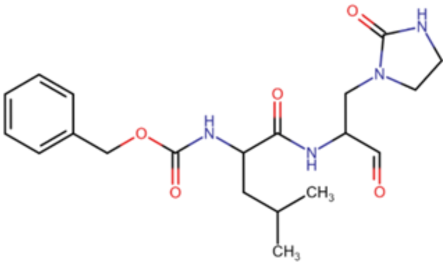	CC(C)CC(NC(=O)OCC1=CC=CC=C1)C(=O)NC(CN1CCNC1=O)C=O	−137 ± 12	−2.4	−3.2	High
**GC373-Hyd**	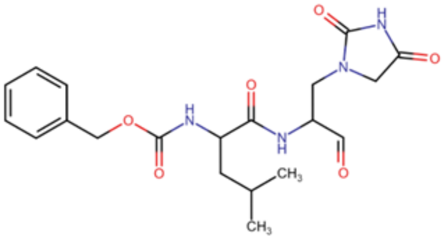	CC(C)CC(NC(=O)OCC1=CC=CC=C1)C(=O)NC(CN1CC(=O)NC1=O)C=O	−139 ± 13	−2.3	−3.4	High

Materials and Methods

Chemicals and synthesis. All chemicals were from Sigma-Aldrich (St. Louis, MO, USA) and Shanghai Macklin Biochemical Technology Co., Ltd. (Shanghai, China) in the purest grade available. Merck Kieselgel 60 was used for column chromatography. Thin-layer chromatography was performed on silica gel 60 F254 aluminum plates (Merck). Visualization was effected by UV light (254 nm) and staining with ninhydrin (ninhydrin 0.3 g, acetic acid 1 mL, ethanol 100 mL) and $KMnO_4$ (3 g $KMnO_4$, 20 g K_2CO_3, 5 mL sodium hydroxide solution 5% (m/v), 300 mL water).

NMR analysis. All NMR spectra of synthetic compounds were acquired in $CDCl_3$, acetone-d_6 (Eurisotop, France) and D_2O (Solvex-D, Russia) at 30°C on the Bruker Fourier 300 MHz, Bruker Fourier 300 MHz, and Avance I Bruker 700 MHz spectrometers. Residual solvent peaks were used as references (2.05 ppm and 29.84 ppm for ^1H and ^{13}C NMR, respectively, for acetone-d_6, 7.26 ppm and 77.16 ppm for ^1H and ^{13}C NMR, respectively, for $CDCl_3$, 4.79 ppm for ^1H NMR for D_2O).

LC-MS analysis of synthetic compounds was carried out on an ACQUITY UPLC H-Class System (Waters Corporation, USA) equipped with an ACQUITY UPLC BEH C18 Column (1.7 μm, 130Å, 50 mm X 2.1 mm), TUV, and SQD-ESI detectors. Samples were eluted with a H_2O-MeCN linear gradient (from 5 to 100% of MeCN for 5 min, 450 μL/min) with 0.1% formic acid as an eluent additive. UV data was collected at 220 nm, using an MS scan within the 50-750 Da range in positive and negative modes.

HRMS analysis of synthetic compounds was conducted on an Orbitrap Fusion Lumos mass spectrometer (ThermoFisher Scientific, USA). Samples were injected directly into the ESI ion source by a syringe in the solution of 0.1% FA/90% acetonitrile/10% H_2O. MS data were collected in DDA mode with the spectra recorded in positive and negative modes, with a 100-1000 mass range, at a 70K resolution for MS1, and a 17.5K resolution at 30 (N)CE and 1.4 m/z isolation window for MS2.

Synthesis of target compounds

Key starting non-natural amino acids (**1a,b**) were synthesized according to the described procedure [12] from protected *L*-asparagine as the initial building block (Scheme SI1). Methyl esters **3a, 3b** were obtained via coupling of corresponding non-natural amino acids **2a**, **2b** with *N*-benzyloxycarbonyl-*L*-leucine *N*-succinimidyl ester. Acids **4a,b** were obtained via basic hydrolysis of esters **3a, 3b**.

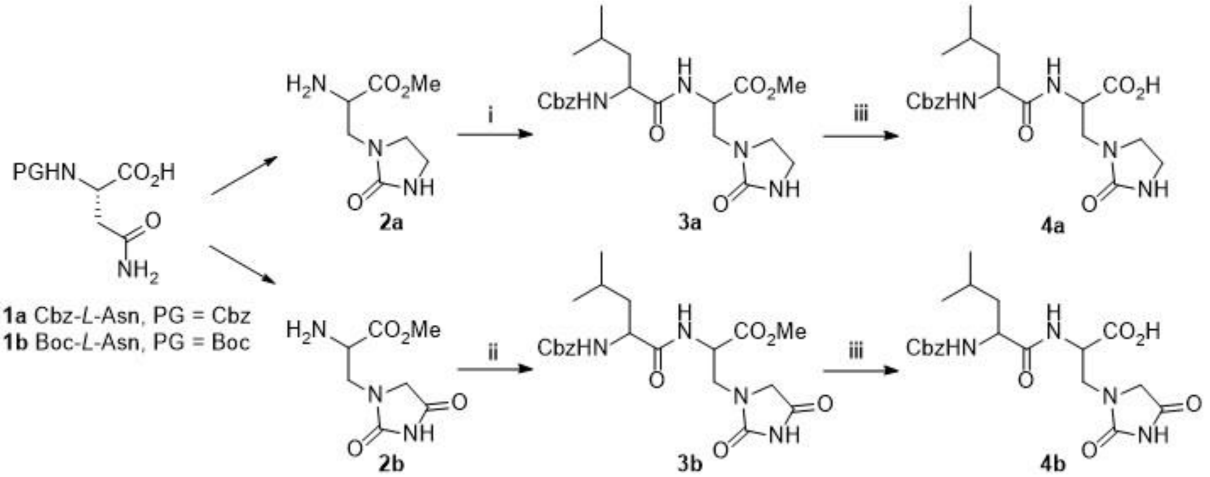

Scheme SI1. Synthesis of acids **4a,b.** (i) Cbz-*L*-Leu-OSu, Et_3N, CH_3CN, r.t.; (ii) Cbz-*L*-Leu-OSu, Et_3N, DMF, r.t.; (iii) NaOH aq., 1,4-dioxane, r.t.

During the screening stage, aldehyde **GC373-OxIm 6a** was obtained via direct DIBAL-H reduction of ester **3a**, while aldehyde **GC373-Hyd 6b** was prepared through Swern oxidation [13] of alcohol **5b** (Scheme SI2). However, both aldehydes **6a** and **6b** were highly unstable in the reaction mixture and during isolation, undergoing acid- and base-catalyzed condensation, which complicated their synthesis and purification.

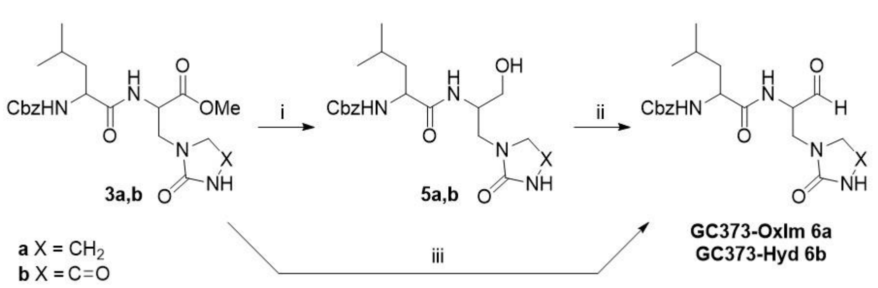

Scheme SI2. Strategies for synthesis of aldehydes **GC373-OxIm 6a** and **GC373-Hyd 6b**: (i) $NaBH_4$, CH_3OH, THF; (ii) oxalyl chloride, dimethyl sulfoxide, Et_3N, THF, Ar, −78 °C; (iii). DIBAL-H, CH_2Cl_2, −78 °C.

To improve the synthesis of **GC373-OxIm 6a**, as the compound demonstrated high inhibitor activity in vitro and was selected for further drug development, alcohol **5a** was first obtained in a 96% yield from ester **3a** via $NaBH_4$ reduction (Scheme SI2). Several oxidation methods, including Swern oxidation [13], Parikh–Doering oxidation [14], Corey–Kim oxidation [15], and Dess–Martin oxidation [16–18], failed to produce aldehyde **GC373-OxIm 6a** in satisfactory yields. Consequently, Fukuyama reduction [19–21] was explored as an alternative approach. Following Fukuyama's protocol [20], ethanethiol ester **7a** was synthesized in a one-pot reaction from acid **4a** (Scheme SI3). Ester **7a** had to be purified via reversed-phase chromatography on a C-18 sorbent before the next step. This procedure removed interfering impurities, prevented palladium catalyst poisoning, and allowed the recovery of unreacted acid **4a** for recycling.

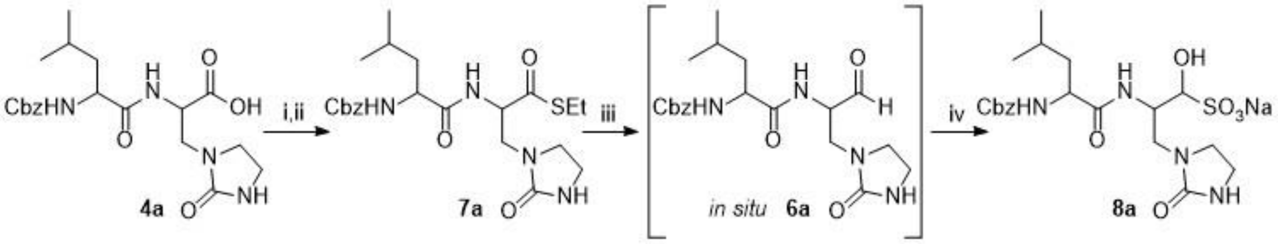

Scheme SI3. Fukuyama's synthesis of aldehyde **GC373-OxIm 6a**, and in situ preparation of **8a**: (i) ethyl chloroformate, Et_3N, CH_2Cl_2, Ar, -10°C; (ii) EtSH, DMAP; (ii) Et_3SiH, Pd/C, 1,4-dioxane, Ar, r.t.; (iv) $NaHSO_3$ aq., 1,4-dioxane, r.t.

Fukuyama reduction is typically performed in acetone as a solvent. However, when this method was applied to ethanethiol ester **7a**, the reaction in acetone resulted in an undesirable side reaction, producing a byproduct identified by LC-MS(ESI+) with *m/z* values of 445.40, 447.50. This byproduct was hypothesized to be a condensation product from aldehyde **GC373-OxIm 6a** and the solvent. By switching to 1,4-dioxane as a solvent, this issue was circumvented. However, purification of **GC373-OxIm 6a** was also challenging and was associated with a low yield (~ 20%), making the procedure unsuitable for gram-scale synthesis. To address the problem, the reaction mixture was treated with an aqueous sodium bisulfite ($NaHSO_3$) solution (Scheme SI3), converting aldehyde **8a** in situ into α-hydroxy sulfonate **9a** (56% yield over 2 stages). This modified approach proved suitable for gram-scale synthesis of compounds **3a-5a, 7a-8a**. Unlike the less stable aldehyde **GC373-OxIm 6a**, the

bisulfite derivative **8a** was stable at room temperature, highly soluble in water, and could be easily purified via reversed-phase chromatography. Due to these advantages, in gram-scale operations, aldehyde **GC373-OxIm 6a** was utilized solely in its bisulfite-bound form **8a**. Literature data suggest that under physiological conditions, the bisulfite derivative can revert to its active aldehyde form [22].

Methyl 2-(2-(((benzyloxy)carbonyl)amino)-4-methylpentanamido)-3-(2-oxoimid-azolidin-1-yl)propanoate (3a).

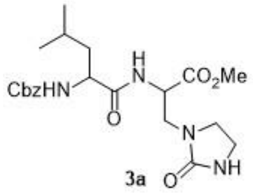

Methyl 2-amino-3-(2-oxoimidazolidin-1-yl)propionate **2a** (8.21 mmol, 1 eq.) was suspended in acetonitrile (130 mL). To this suspension, triethylamine (2.3 mL, 16.42 mmol, 2 eq.) and *N*-benzyloxycarbonyl-*L*-leucine *N*-succinimidyl ester (1.77 g, 9.44 mmol, 1.15 eq.) were added under stirring at room temperature, while the suspension was turning into an opalescent solution. The reaction progress was monitored by LC-MS. After stirring at r.t. for 1.5 hours, the solvent was evaporated to yield a colorless oil. The crude residue was purified by column chromatography (SiO_2; gradient from chloroform to chloroform–methanol 99:2) to yield product **3a** as a white glassy foam (2.92 g, 6.82 mmol, 83%).

R_f = 0.45 ($CHCl_3$-MeOH = 9:1);

^{1}H NMR (300 MHz, $CDCl_3$): δ 7.56 (d, *J* = 7.9 Hz, 1H), 7.40 – 7.27 (m, 5H), 5.57 (d, *J* = 8.6 Hz, 1H), 5.09 (s, 3H), 4.92 (s, 2H), 4.72 (ddd, *J* = 8.0, 7.9, 4.1 Hz, 2H), 4.36 – 4.22 (m, 1H), 3.72 (s, 3H), 3.69 – 3.26 (m, 6H), 1.79 – 1.41 (m, 3H), 0.99 – 0.80 (m, 6H).

^{13}C NMR (201 MHz, $CDCl_3$): δ 172.8, 170.7, 163.4, 156.2, 136.6, 128.6, 128.2, 128.1, 66.9, 53.7, 52.7, 51.7, 45.8, 45.3, 41.9, 38.5, 24.8, 23.1, 21.9. **HRMS (ESI+)** *m/z*: calc. for $C_{20}H_{27}N_4O_7^+$ ($[M+H]^+$) 435.2238, found 435.2246.

Methyl 2-(2-(((benzyloxy)carbonyl)amino)-4-methylpentanamido)-3-(2,4-dioxo-imidazolidin-1-yl)propanoate (3b).

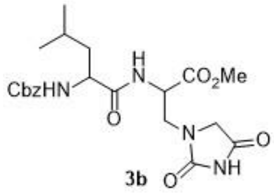

Methyl 2-amino-3-(2,4-dioxoimidazolidin-1-yl)propanoate **2b** (169 mg, 0.84 mmol, 1 eq.) was dissolved in DMF (1 mL). To this solution, triethylamine (234 µL, 1.68 mmol, 2 eq.) and *N*-benzyloxycarbonyl-*L*-leucine *N*-succinimidyl ester (350 mg, 0.97 mmol, 1.15 eq.) were added under stirring at r. t., while the solution was turning into a

suspension. The reaction progress was monitored by LC-MS. After stirring for 2 hours, the reaction mixture was diluted with water (300 µL). The resulting yellow solution was directly applied to the C-18 cartridge without any pre-separation steps. The product **2b** was isolated by RP-MPLC on the PuriFlash 5.250 system with a UV detector (Interchim, France). The purification was performed using cartridge Interchim C18, 15 µm, with the following eluent system: A – water, B – acetonitrile; gradient: 2 CV at 90% A, 10% B; 5 CV from 90% A, 10% B to 50% A, 50% B; 5 CV at 50% A, 50% B; flow rate: 5 mL/min; detection at λ = 210 nm. Fractions containing the pure product were lyophilized to yield **3b** as a white powder (180 mg, 48%).

R_f = 0.42 ($CHCl_3$-MeOH = 9:1);

^{1}H NMR (300 MHz, acetone-d_6): δ 9.71 (s, 1H), 7.79 (d, *J* = 8.2 Hz, 1H), 7.41 – 7.26 (m, 5H), 6.59 (dd, *J* = 8.5, 3.8 Hz, 1H), 5.15 – 4.99 (m, 2H), 4.83 – 4.69 (m, 1H), 4.33 – 4.15 (m, 1H), 4.15 – 3.92 (m, 2H), 3.84 – 3.64 (m, 5H), 1.82 – 1.67 (m, 1H), 1.67 – 1.51 (m, 2H), 0.96 – 0.86 (m, 6H).

^{13}C NMR (75 MHz, acetone-d_6): δ 173.5, 171.6, 171.2, 171.1, 158.0, 157.1, 138.1, 129.1, 128.7, 128.6, 66.9, 54.5, 52.7, 52.7, 52.5, 52.3, 51.9, 51.9, 51.8, 44.3, 44.3, 41.7, 41.6, 25.3, 23.3, 23.3, 21.9, 21.8.

HRMS (ESI+) *m/z*: calc. for $C_{21}H_{29}N_4O_7^+$ ($[M+H]^+$) 449,2031, found 449,2035.

2-(2-(((Benzyloxy)carbonyl)amino)-4-methylpentanamido)-3-(2,4-dioxoimidazolidin-1-yl)propanoic acid (4a).

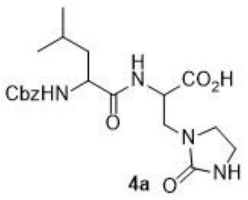

Method A: 1 M aqueous NaOH (36.7 mL, 36.7 mmol, 3 equiv.) was added to a solution of **3a** (5.32 g, 12.2 mmol, 1 equiv.) in 1,4-dioxane (37 mL). The homogeneous mixture was stirred at r.t. for 1.5 h. The reaction was monitored by TLC (eluent: $CHCl_3$ 79%, methanol 20%, acetic acid 1%). The pH of the solution was adjusted to 3–4 by adding 1 M aqueous HCl (37.0 mL). The reaction mixture was rotary evaporated, and the residue was dissolved in dichloromethane (20 mL). The precipitate was filtered through a Schott filter, and the supernatant was rotary evaporated to yield product **4a** as a white glassy foam (5.05 g, 98%, 95% purity by LC-MS(TUV)), which was then used in the next step without further purification.

Method B (Optional Purification). If necessary, the crude residue can be purified by

column chromatography (SiO_2; gradient: chloroform–methanol 98:2 to chloroform 79%, methanol 20%, acetic acid 1%).

R_f = 0.55 ($CHCl_3$ 79%, MeOH 20%, acetic acid 1%);

^{1}H NMR (300 MHz, $CDCl_3$): δ 7.66 (br.s, 1H), 7.39–7.25 (m, 5H), 5.70 (br.s, 1H), 5.08 (s, 2H), 4.67 – 4.59 (m, 1H), 4.31 – 4.25 (m, 1H), 3.70–3.26 (m, 6H), 1.70 – 1.45 (m, 1H), 0.90 (s, 3H).

^{13}C NMR (75 MHz, $CDCl_3$): δ 174.7, 172.5, 163.0, 155.6, 135.5, 127.6, 127.2, 126.9, 66.0, 57.4, 52.7, 44.3, 40.5, 37.6, 23.8, 22.1, 20.7.

HRMS (ESI+) *m/z*: calc. for $C_{20}H_{29}N_4O_6^+$ ($[M+H]^+$) 421.2082, found 421,2085.

2-(2-(((Benzyloxy)carbonyl)amino)-4-methylpentanamido)-3-(2,4-dioxoimidazolidin-1-yl)propanoic acid (4b).

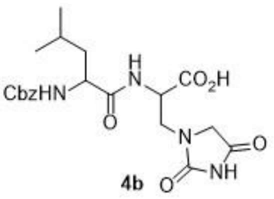

The substance **4b** was prepared from **3b** (45 mg, 0.10 mmol) according to the procedure described for **4a (Method A)**. The crude residue was purified by RP-HPLC using a Gilson system (France) equipped with pumps (models 305 and 306), a Rheodyne injector, and a UV-Vis detector (model 156). The purification was performed using a Luna 10 μm C18(2) column (250 × 4.6 mm) with the following eluent system: A – water, B – acetonitrile; gradient: 20 minutes from 90% A, 10% B to 100% B; flow rate: 5 mL/min; detection at λ = 210. Fractions containing the target product **4b** were rotary evaporated (15 mg, 35%).

R_f = 0.35 ($CHCl_3$ 79%, methanol 20%, acetic acid 1%).

^{1}H NMR (300 MHz, acetone-d_6) δ 9.68 (d, J = 9.1 Hz, 1H), 7.73 (d, J = 8.1 Hz, 1H), 7.49 – 7.23 (m, 5H), 6.59 (d, J = 8.1 Hz, 1H), 5.18 – 4.96 (m, 2H), 4.84 – 4.71 (m, 1H), 4.31 – 4.19 (m, 1H), 4.17 – 3.95 (m, 2H), 3.86 – 3.68 (m, 2H), 1.86 – 1.50 (m, 3H), 0.99 – 0.79 (m, 6H).

^{13}C NMR (75 MHz, acetone-d_6) δ 173.6, 171.6, 157.9, 157.2, 138.1, 129.2, 128.7, 128.6, 66.9, 54.5, 52.4, 52.3, 51.8, 51.8, 44.4, 41.7, 29.8, 25.4, 25.3, 23.4, 23.4, 21.9, 21.8.

HRMS (ESI+) *m/z:* calc. for $C_{20}H_{27}N_4O_7^+$ ($[M+H]^+$) 435.1874, found 435.1875.

Benzyl (1-((1-hydroxy-3-(2-oxoimidazolidin-1-yl)propan-2-yl)amino)-4-methyl-1-oxopentan-2-yl)carbamate (5a).

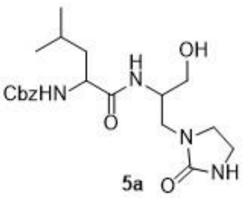

To a solution of **3a** (1.26 g, 2.90 mmol, 1 eq.) in tetrahydrofuran (110 mL) at 0°C, $NaBH_4$ (656 mg, 17.43 mmol, 6 eq.) was added slowly, followed by methanol (150 μL). The reaction mixture was stirred at 0°C for 30 min, then warmed to r.t. and stirred for an additional 1.5 h. The reaction was quenched with saturated NH_4Cl solution (30 mL) at 0°C, and the reaction mixture was extracted with ethyl acetate (3 × 100 mL). The organic layer was washed with brine (20 mL), dried over anhydrous Na_2SO_4, then the solvent was evaporated. The crude residue was purified by column chromatography (SiO_2; $CHCl_3$–MeOH gradient from 99:1 to 95:5) to yield **5b** as a glassy foam (1.13 g, 96%).

R_f = 0.57 ($CHCl_3$–MeOH = 9:1);

^{1}H NMR (300 MHz, $CDCl_3$): δ 7.39 – 7.27 (m, 5H), 7.21 (d, *J* = 8.5 Hz, 1H), 5.78 (d, *J* = 8.6 Hz, 1H), 5.18 – 5.00 (m, 2H), 4.30 – 4.16 (m, 1H), 4.16 – 3.96 (m, 1H), 3.77 – 3.01 (m, 8H), 1.75 – 1.40 (m, 3H), 0.92 (s, 3H), 0.90 (s, 3H).

^{13}C NMR (75 MHz, $CDCl_3$): δ 173.0, 163.9, 156.3, 136.5, 128.6, 128.2, 128.1, 67.0, 62.1, 53.9, 49.1, 45.9, 43.6, 42.0, 38.5, 24.8, 23.2, 21.9.

HRMS (ESI+) *m/z*: calc. for $C_{20}H_{31}N_4O_5^+$ ($[M+H]^+$) 407.2289, found 407.2292.

Benzyl (1-((1-(2,4-dioxoimidazolidin-1-yl)-3-hydroxypropan-2-yl)amino)-4-methyl-1-oxopentan-2-yl)carbamate (5b).

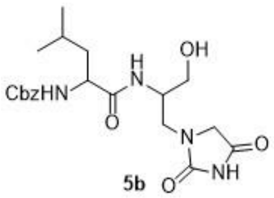

The substance **5b** was prepared from **3b** (131 mg, 0.29 mmol) according to the procedure described for **5a**. Yield **5b**: 104 mg, 84%.

R_f = 0.46 ($CHCl_3$–MeOH = 9:1);

^{1}H NMR (300 MHz, acetone-*d*$_6$) δ 9.74 (d, *J* = 6.9 Hz, 1H), 8.01 (d, *J* = 1.3 Hz, 1H), 7.42 – 7.24 (m, 5H), 5.91 (s, 1H), 5.15 – 4.99 (m, 2H), 4.35 – 3.26 (m, 8H), 1.85 – 1.47 (m, 3H), 0.98 – 0.81 (m, 6H);

^{13}C NMR (75 MHz, acetone-*d*$_6$) δ 173.7, 171.9, 158.5, 157.1, 138.1, 129.2, 128.7, 128.6, 79.2, 66.9, 62.5, 54.7, 52.5, 52.2, 50.5, 50.3, 43.9, 42.1, 41.8, 29.8, 25.4, 23.4, 21.9.

HRMS (ESI+) *m/z*: calc. for $C_{20}H_{29}N_4O_6^+$ ($[M+H]^+$) 421,2082. found: 421.2088.

Benzyl (4-methyl-1-oxo-1-((1-oxo-3-(2-oxoimidazolidin-1-yl)propan-2-yl)amino) pentan-2-yl)carbamate (GC373-OxIm 6a).

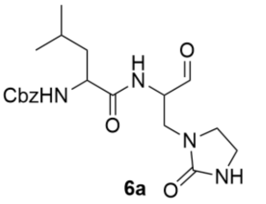

Compound **GC373-OxIm 6a** was prepared from **7a** (49 mg, 0.10 mmol) via Fukuyama reduction, following the general procedure described for **8a** (the synthesis of **8a** includes the in situ generation of **GC373-OxIm 6a** as an intermediate step). After the removal of Pd/C by filtration through Celite, the reaction mixture was evaporated. The resulting residue was purified by column chromatography (SiO_2; $CHCl_3$-MeOH gradient from 98:2 to 95:5) to yield **6a** as a colorless oil (8 mg; 19%). Due to the low stability of the resulting aldehyde **GC373-OxIm 6a** at r.t., it was isolated and purified as quickly as possible and stored in the freezer at –80°C.

R_f = 0.45 ($CHCl_3$–MeOH, 9:1);

^{1}H NMR (700 MHz, $CDCl_3$): δ 9.16 (s, 1H), 7.39 – 7.29 (m, 5H), 5.24 – 4.98 (m, 2H), 4.53 – 3.16 (m, 8H), 1.83 – 1.45 (m, 3H), 1.05 – 0.79 (m, 6H).

HRMS (ESI+) *m/z*: calc. for $C_{20}H_{29}N_4O_5^+$ ($[M+H]^+$) 405.2132, found: 405.2137.

Benzyl (1-((1-(2,4-dioxoimidazolidin-1-yl)-3-oxopropan-2-yl)amino)-4-methyl- 1-oxopentan-2-yl)carbamate (GC373-Hyd 6b).

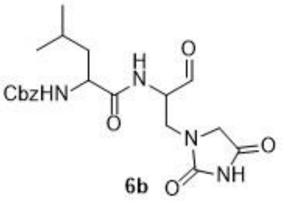

To the mixture of THF (200 µL) and DMSO (50 µL, 0.70 mmol, 8 eq.) at –78°C under an argon atmosphere, oxalyl chloride (50 µL, 0.35 mmol, 4 eq.) was added dropwise with stirring. The reaction mixture was stirred at –78°C for 30 min, after which a solution of **5b** (37 mg, 0.09 mmol, 1 eq.) THF (1 mL) was added dropwise. The reaction mixture was stirred at –78°C for 1 hour, followed by the addition of triethylamine (98 µL, 0.70 mmol; 8 eq.). The reaction was then gradually warmed to 0°C over 30 minutes, stirred at –30°C for an additional 1 hour, then gradually warmed up to 0°C over 30 minutes, diluted with THF (2 mL), and quenched with saturated ammonium chloride solution (2 mL). The mixture was extracted with THF (3 × 5 mL). The organic layer was dried over anhydrous Na_2SO_4, and then the solvent was evaporated. The crude residue was purified by column chromatography (SiO_2; $CHCl_3$–MeOH gradient from 98:2 to 95:5) to yield product **GC373-Hyd 6b** (5 mg, 13%) as a colorless oil. Due to the low stability of the resulting aldehyde **GC373-Hyd 6b** at r.t., it was isolated and purified as quickly as possible and stored in the freezer at –80°C.

R_f = 0.31 ($CHCl_3$–MeOH = 9:1);

^{1}H NMR (300 MHz, $CDCl_3$): δ 9.58 (d, *J* = 10.4 Hz, 1H), 9.15 – 8.78 (m, 1H), 7.75 (dd, *J* = 22.0, 7.1 Hz, 1H), 7.38 – 7.23 (m, 5H), 5.50 (d, *J* = 8.8 Hz, 1H), 5.16 – 5.01 (m, 2H), 4.74 – 3.56 (m, 6H), 1.64 (s, 3H), 1.03 – 0.54 (m, 6H).

^{13}C NMR (75 MHz, $CDCl_3$) δ 173.9, 170.7, 157.8, 156.7, 156.6, 136.1, 128.7, 128.5, 128.4, 67.6, 58.3, 53.7, 51.4, 41.6, 41.2, 24.9, 23.0, 21.9.

HRMS (ESI+) *m/z*: calc. for $C_{20}H_{27}N_4O_6^+$ ($[M+H]^+$) 419.1925, found: 419.1936.

S-Ethyl 2-(2-(((benzyloxy)carbonyl)amino)-4-methylpentanamido)-3-(2-oxoimid-azolidin-1-yl)propanoate (7a).

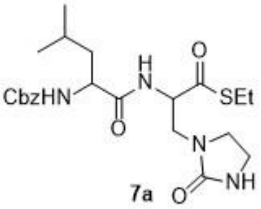

7a

To the solution of compound **4a** (5.15 g, 12.25 mmol, 1 eq.), prepared in situ by **Method A**, dichloromethane (144 mL) triethylamine (4.27 mL, 30.62 mmol, 2.5 eq.) and ethyl chloroformate (1.75 mL, 18.37 mmol, 1.5 eq.) were added at −10°C under stirring in an argon atmosphere. The reaction mixture was stirred for 20 minutes at −10°C, after which ethanethiol (5.60 mL, 73.49 mmol, 6.0 eq.), triethylamine (1.71 mL, 12.25 mmol, 1 eq.), and 4-dimethylaminopyridine (300 mg, 2.45 mmol, 0.2 eq.) were added. The reaction was monitored by LC-MS. The reaction mixture was stirred for an additional 20 min at −10°C, then warmed to r. t., and then 1 M HCl (70 mL) and saturated NaCl solution (70 mL) were added. The resulting mixture was extracted with dichloromethane (3 × 150 mL). The combined organic phases were rotary evaporated to yield a white glassy foam. The crude residue was purified by RP-MPLC on the PuriFlash 5.250 system with a UV detector (Interchim, France). The purification was performed using cartridge Interchim C18, 50 µm, with the following eluent system: A – water, B – acetonitrile; gradient: 5 CV from 70% A, 30% B to 30% A, 70% B; 5 CV at 30% A, 70% B; flow rate: 25 mL/min; detection at λ = 265 nm) to yield product **7a** (2.78 g, 49%), and recycled unreacted **4a** (258 mg**,** 5%).

R_f = 0.57 ($CHCl_3$–MeOH = 9:1);

^{1}H NMR (800 MHz, $CDCl_3$): δ 7.84 (d, *J* = 7.5 Hz, 1H), 7.46 – 7.27 (m, 5H), 5.27 (d, *J* = 7.9 Hz, 1H), 5.13 (s, 2H), 4.74 – 4.69 (m, 1H), 4.45 (s, 1H), 4.31 – 4.26 (m, 1H), 3.67 (dd, *J* = 15.1, 8.2 Hz, 1H), 3.53 – 3.30 (m, 3H), 2.90 – 2.79 (m, 2H), 1.82 – 1.67 (m, 2H), 1.58 – 1.51 (m, 1H), 1.22 (t, J = 7.4 Hz, 3H), 0.95 (s, 3H), 0.94 (s, 3H).

^{13}C NMR (201 MHz, CDCl$_3$): δ 200.1, 173.3, 163.7, 156.2, 136.6, 128.6, 128.3, 128.2, 77.3, 67.2, 59.3, 54.1, 46.4, 45.6, 41.6, 38.7, 24.9, 23.5, 23.1, 21.9, 14.5.

HRMS (ESI+) *m/z*: calc. for $C_{22}H_{33}N_4O_5S^+$ ([M+H]$^+$) 465.2166, found: 465.2170.

Sodium 2-(2-(((benzyloxy)carbonyl)amino)-4-methylpentanamido)-1-hydroxy-3-(2-oxoimidazolidin-1-yl)propan-1-sulfonate (8a).

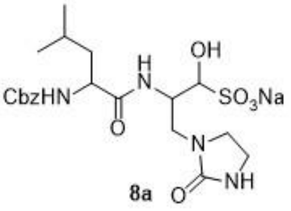

8a

To the solution of **7a** (1.32 g, 2.84 mmol, 1 eq.) in 1,4-dioxane (55 mL) under an argon atmosphere, triethylsilane (1.81 mL, 1.81 mmol, 4 eq.) and 5% Pd/C (301 mg, 5 mol%) were added. The mixture was stirred at r.t. for 30 min. The formation of aldehyde **6a** was monitored by LC-MS. Then Pd/C was removed by filtration through Celite. The Celite was washed with 1,4-dioxane (3 × 5 mL). The filtrate was poured with 1 M aqueous $NaHSO_3$ (34.12 mL, 34.12 mmol, 12 eq.) and water (9 mL). The resulting homogeneous solution was stirred for 15 minutes at room temperature. The formation of **8a** was monitored by LC-MS. The reaction mixture was concentrated on a rotary evaporator at 40°C. The product was isolated from the resulting solution by RP-MPLC using a PuriFlash 5.250 system with a UV detector. The purification was performed using cartridge Interchim C18, 15 µm, with the following eluent system: A – water, B – acetonitrile; gradient: 1 CV at 90% A, 10% B; 3 CV from 90% A, 10% B to 50% A, 50% B; 1 CV at 50% A, 50% B; 3 CV from 50% A, 50% B to 30% A, 70% B; flow rate: 15 mL/min; detection at λ = 210 nm). Fractions containing the pure product were lyophilized to yield **8a** as a white powder (773 mg, 56%).

^{1}H NMR (800 MHz, D$_2$O): δ 7.51–7.42 (m, 5H), 5.23–5.12 (m, 2H), 4.75–4.37 (m, 2H), 4.13–4.15 (m, 1H), 3.77–3.16 (m, 6H), 1.77–1.50 (m, 3H), 0.98–0.94 (m, 3H), 0.94–0.90 (m, 3H).

^{13}C NMR (201 MHz, D$_2$O): δ 175.2, 164.5, 157.6, 136.5, 128.9, 128.4, 127.7, 127.6, 83.5, 83.2, 81.5, 81.5, 67.5, 67.5, 67.15, 67.1, 54.3, 54.2, 54.2, 53.9, 48.9, 48.7, 48.2, 48.1, 45.3, 44.6, 44.5, 42.8, 42.2, 40.3, 40.1, 38.0, 24.4, 24.4, 22.4, 22.3, 22.3, 22.2, 20.9, 20.8, 20.7, 20.6.

HRMS (ESI+) *m/z*: calc. for $C_{20}H_{31}N_4O_8S^+$ ([M+H]$^+$) 487.1857, found: 487.1862.

NMR spectra of synthetic compounds

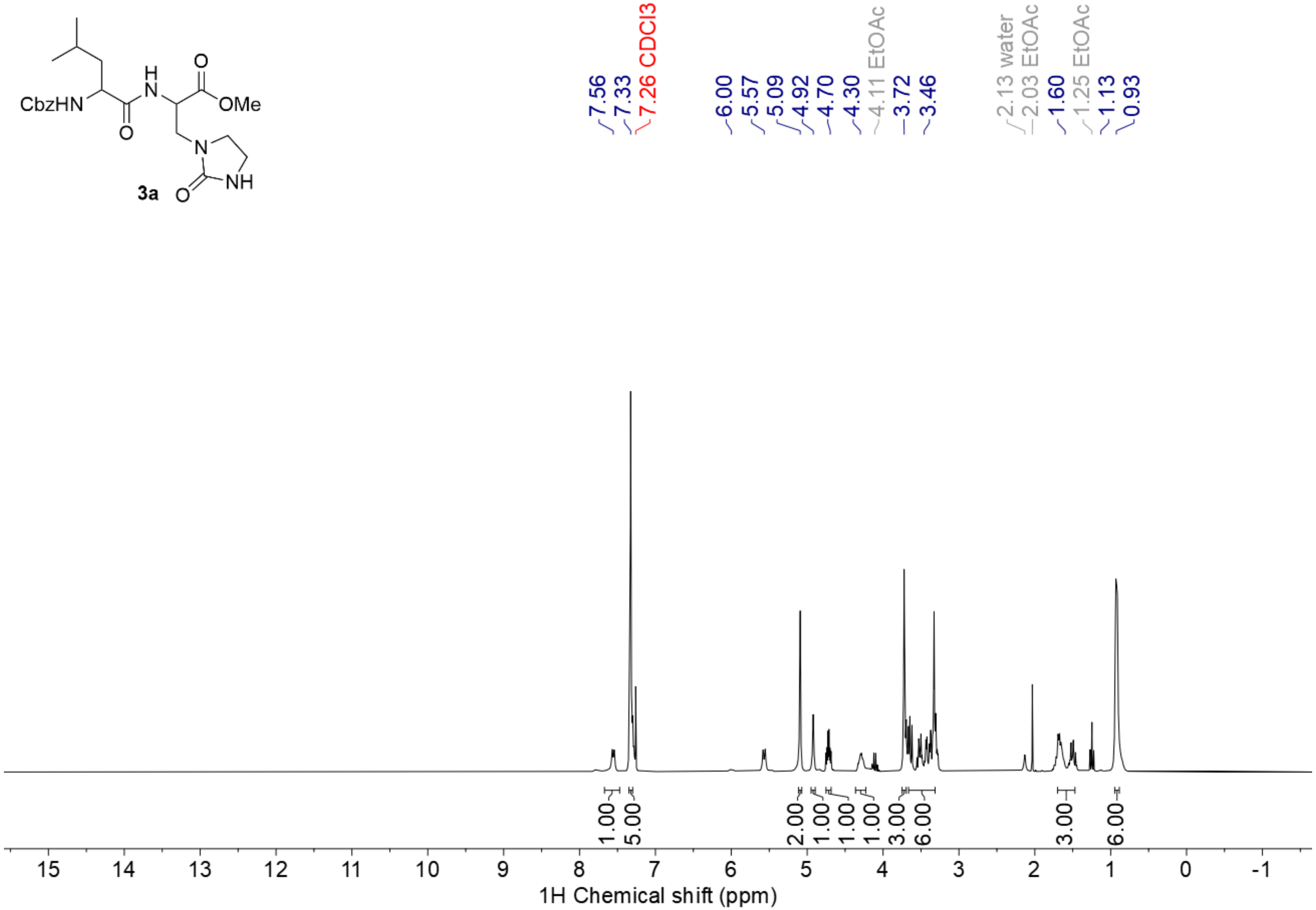

Figure SI1. ^{1}H NMR (300 MHz, $CDCl_3$) of compound **3a**.

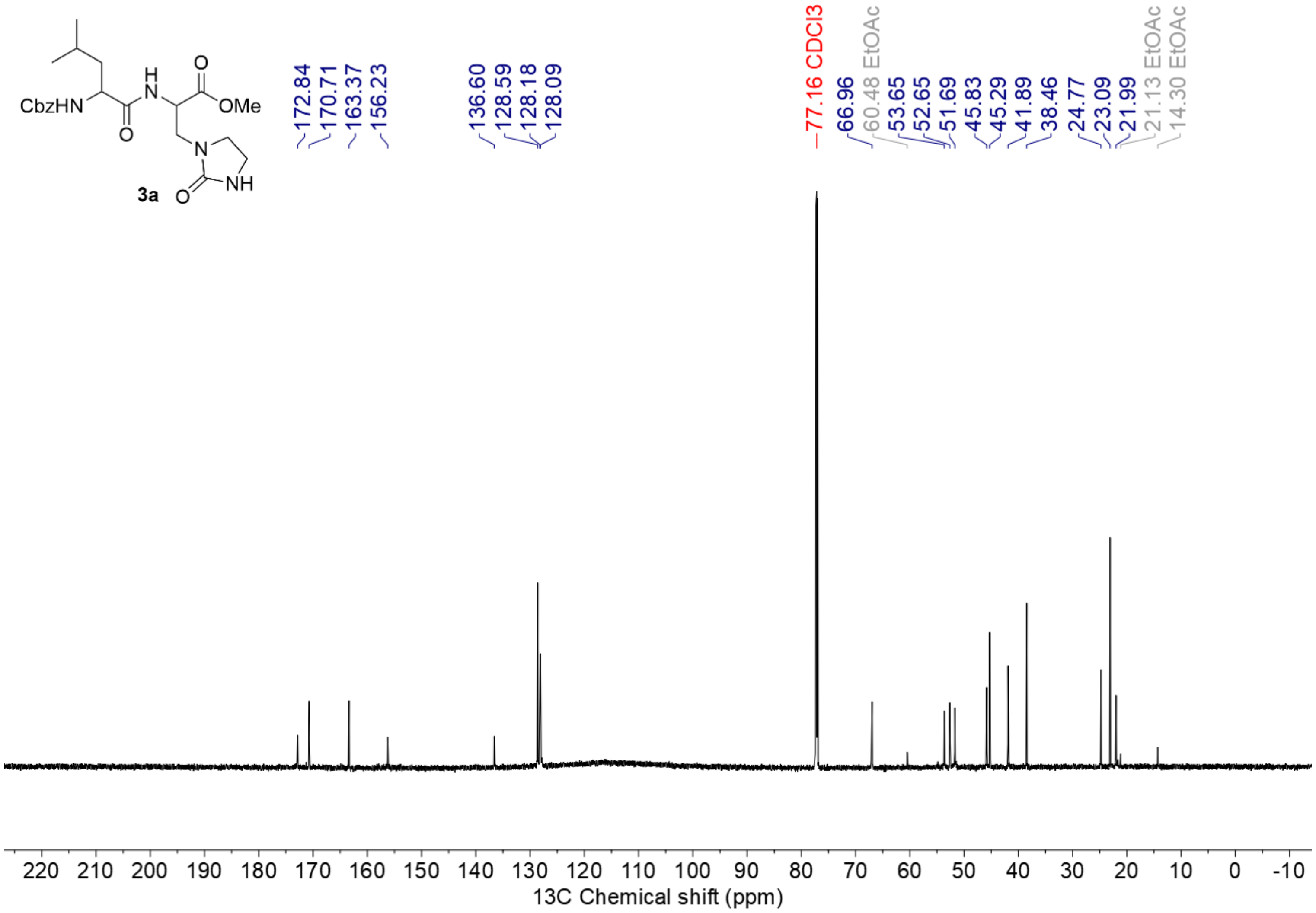

Figure SI2. ^{13}C NMR (201 MHz, $CDCl_3$) of compound **3a**.

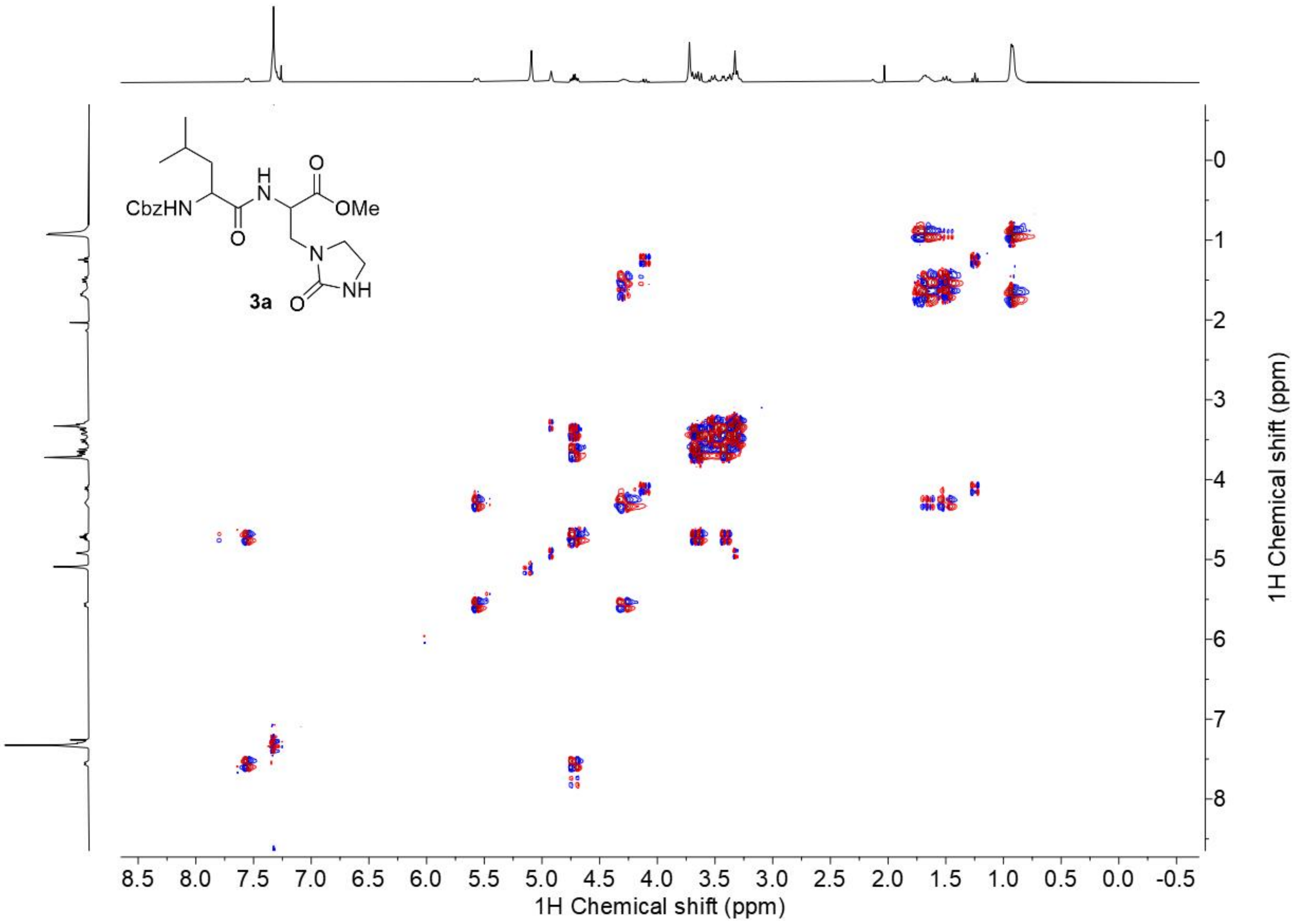

Figure SI3. ^{1}H-^{1}H COSY NMR (^{1}H 300 MHz, $CDCl_3$) of compound **3a**.

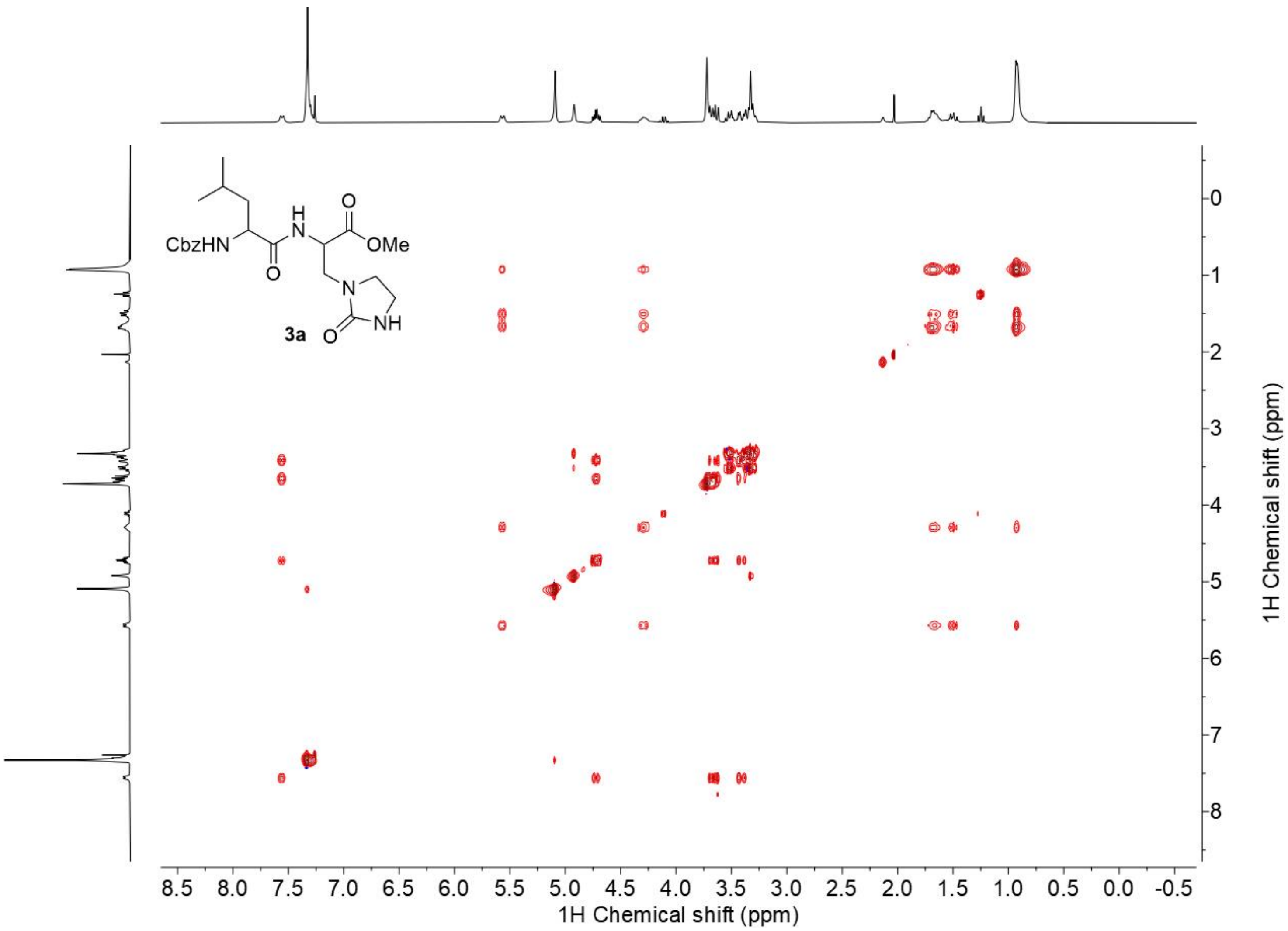

Figure SI4. ^{1}H-^{1}H TOCSY NMR (^{1}H 300 MHz, $CDCl_3$) of compound **3a**.

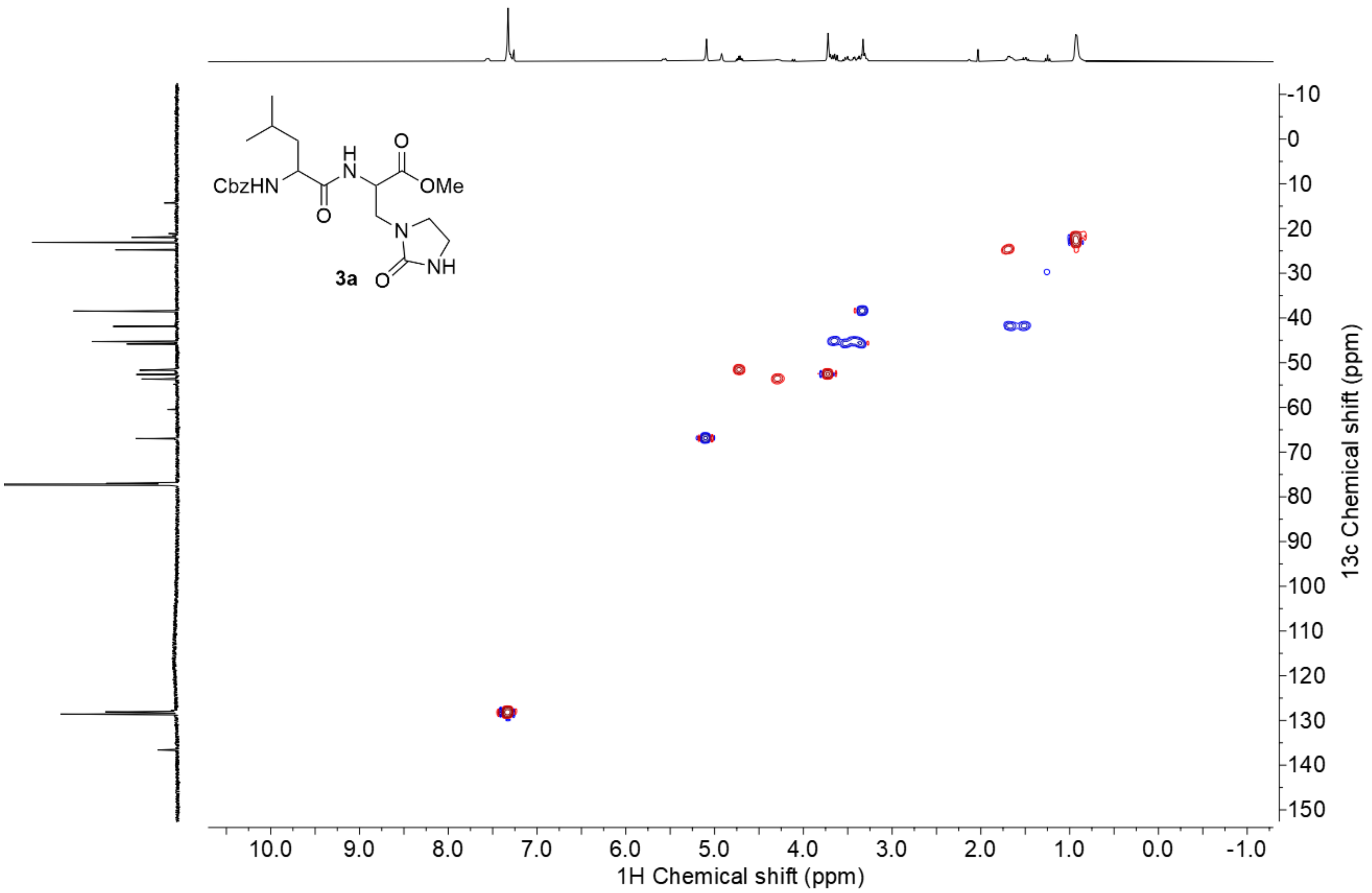

Figure SI5. ^{1}H-^{13}C HSQC-ED NMR (^{1}H 300 MHz, $CDCl_3$) of compound **3a**.

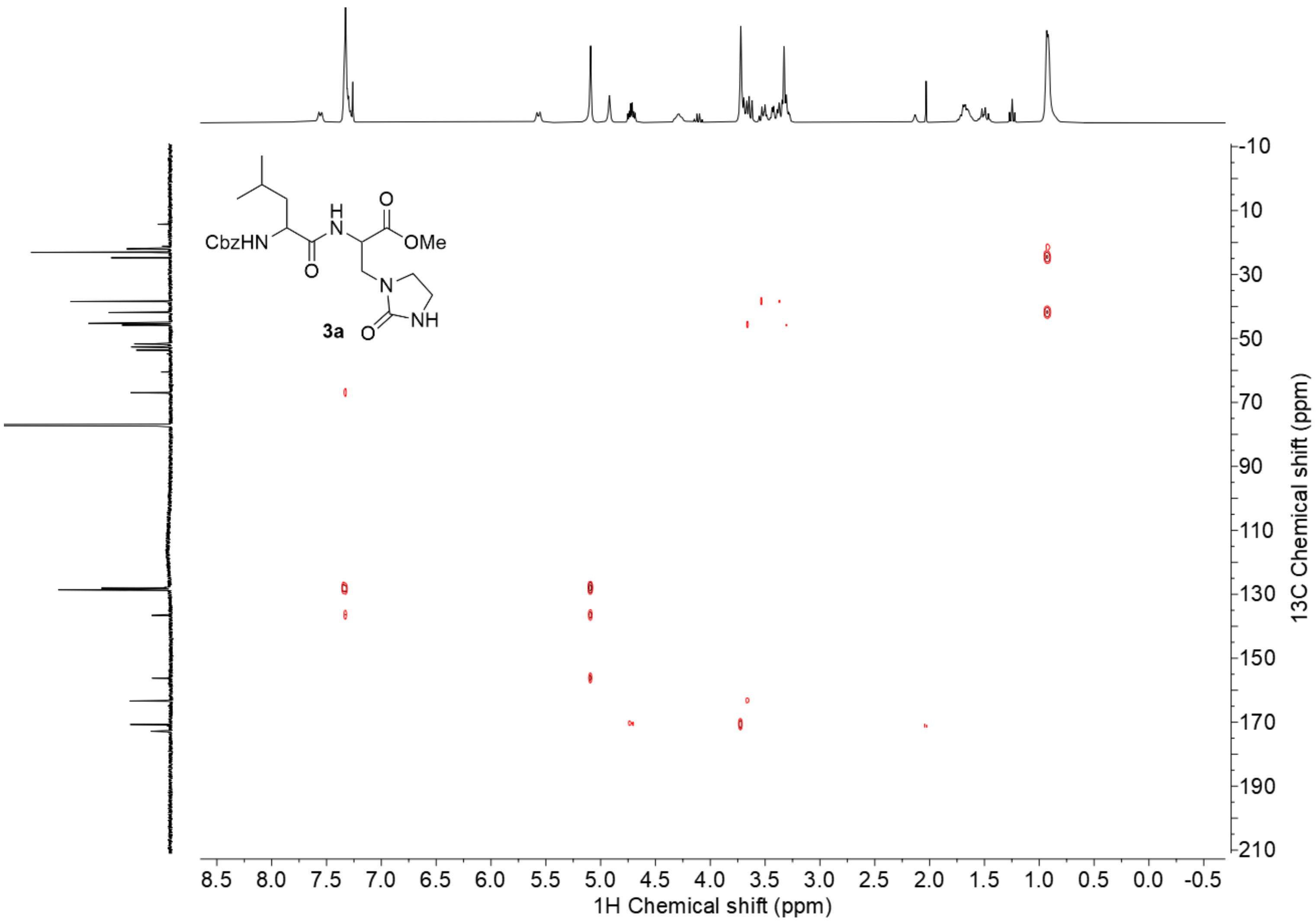

Figure SI6. ^{1}H-^{13}C HMBC NMR (^{1}H 300 MHz, $CDCl_3$) of compound **3a**.

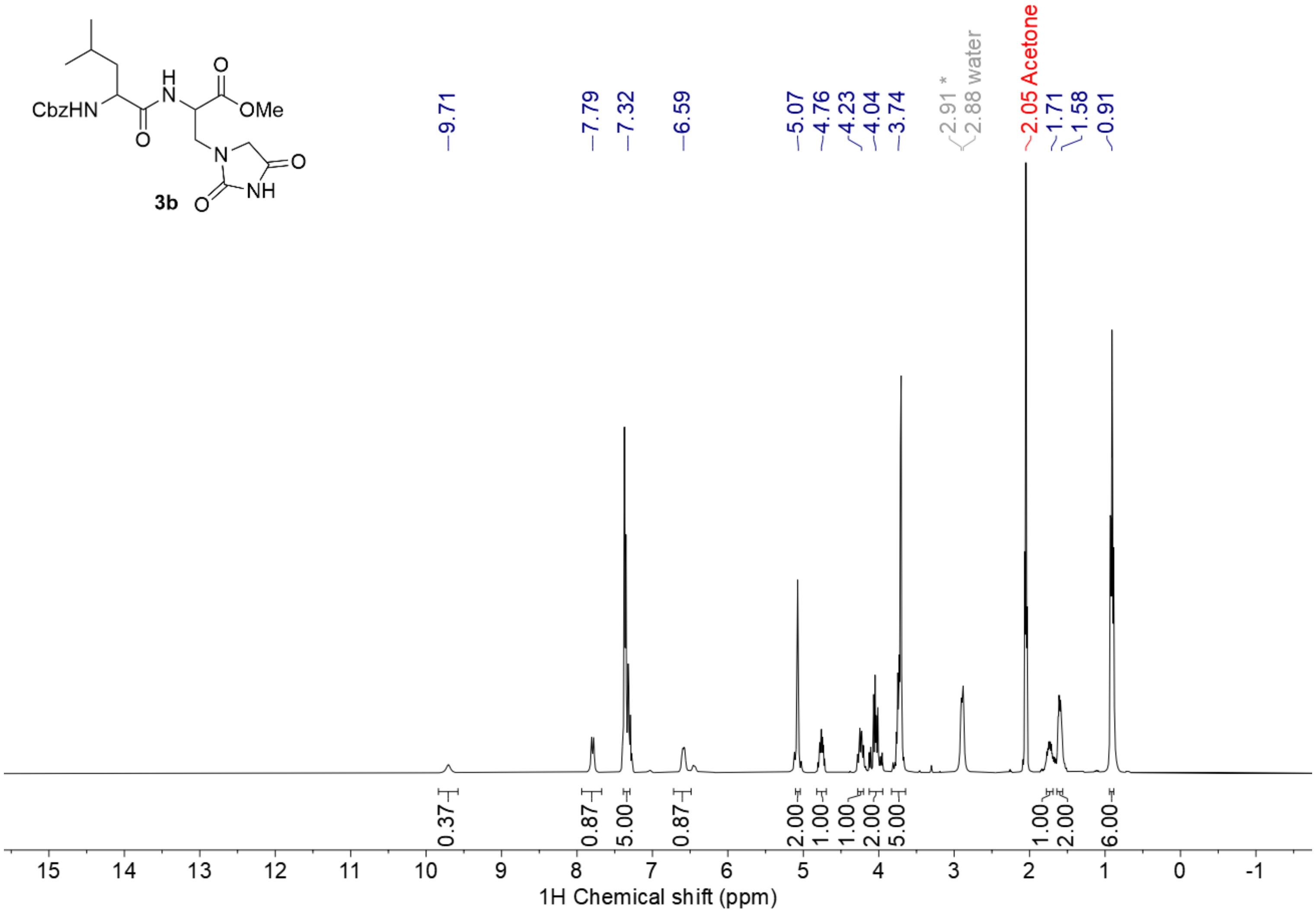

Figure SI7. ^{1}H NMR (300 MHz, acetone-d_6) of compound **3b**; * – impurity.

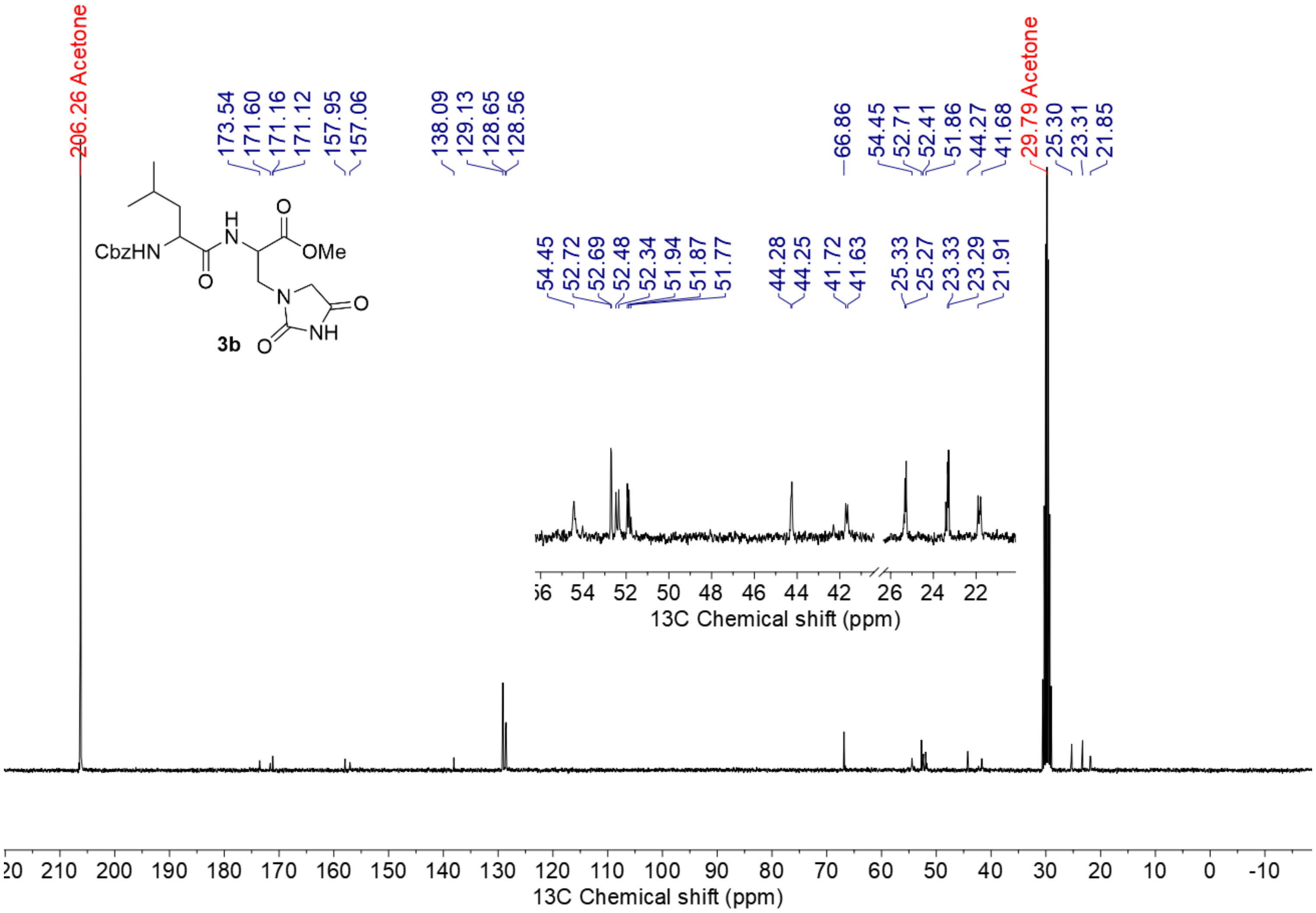

Figure SI8. ^{13}C NMR (75 MHz, acetone-d_6) of compound **3b**.

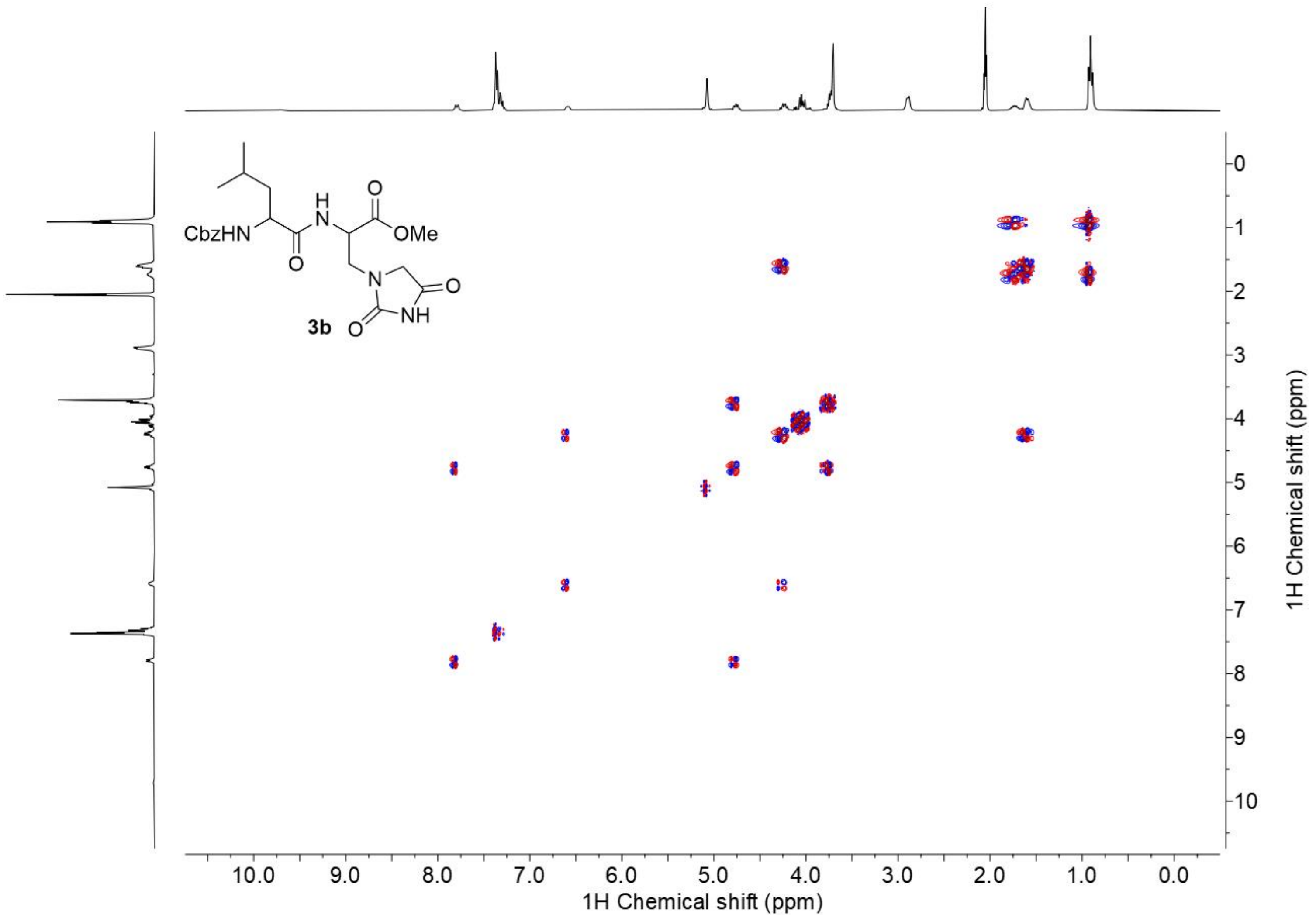

Figure SI9. ^{1}H-^{1}H COSY NMR (^{1}H 300 MHz, acetone-d_6) of compound **3b**.

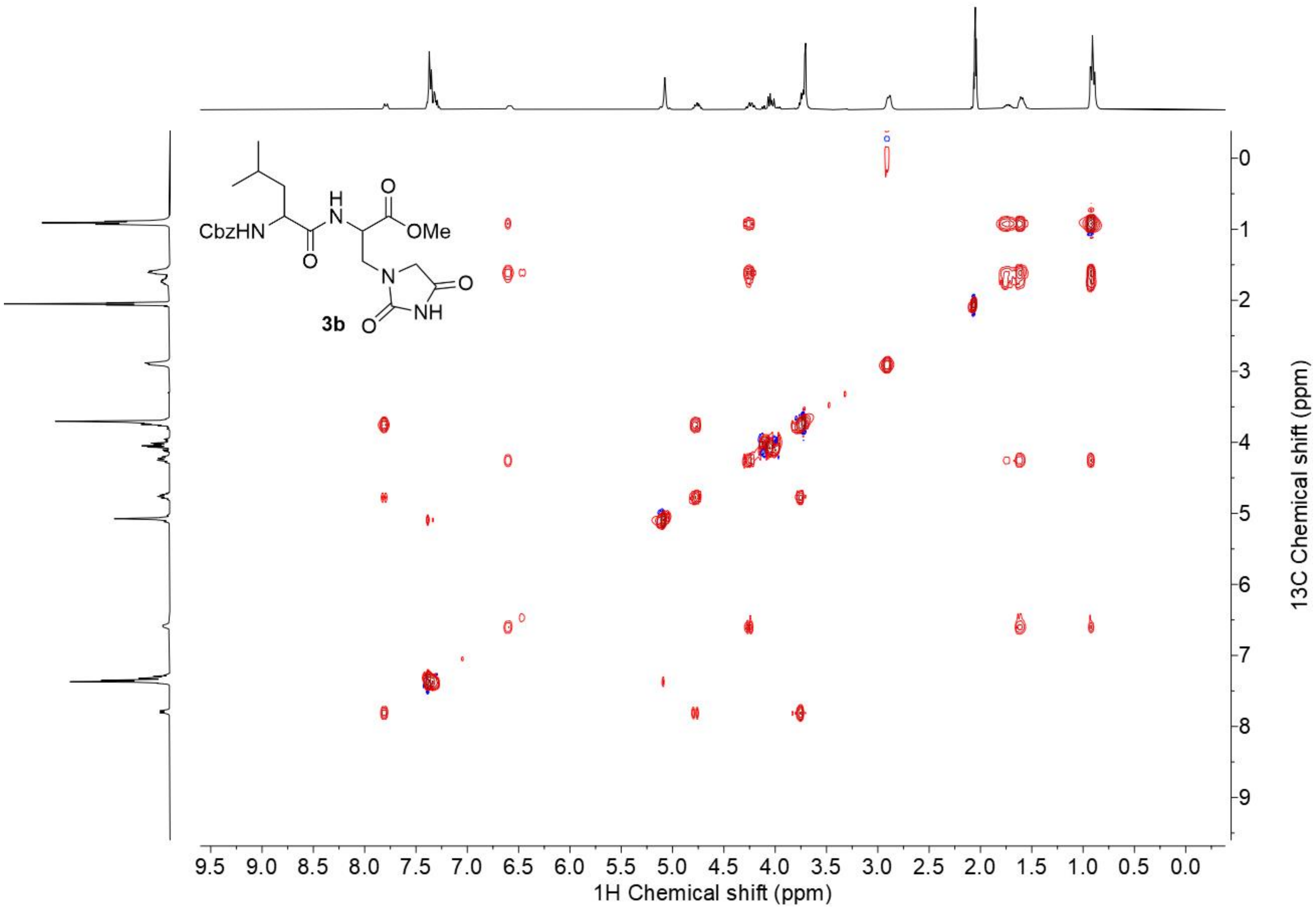

Figure SI10. ^{1}H-^{1}H TOCSY NMR (^{1}H 300 MHz, acetone-d_6) of compound **3b**.

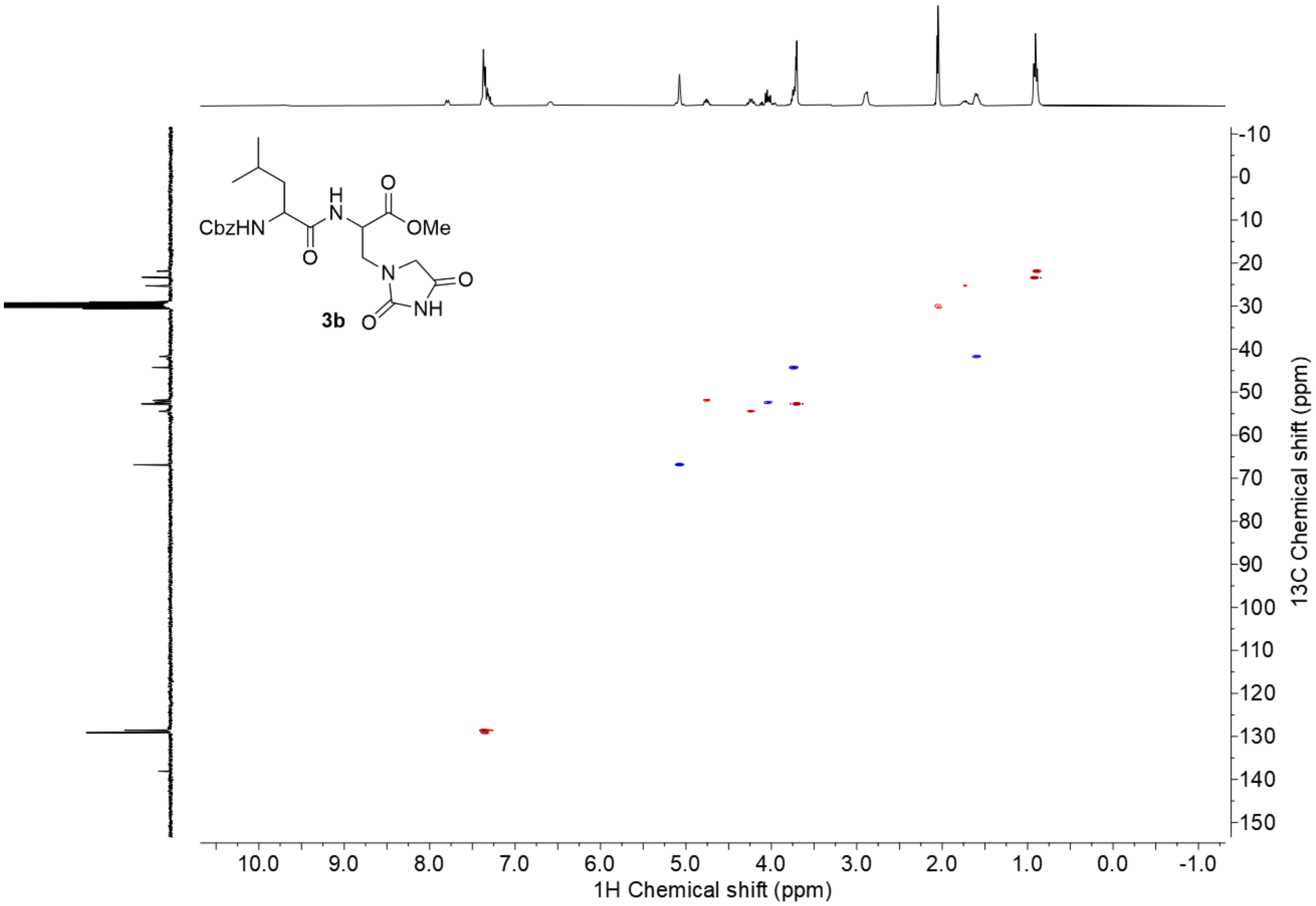

Figure SI11. ^{1}H-^{13}C HSQC-ED NMR (^{1}H 300 MHz, acetone-d_6) of compound **3b**.

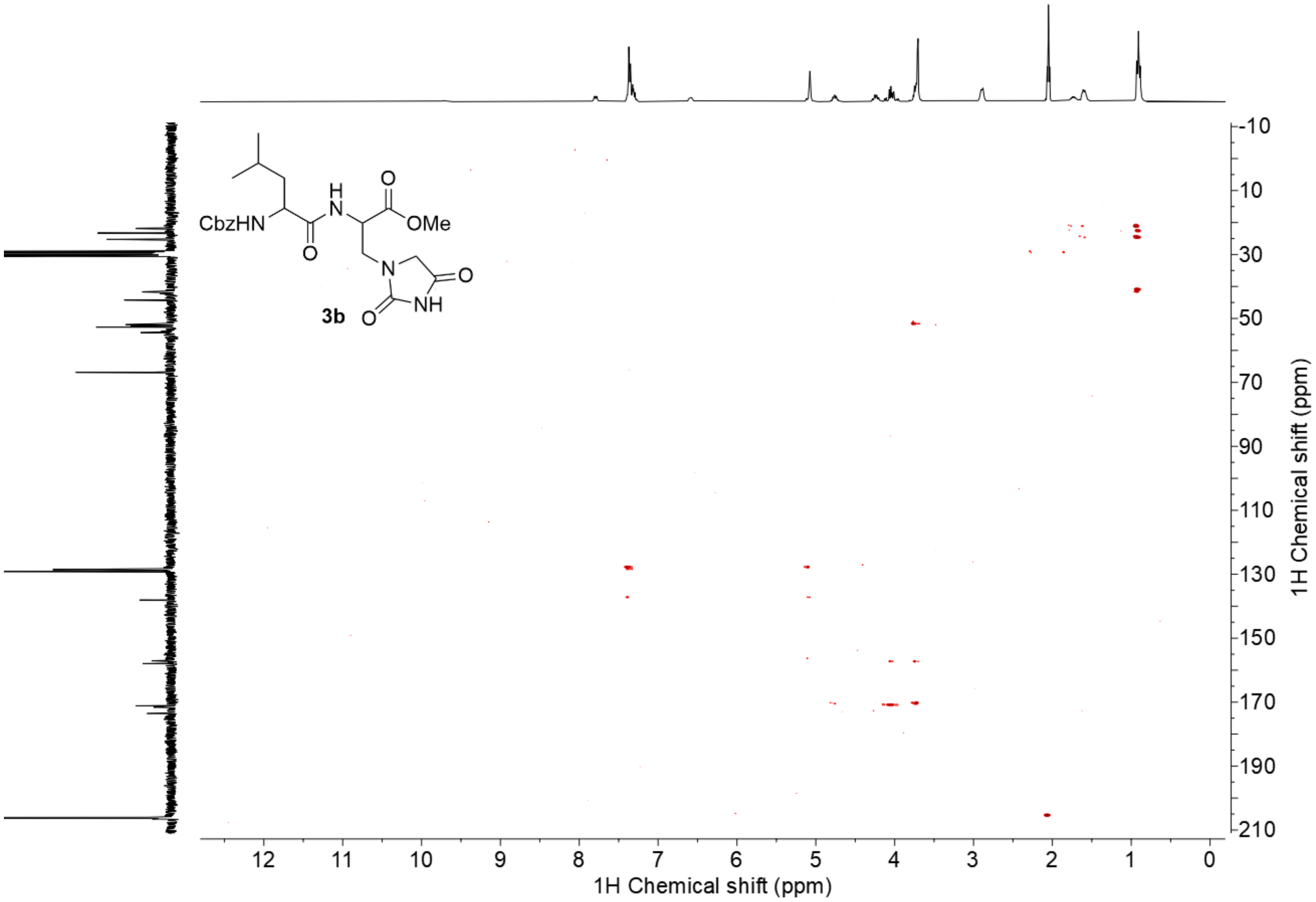

Figure SI12. ^{1}H-^{13}C HMBC NMR (^{1}H 300 MHz, acetone-d_6) of compound **3b**.

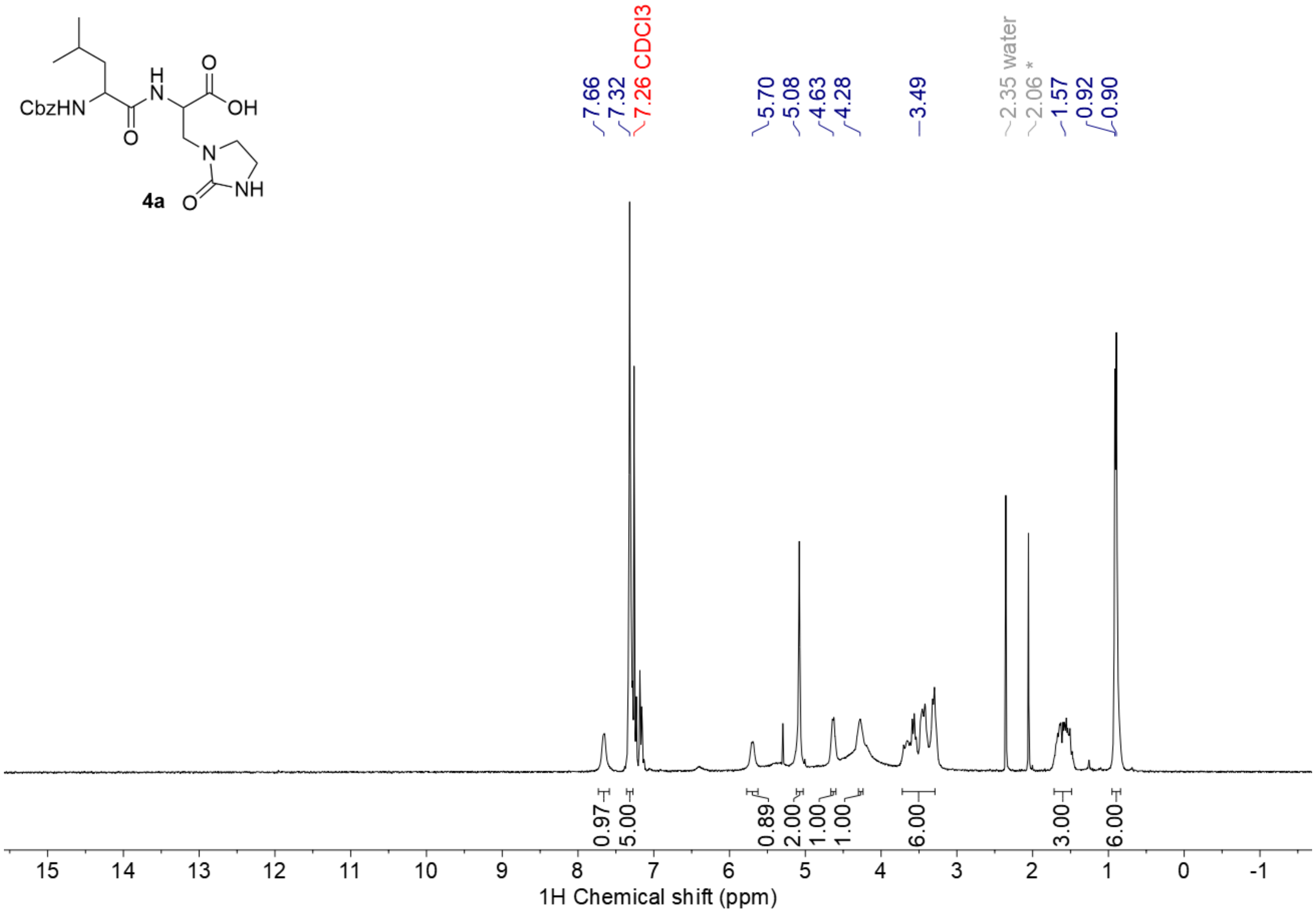

Figure SI13. ^{1}H NMR (300 MHz, $CDCl_3$) of compound **4a**; * – impurity.

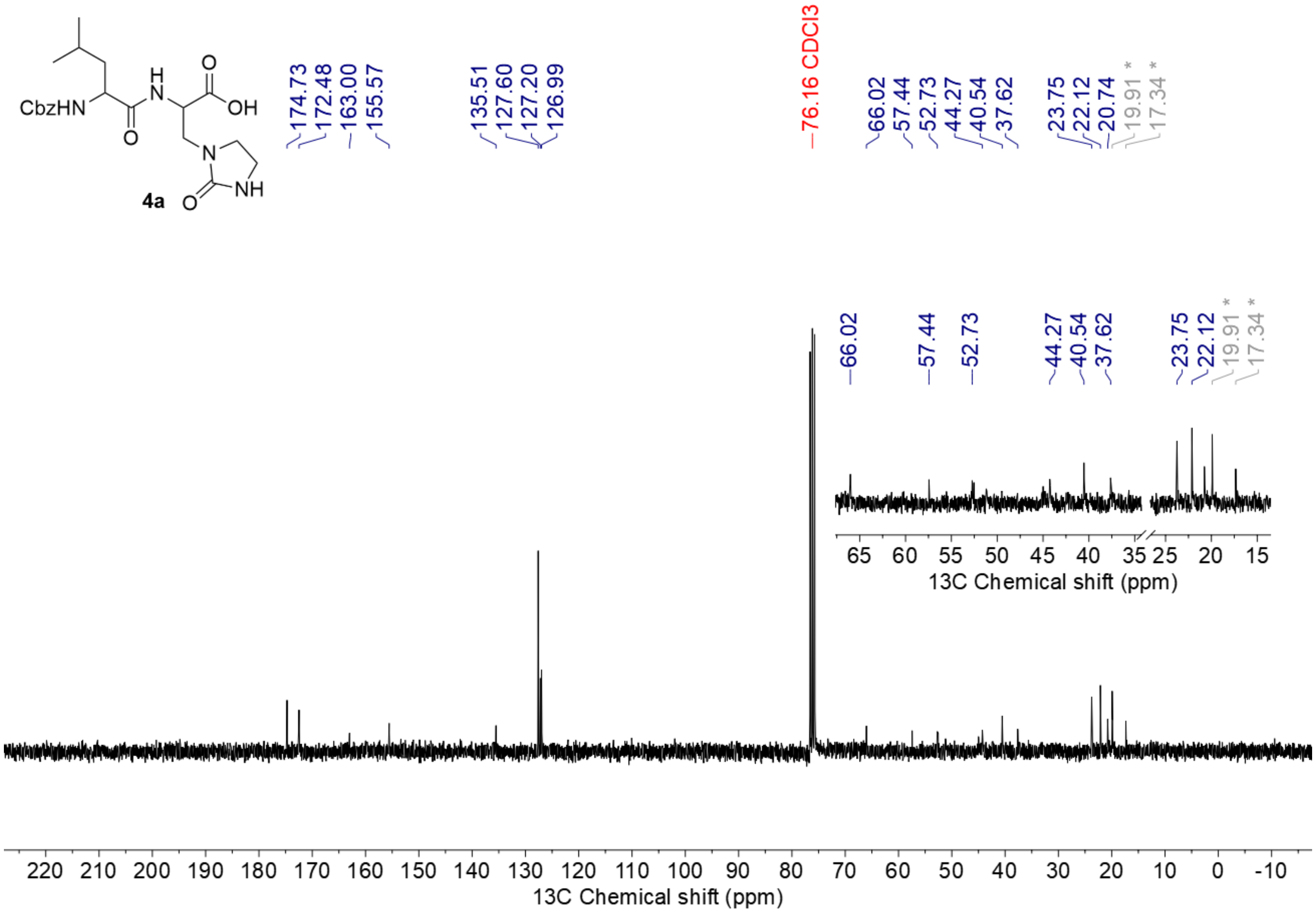

Figure SI14. ^{13}C NMR (75 MHz, $CDCl_3$) of compound **4a**; * – impurities.

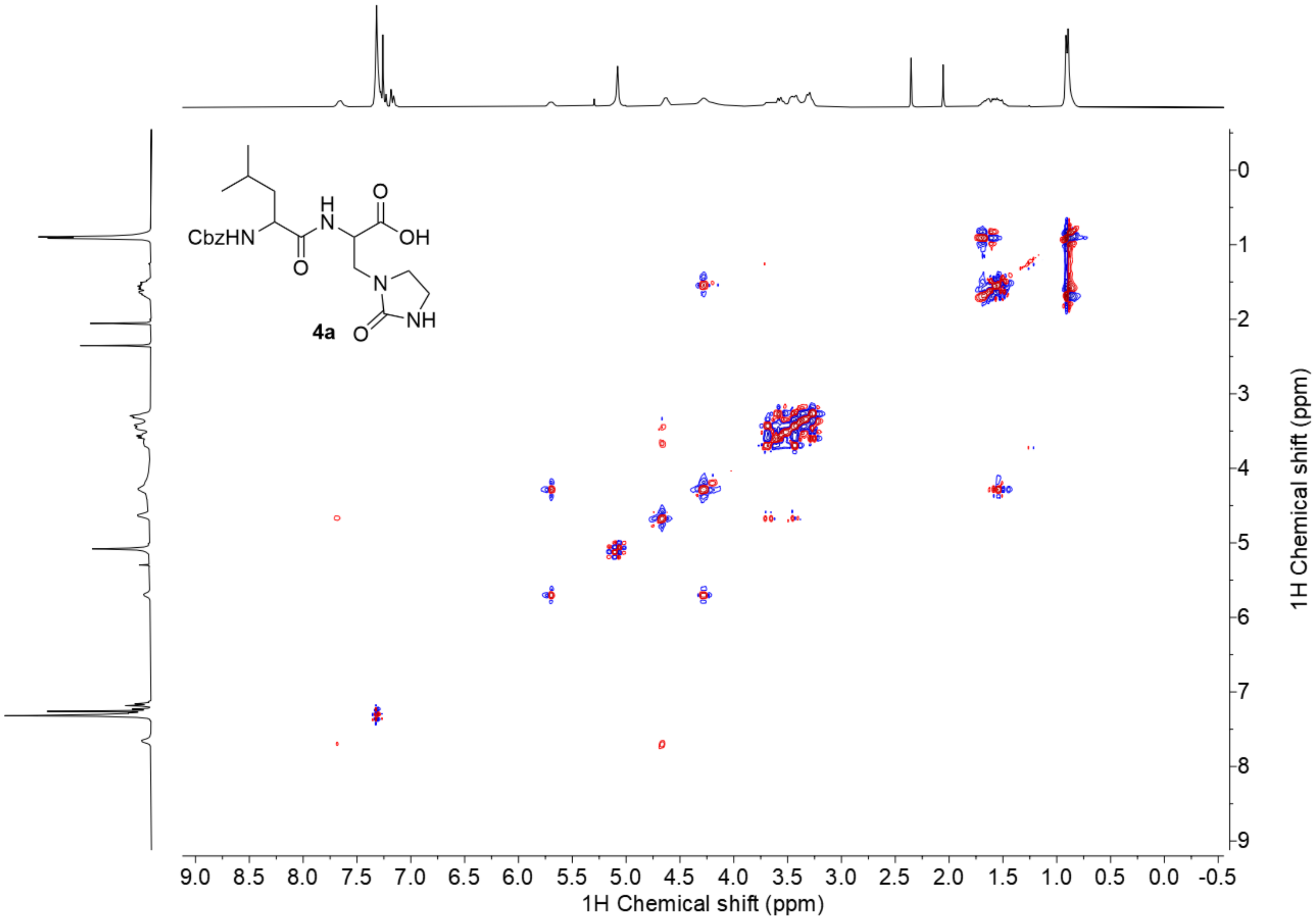

Figure SI15. ^{1}H-^{1}H COSY NMR (^{1}H 300 MHz, $CDCl_3$) of compound **4a**.

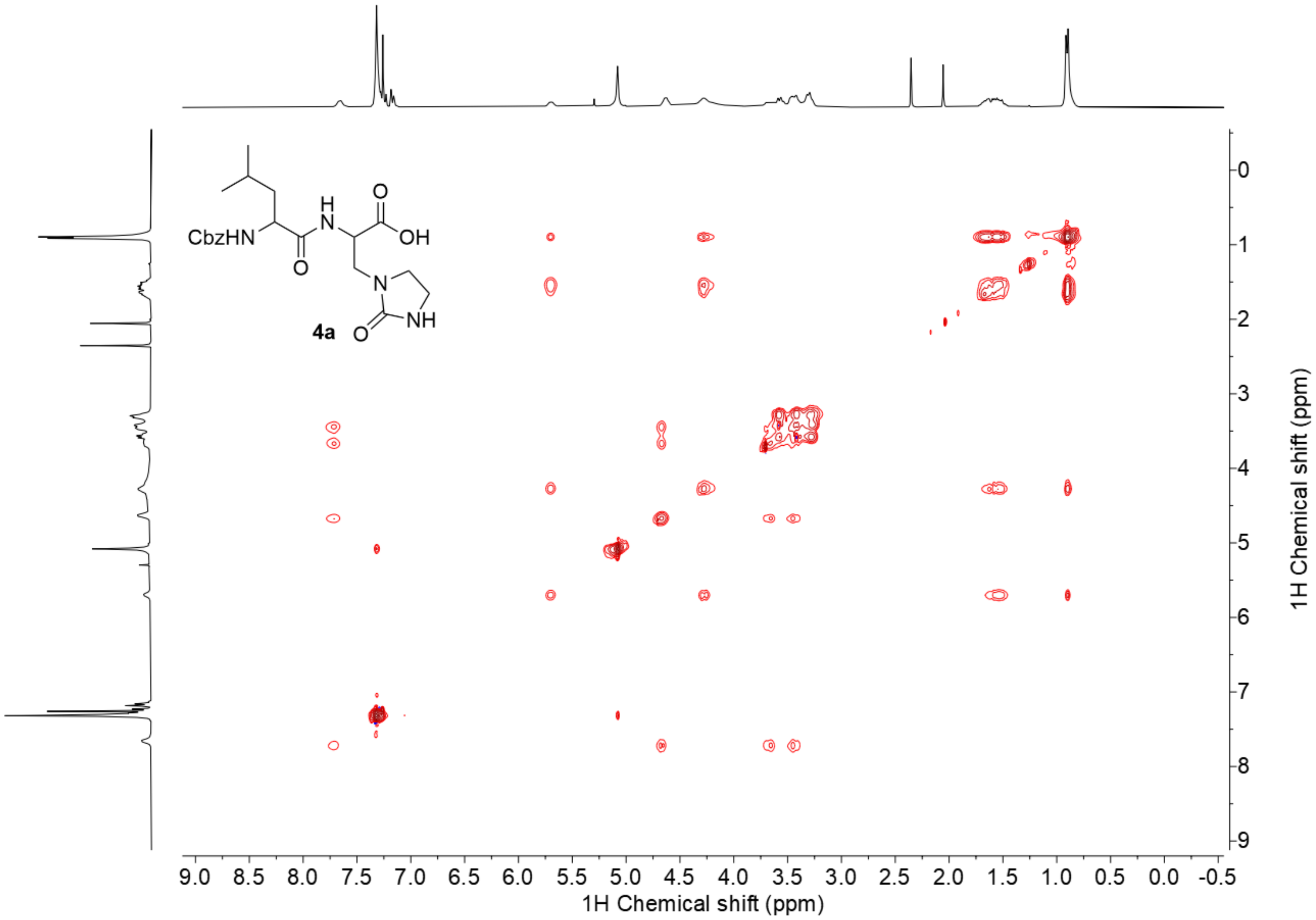

Figure SI16. ^{1}H-^{1}H TOCSY NMR (^{1}H 300 MHz, $CDCl_3$) of compound **4a**.

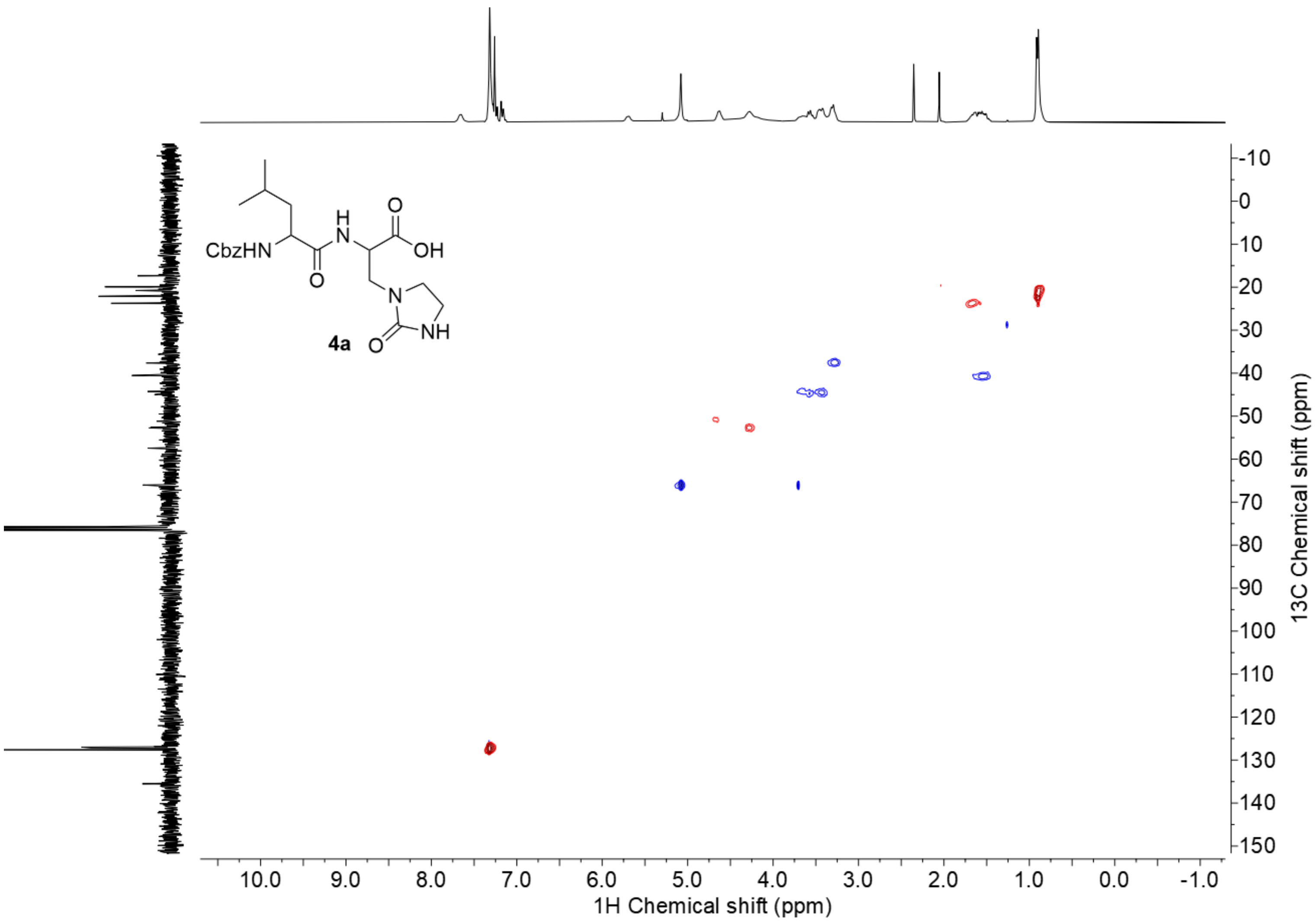

Figure SI17. ^{1}H-^{13}C HSQC-ED NMR (^{1}H 300 MHz, $CDCl_3$) of compound **4a**.

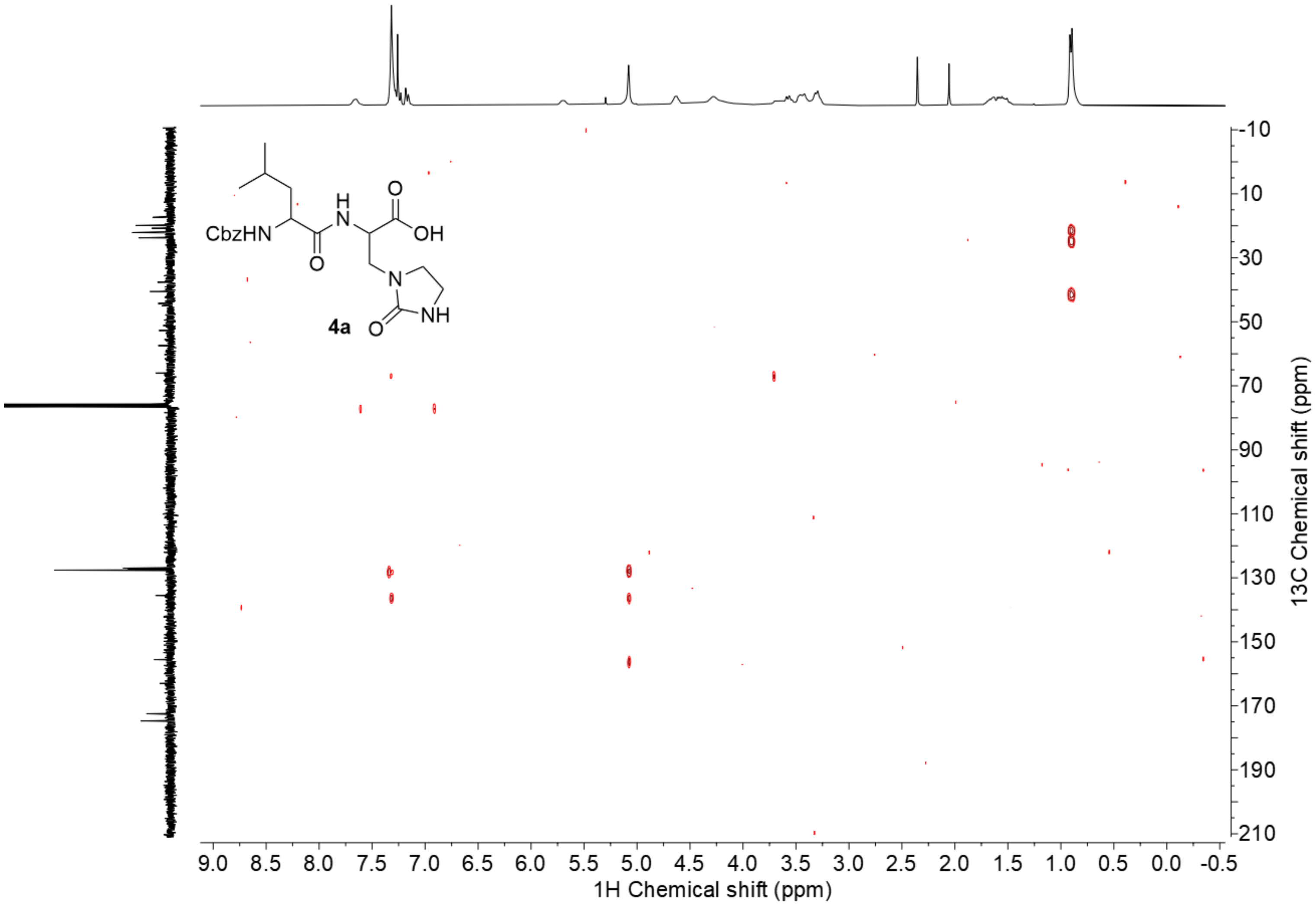

Figure SI18. ^{1}H-^{13}C HMBC NMR (^{1}H 300 MHz, $CDCl_3$) of compound **4a**.

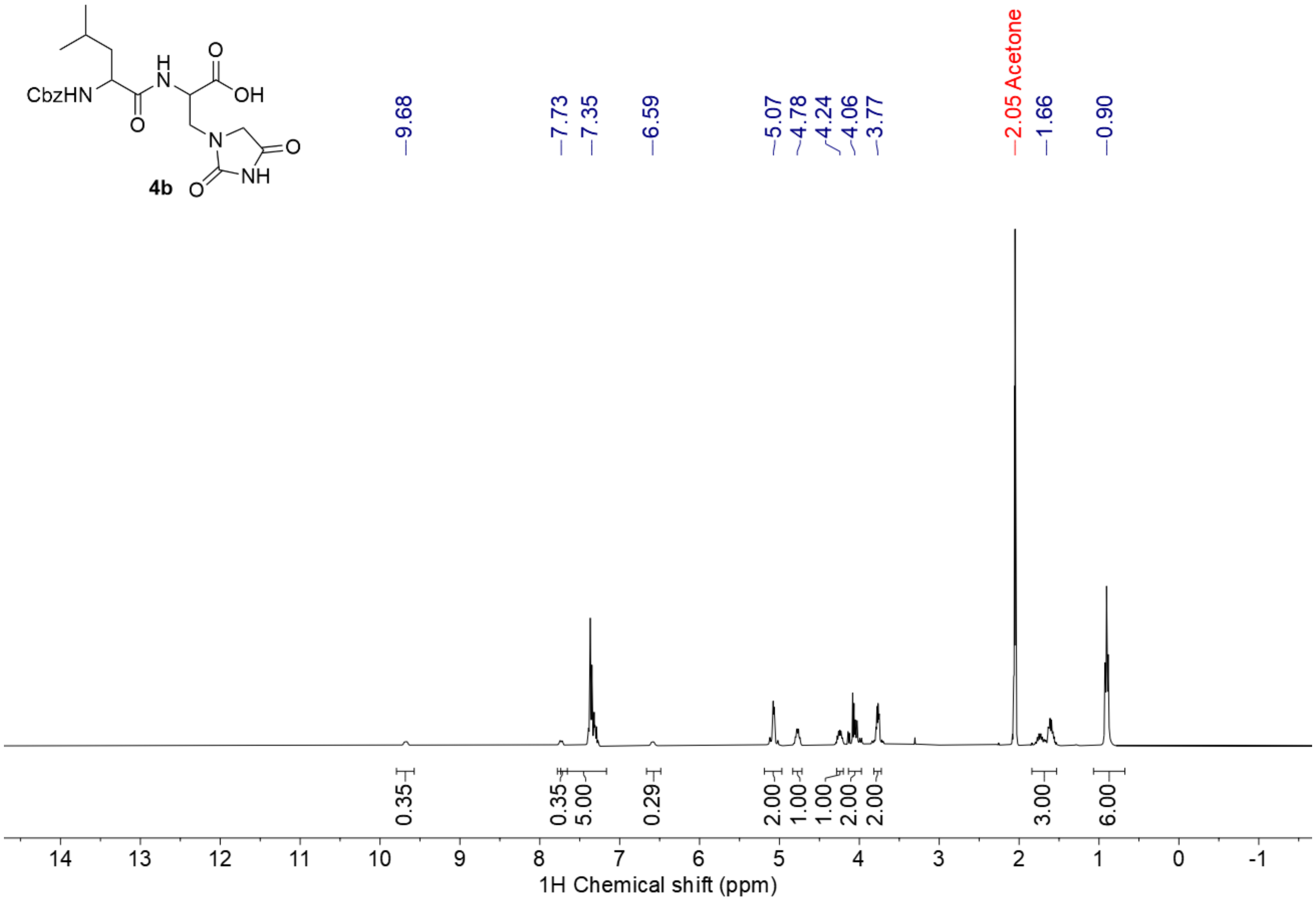

Figure SI19. ^{1}H NMR (300 MHz, acetone-d_6) of compound **4b**.

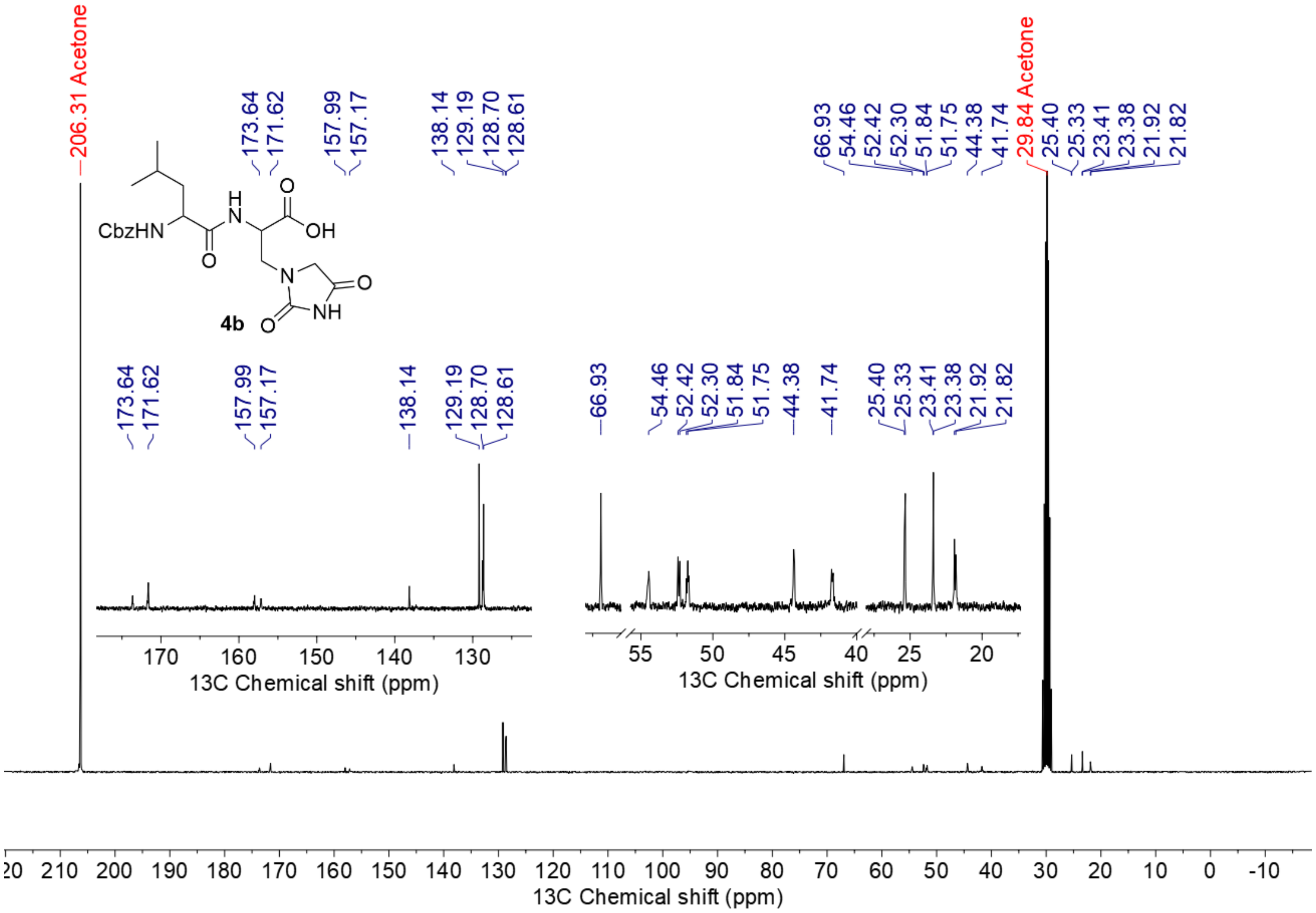

Figure SI20. ^{13}C NMR (75 MHz, acetone-d_6) of compound **4b**.

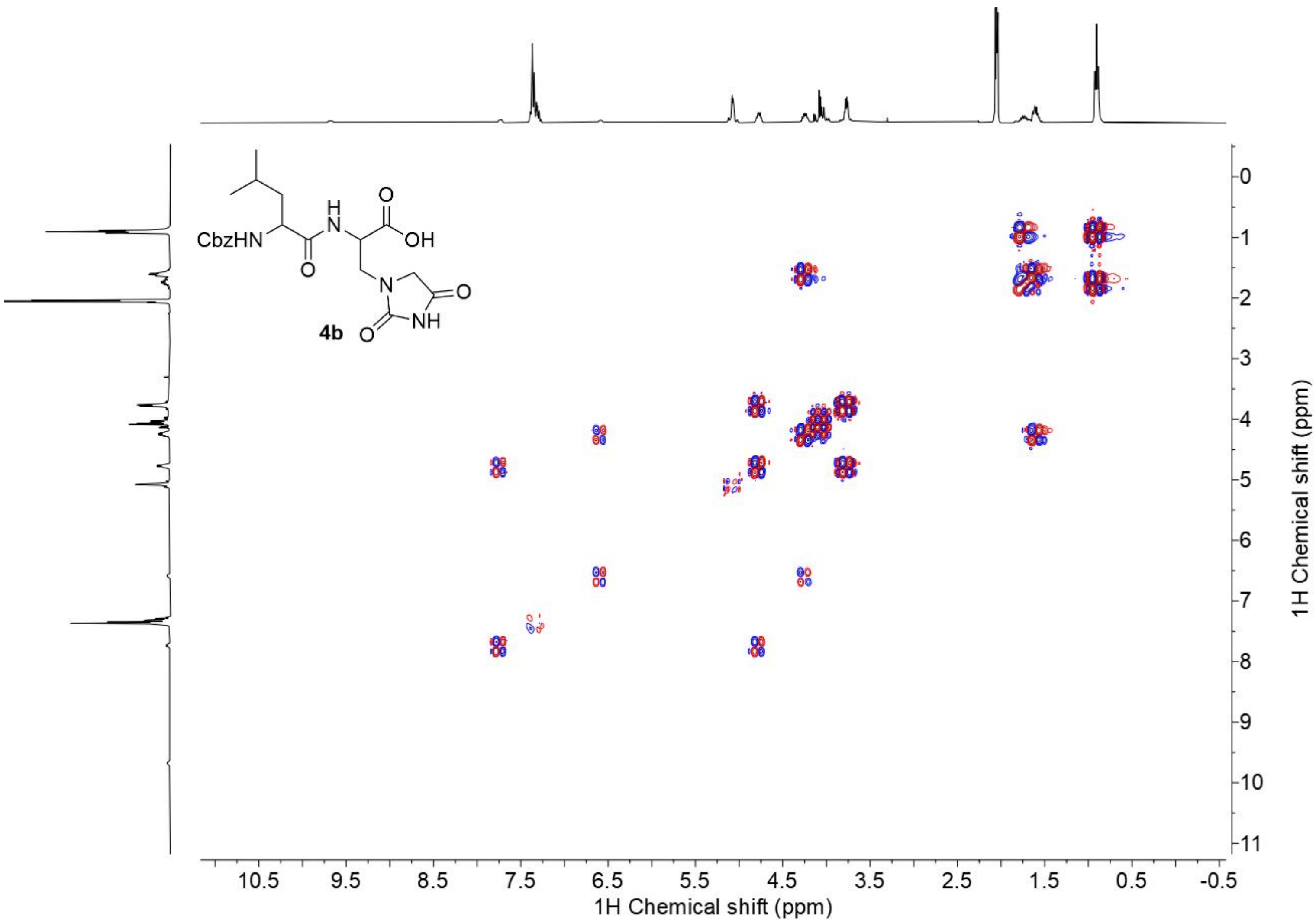

Figure SI21. ^{1}H-^{1}H COSY NMR (^{1}H 300 MHz, acetone-d_6) of compound **4b**.

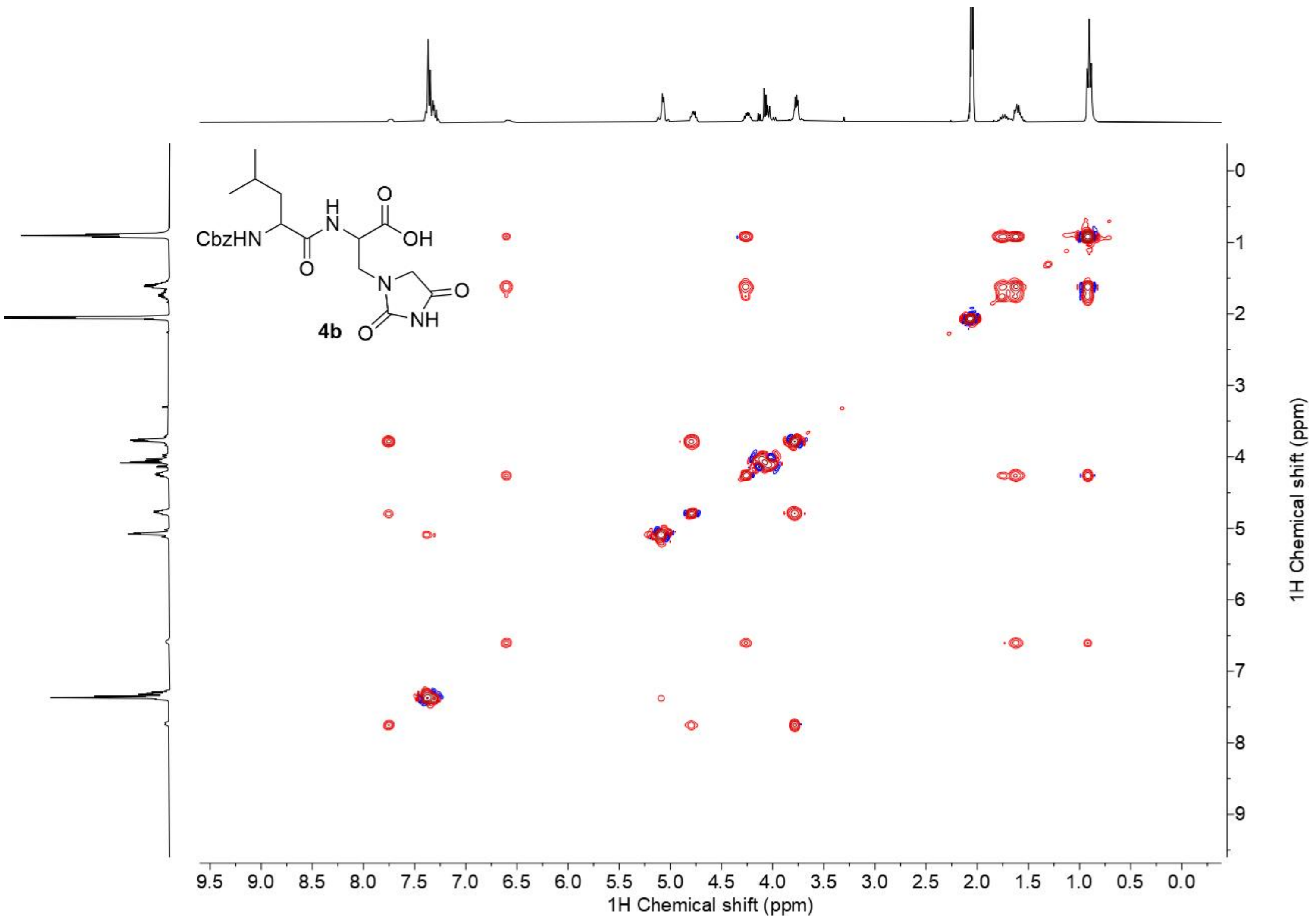

Figure SI22. ^{1}H-^{1}H TOCSY NMR (^{1}H 300 MHz, acetone-d_6) of compound **4b**.

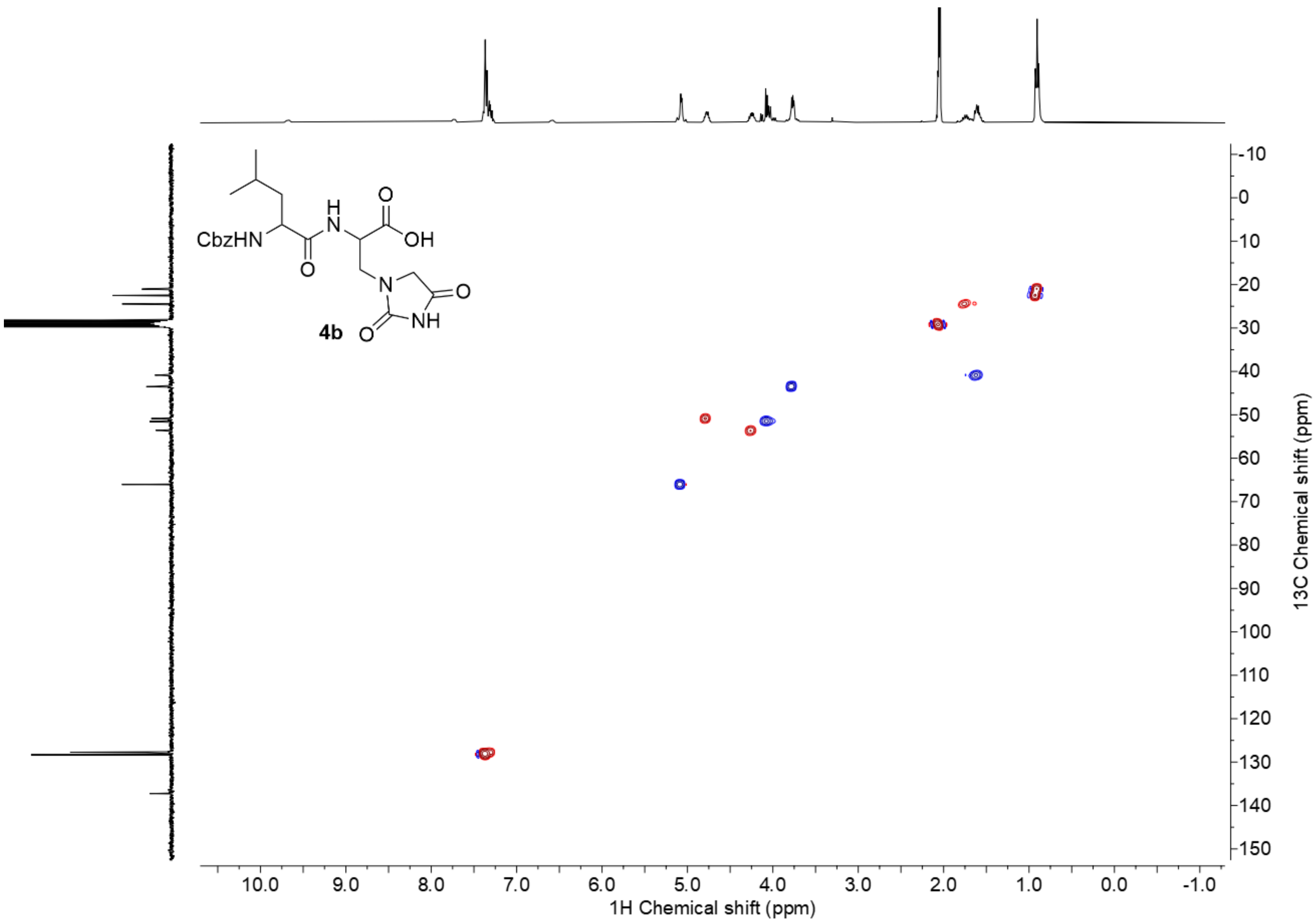

Figure SI23. ^{1}H-^{13}C HSQC-ED NMR (^{1}H 300 MHz, acetone-d_6) of compound **4b**.

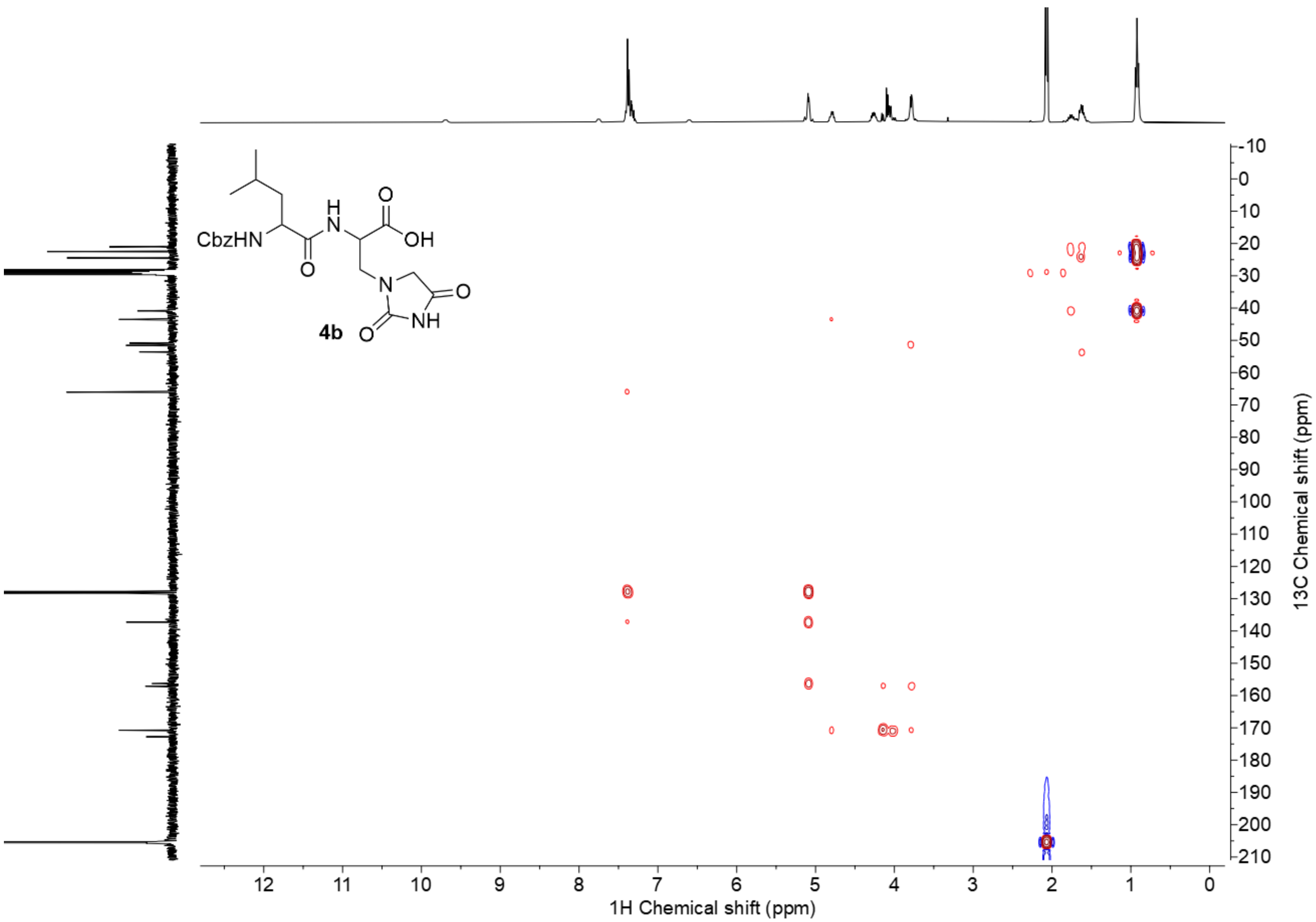

Figure SI24. ^{1}H-^{13}C HMBC NMR (^{1}H 300 MHz, acetone-d_6) of compound **4b**.

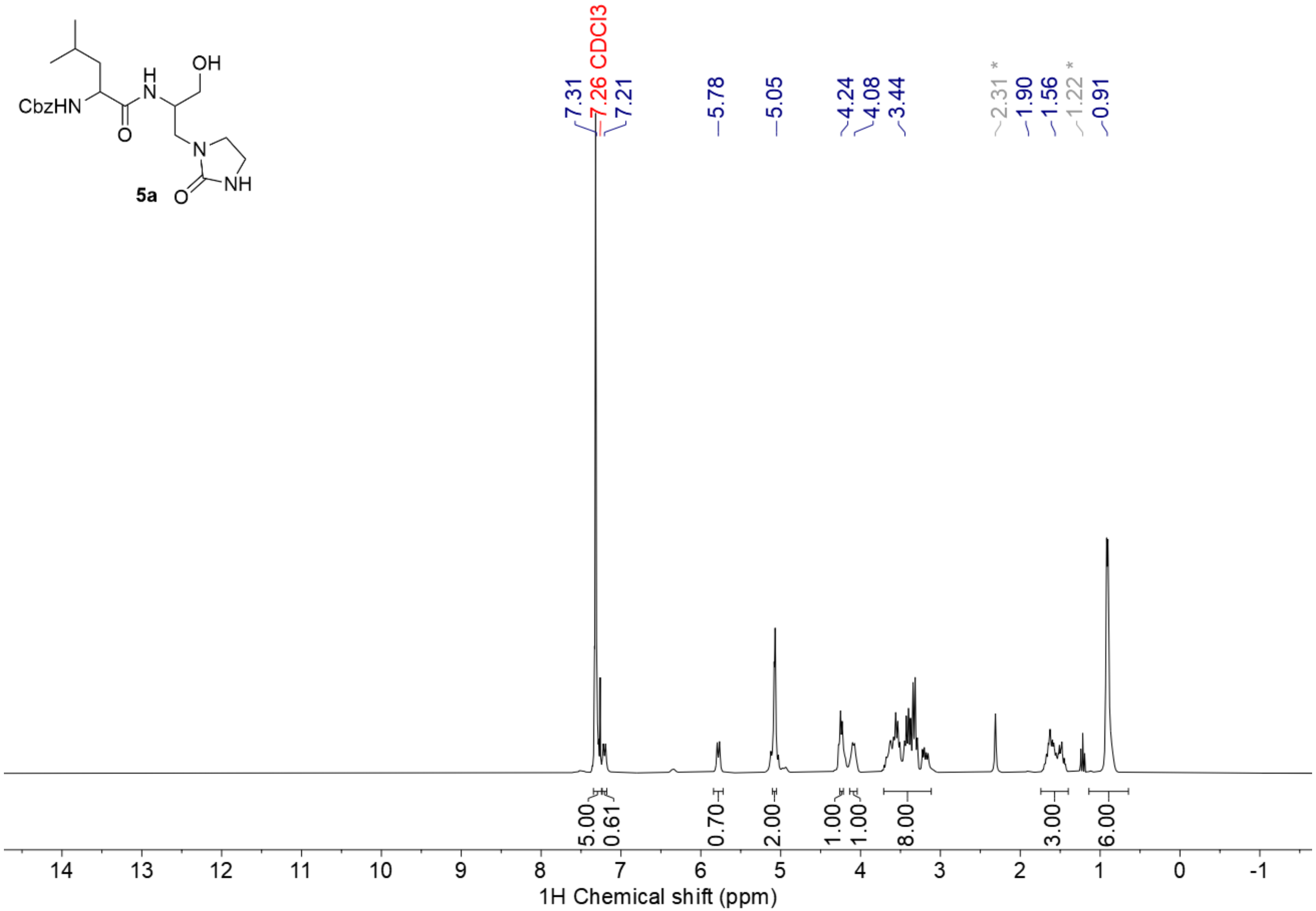

Figure SI25. ^{1}H NMR (300 MHz, $CDCl_3$) of compound **5a**; * – impurities.

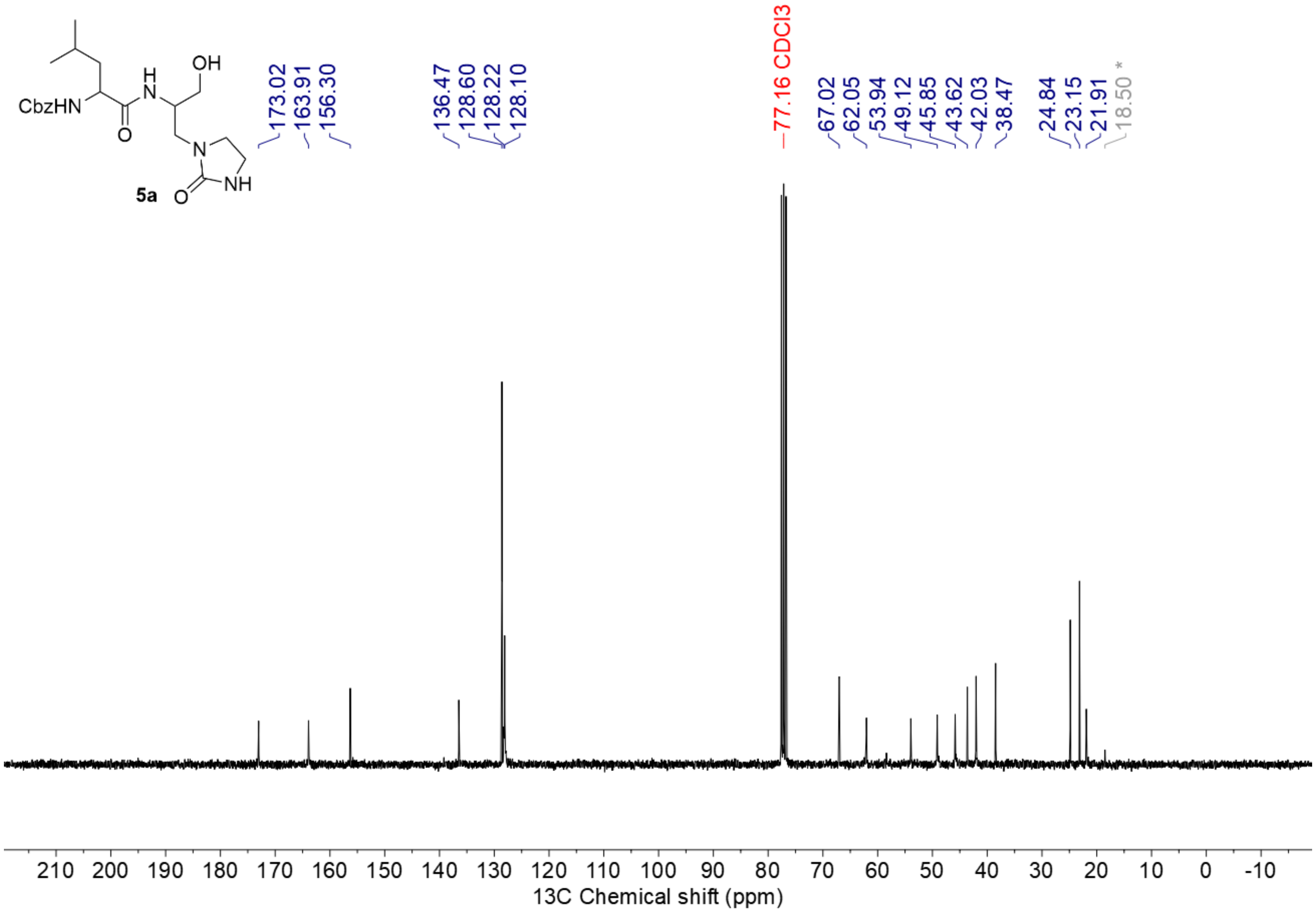

Figure SI26. ^{13}C NMR (75 MHz, $CDCl_3$) of compound **5a**; * – impurities.

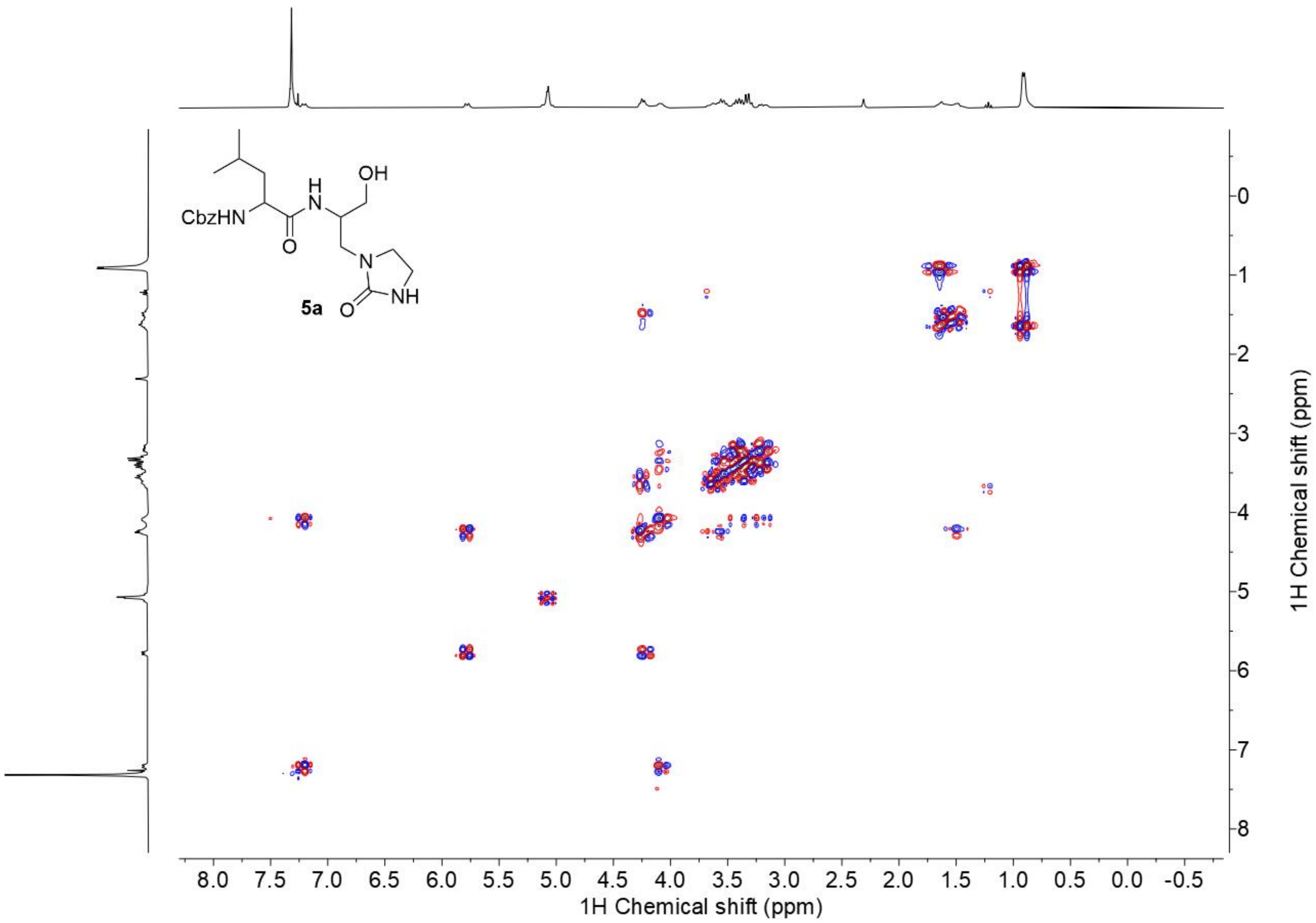

Figure SI27. ^{1}H-^{1}H COSY NMR (^{1}H 300 MHz, $CDCl_3$) of compound **5a**.

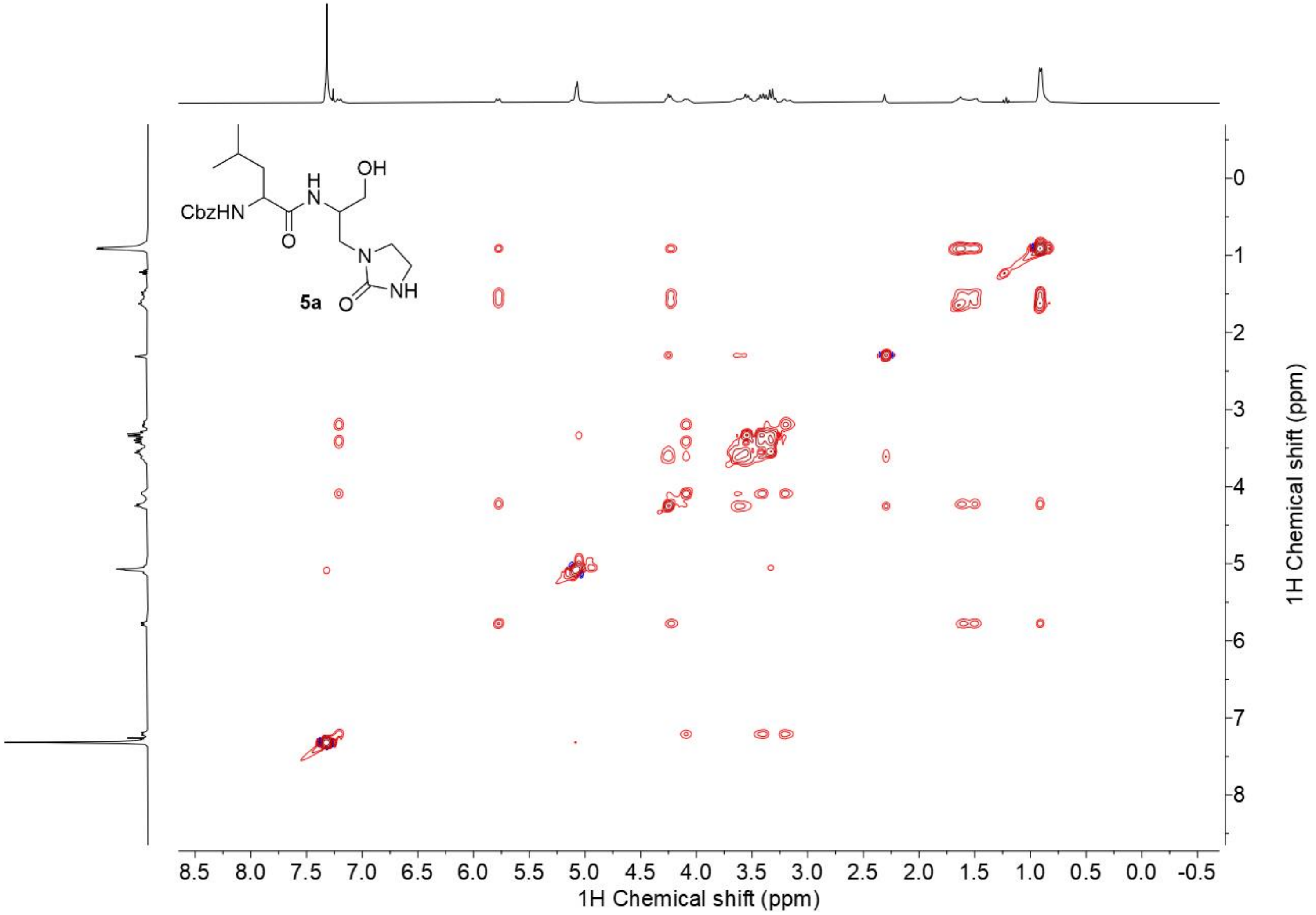

Figure SI28. ^{1}H-^{1}H TOCSY NMR (^{1}H 300 MHz, $CDCl_3$) of compound **5a**.

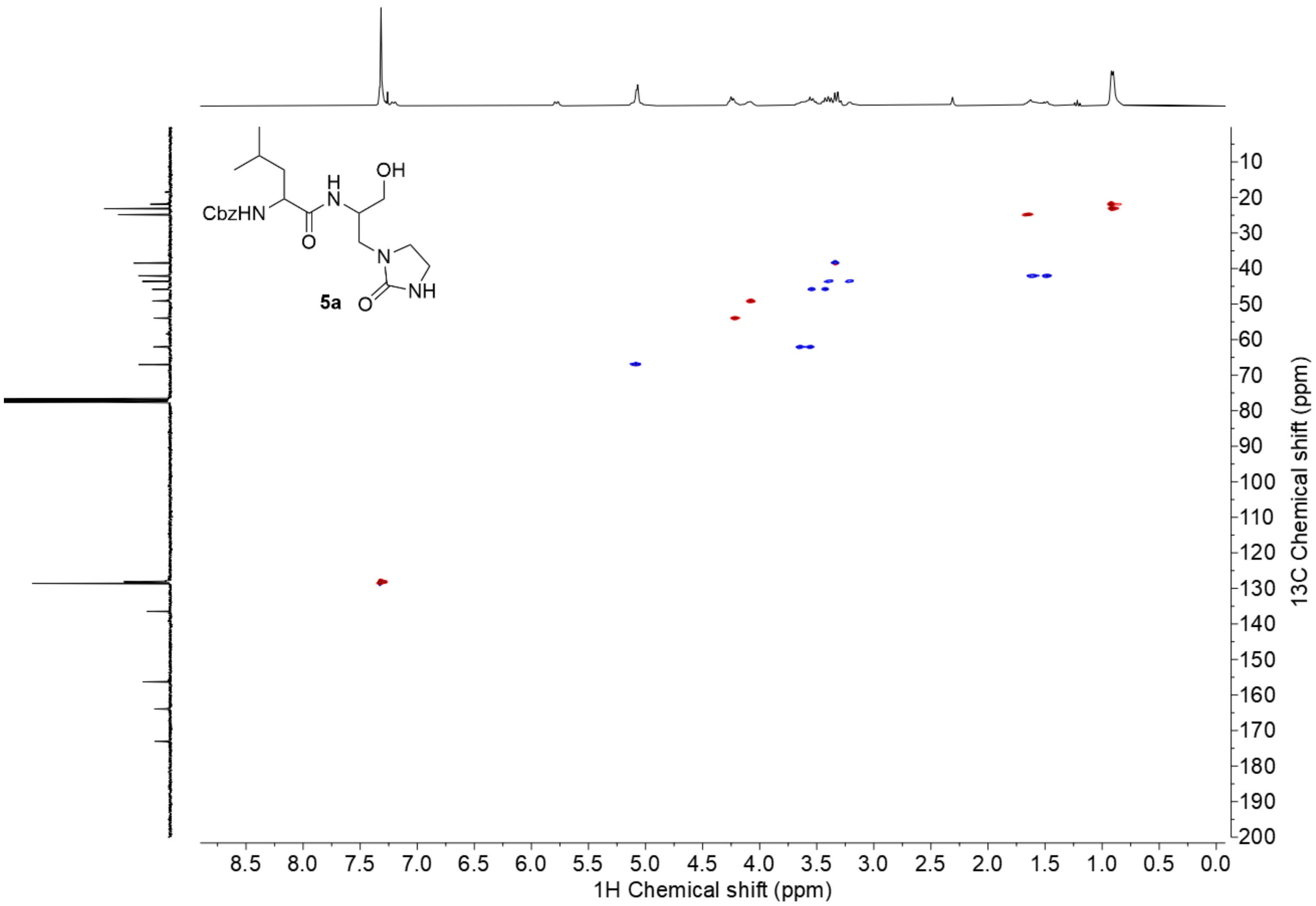

Figure SI29. ^{1}H-^{13}C HSQC-ED NMR (^{1}H 300 MHz, $CDCl_3$) of compound **5a**.

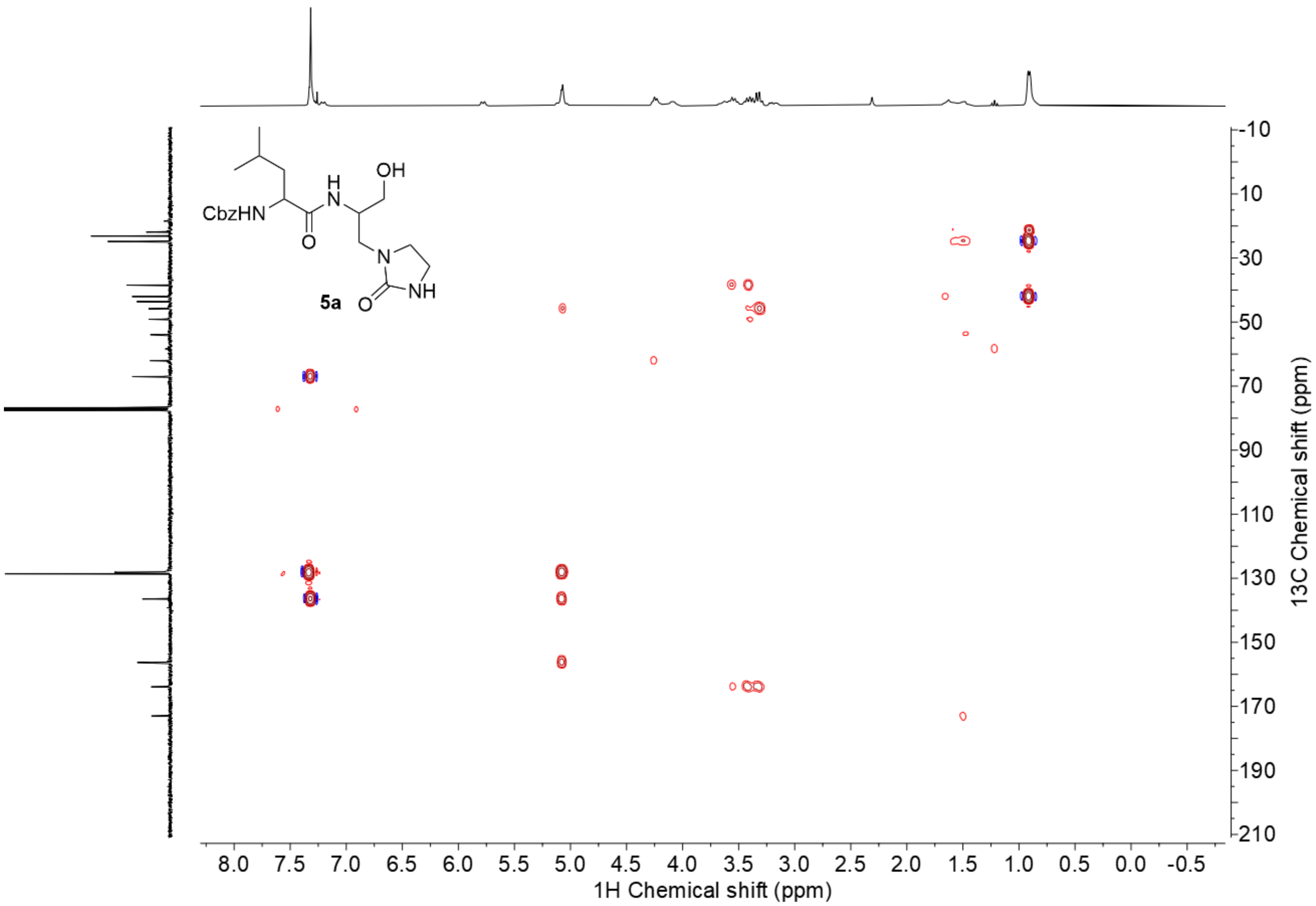

Figure SI30. ^{1}H-^{13}C HMBC NMR (^{1}H 300 MHz, $CDCl_3$) of compound **5a**.

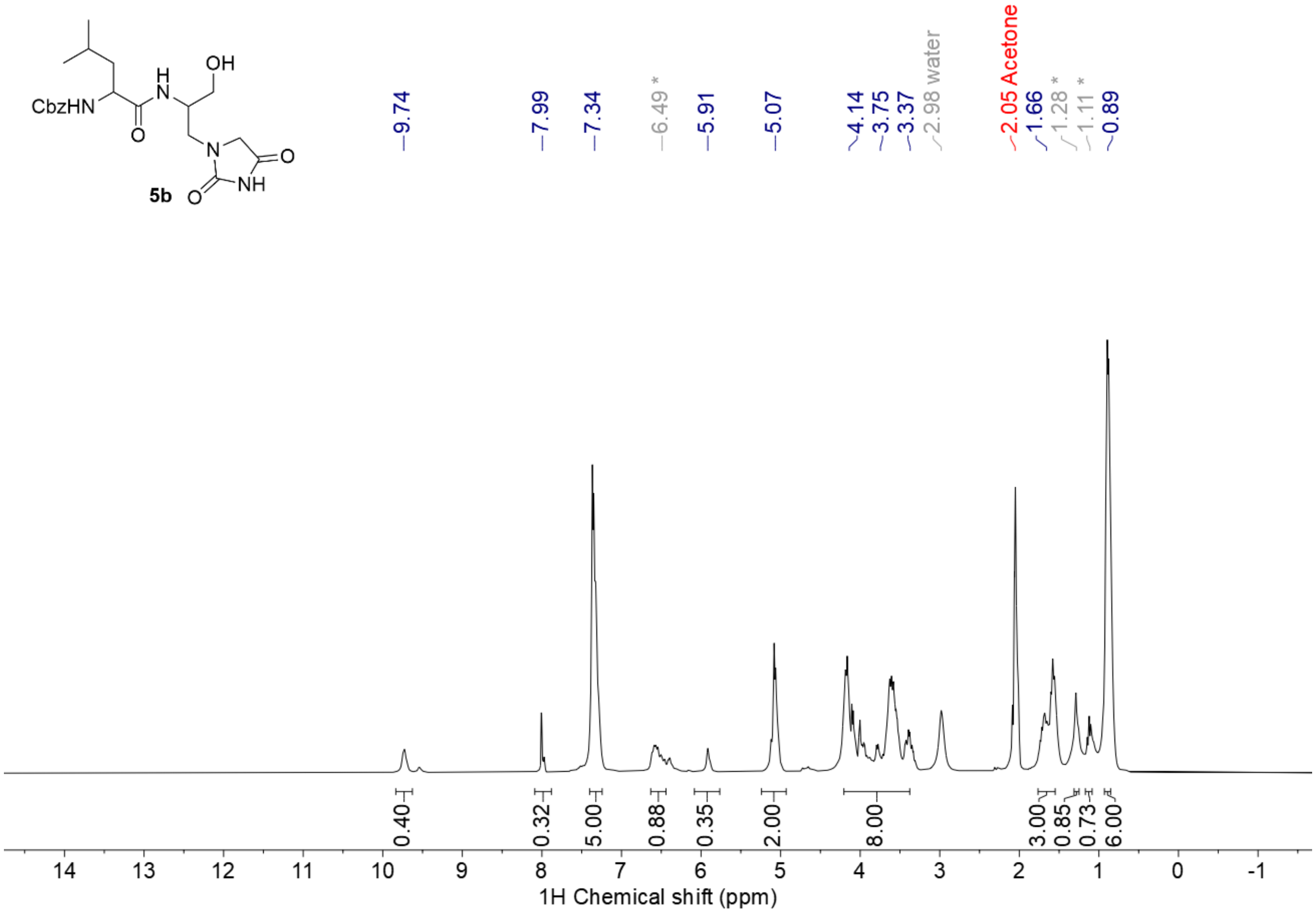

Figure SI31. ^{1}H NMR (300 MHz, acetone-d_6) of compound **5b**; * – impurities.

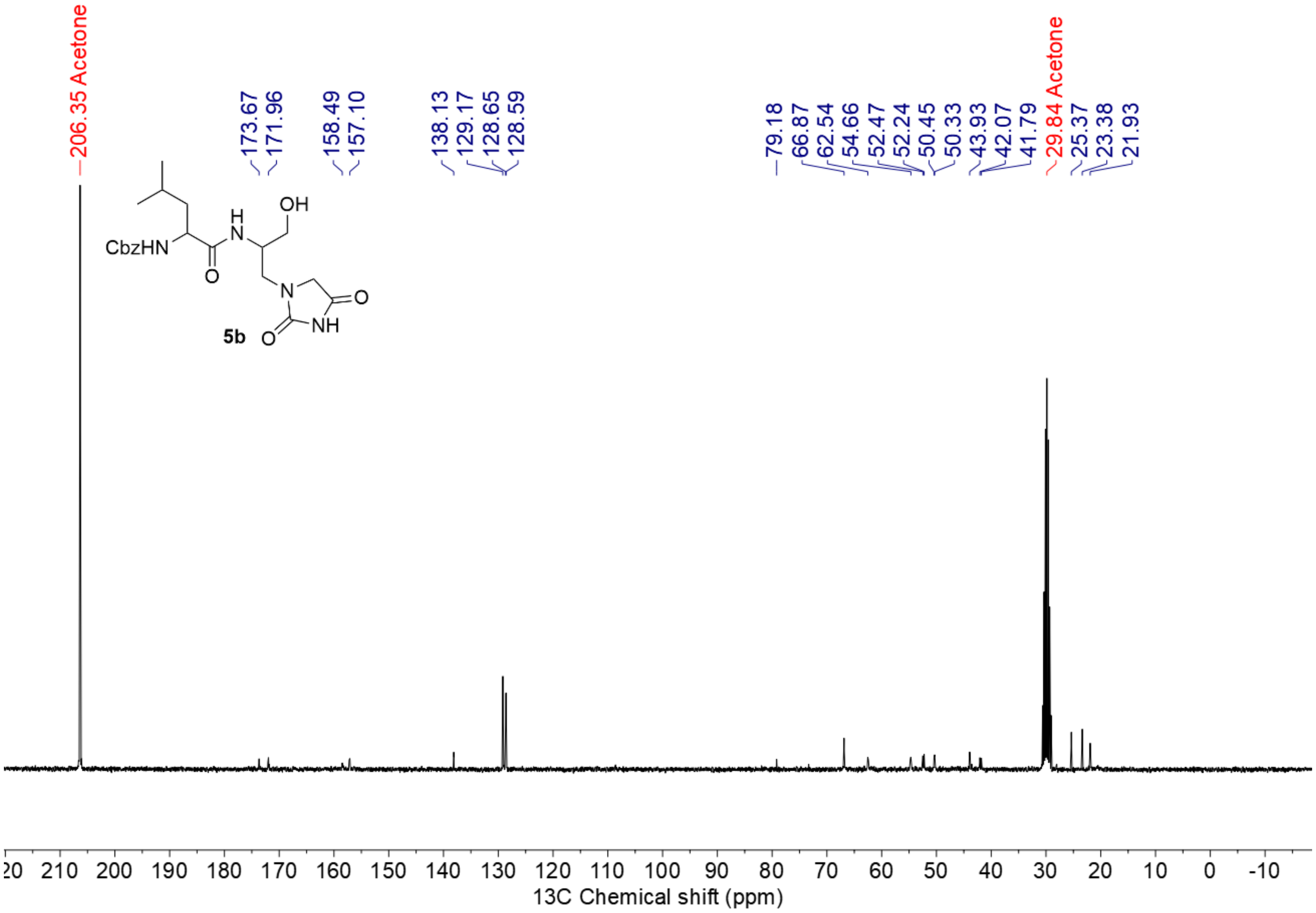

Figure SI32. ^{13}C NMR (75 MHz, acetone-d_6) of compound **5b**.

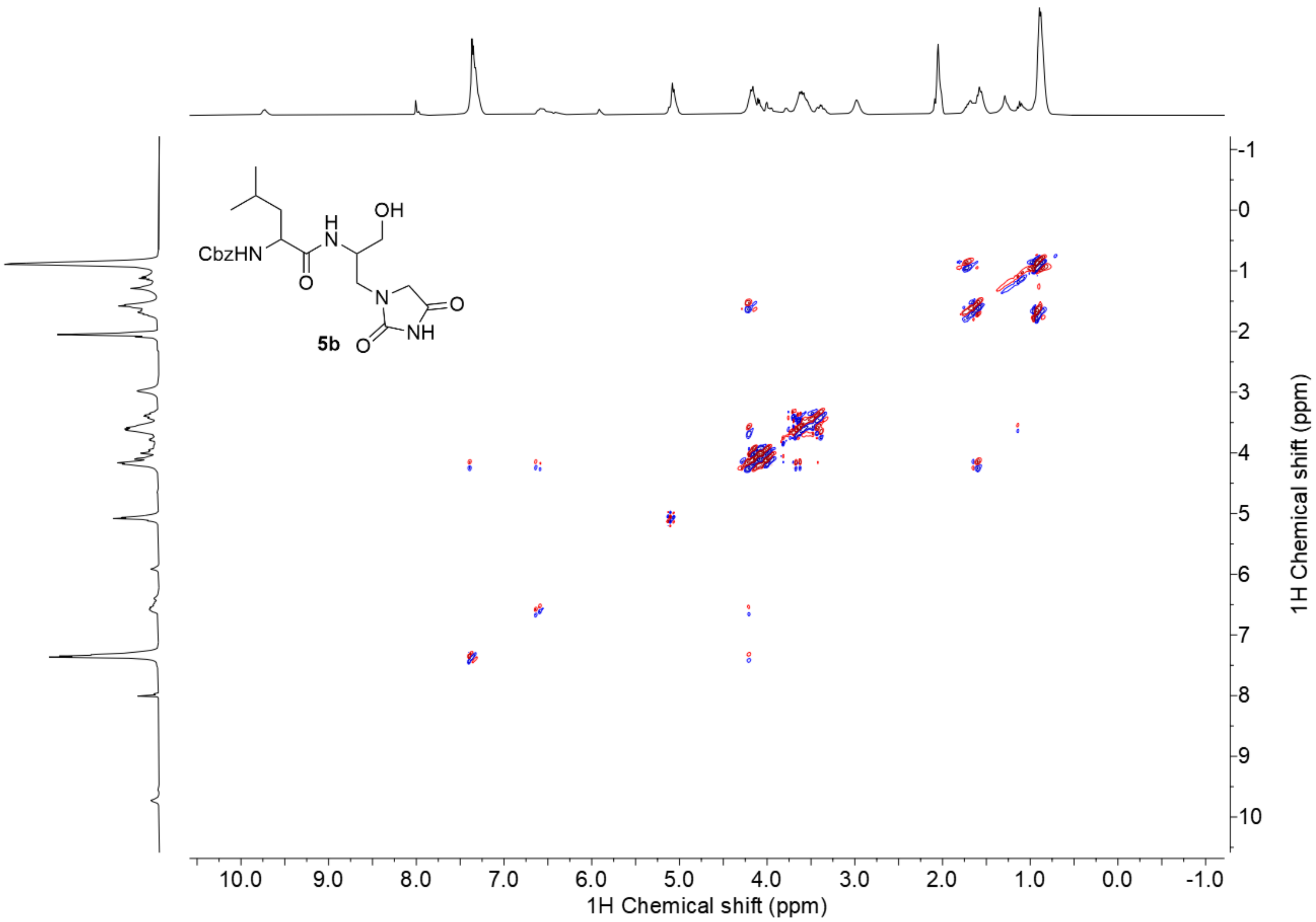

Figure SI33. ^{1}H-^{1}H COSY NMR (^{1}H 300 MHz, acetone-d_6) of compound **5b**.

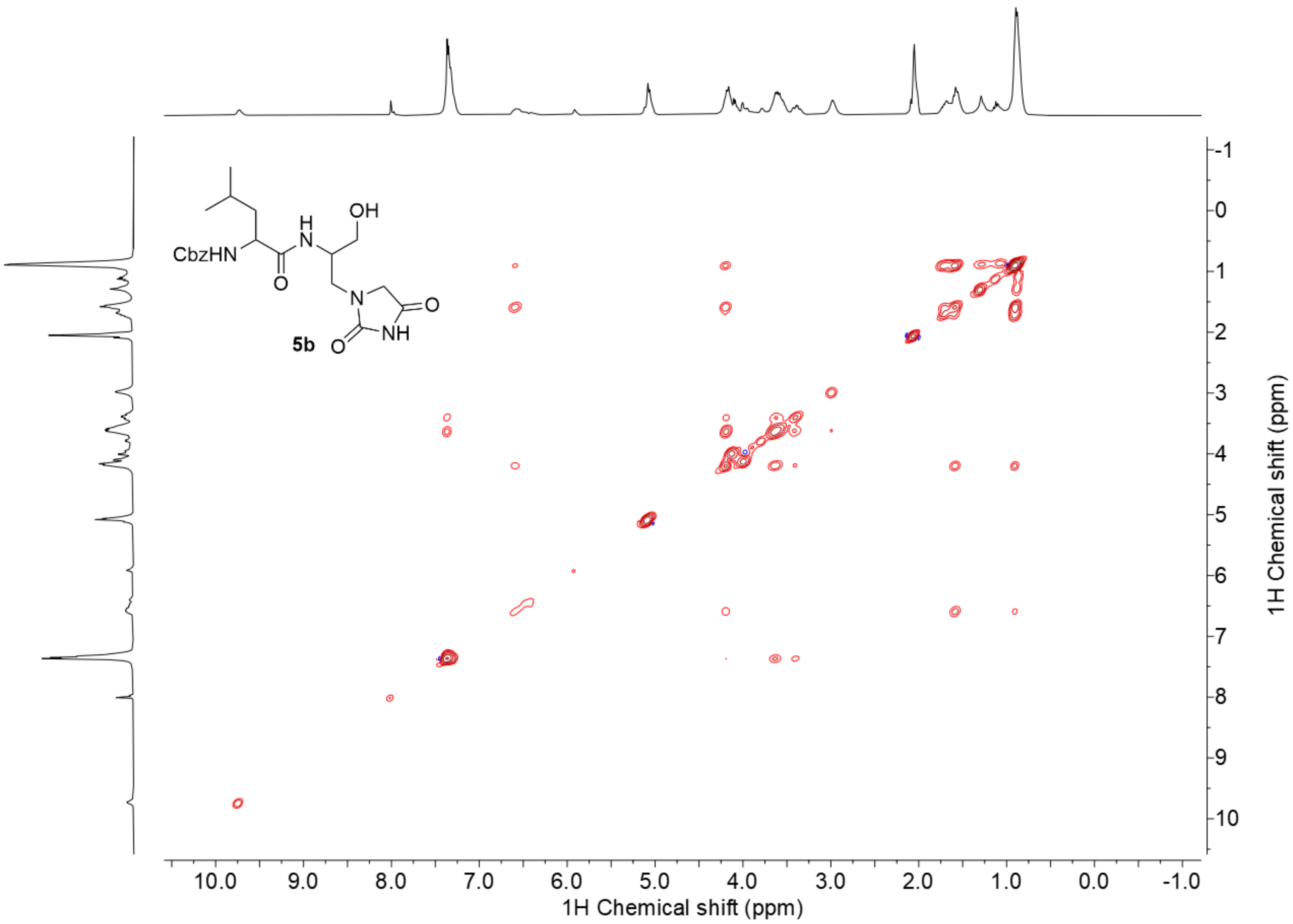

Figure SI34. ^{1}H-^{1}H TOCSY NMR (^{1}H 300 MHz, acetone-d_6) of compound **5b**.

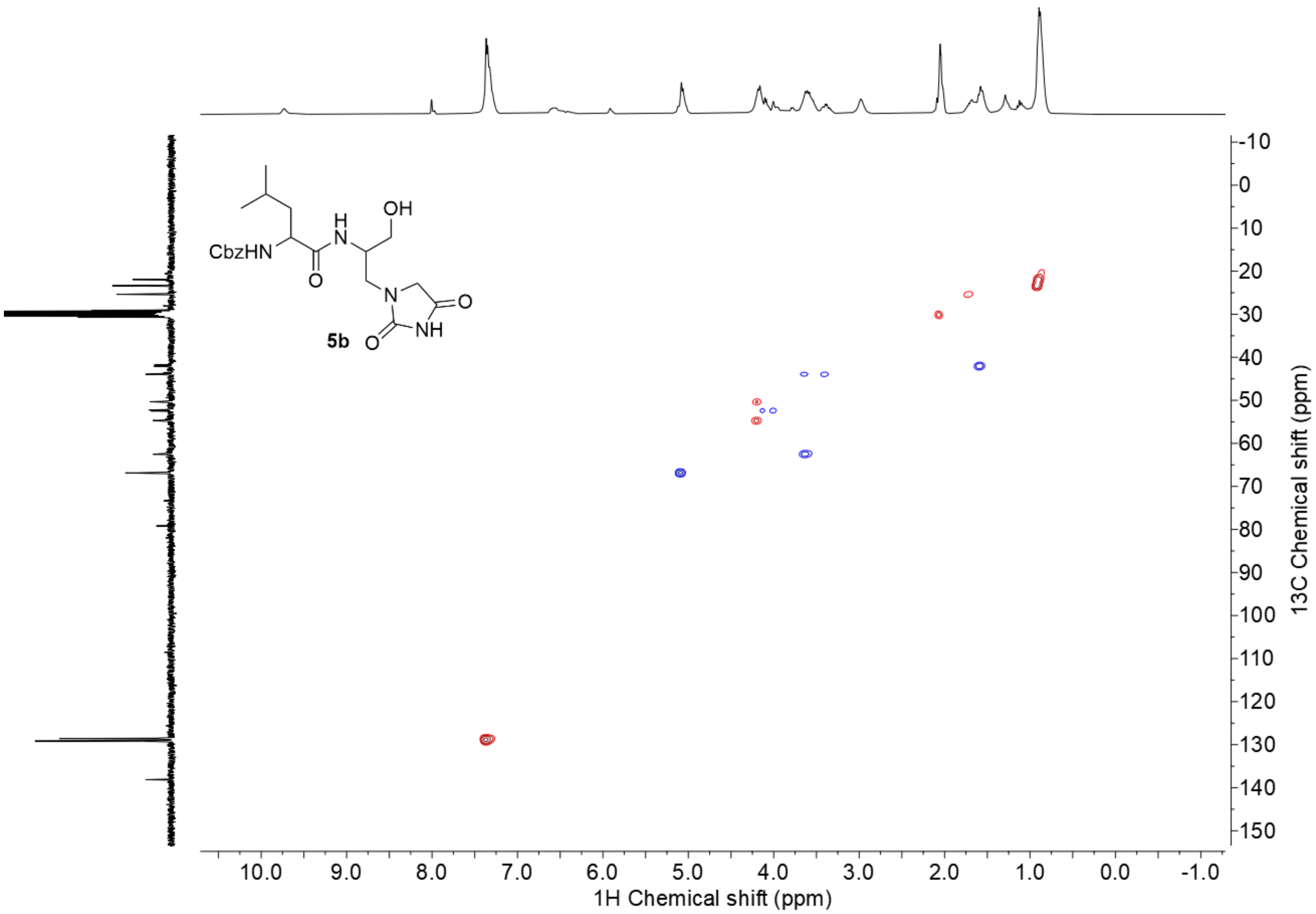

Figure SI35. ^{1}H-^{13}C HSQC-ED NMR (^{1}H 300 MHz, acetone-d_6) of compound **5b**.

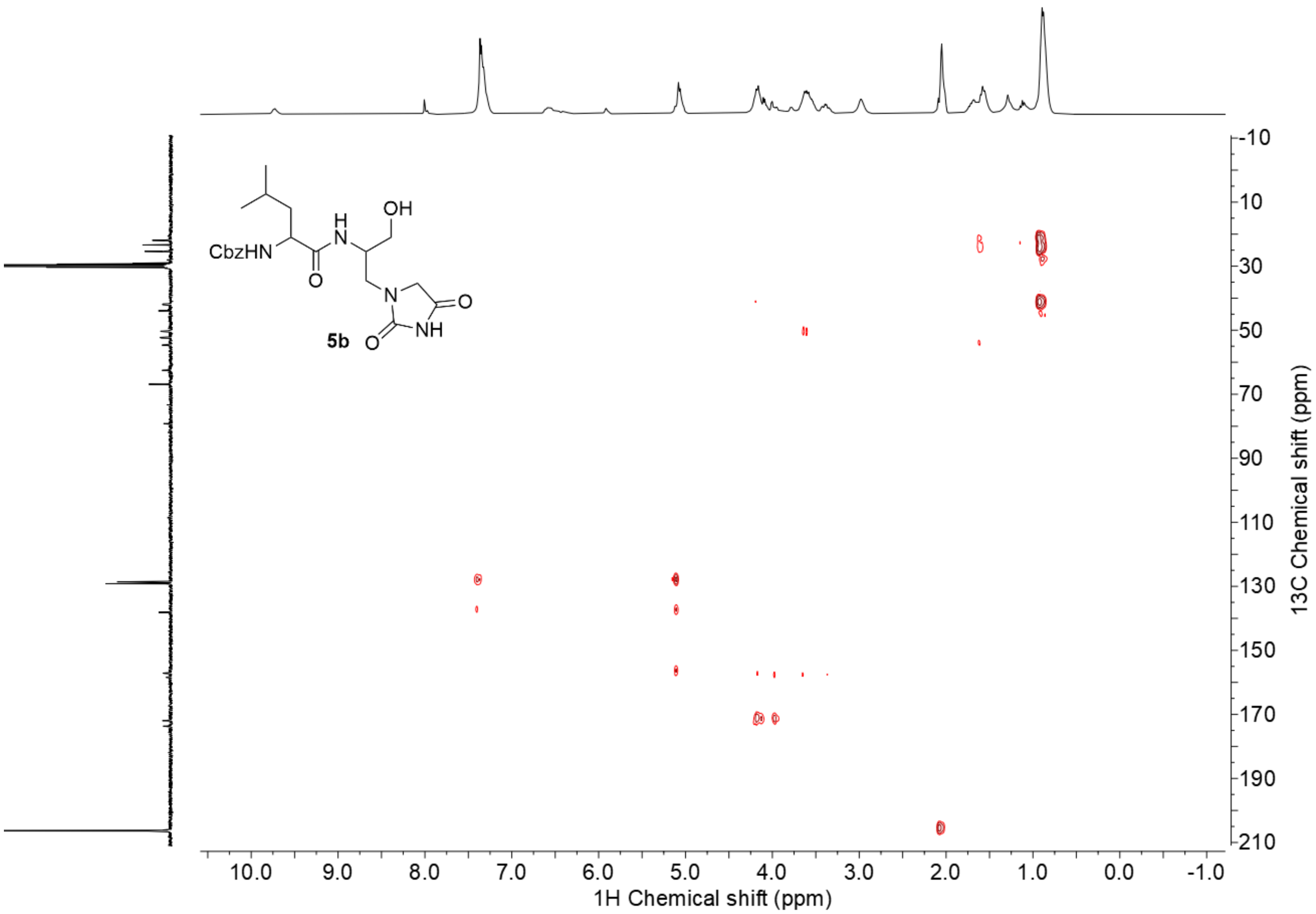

Figure SI36. ^{1}H-^{13}C HMBC NMR (^{1}H 300 MHz, acetone-d_6) of compound **5b**.

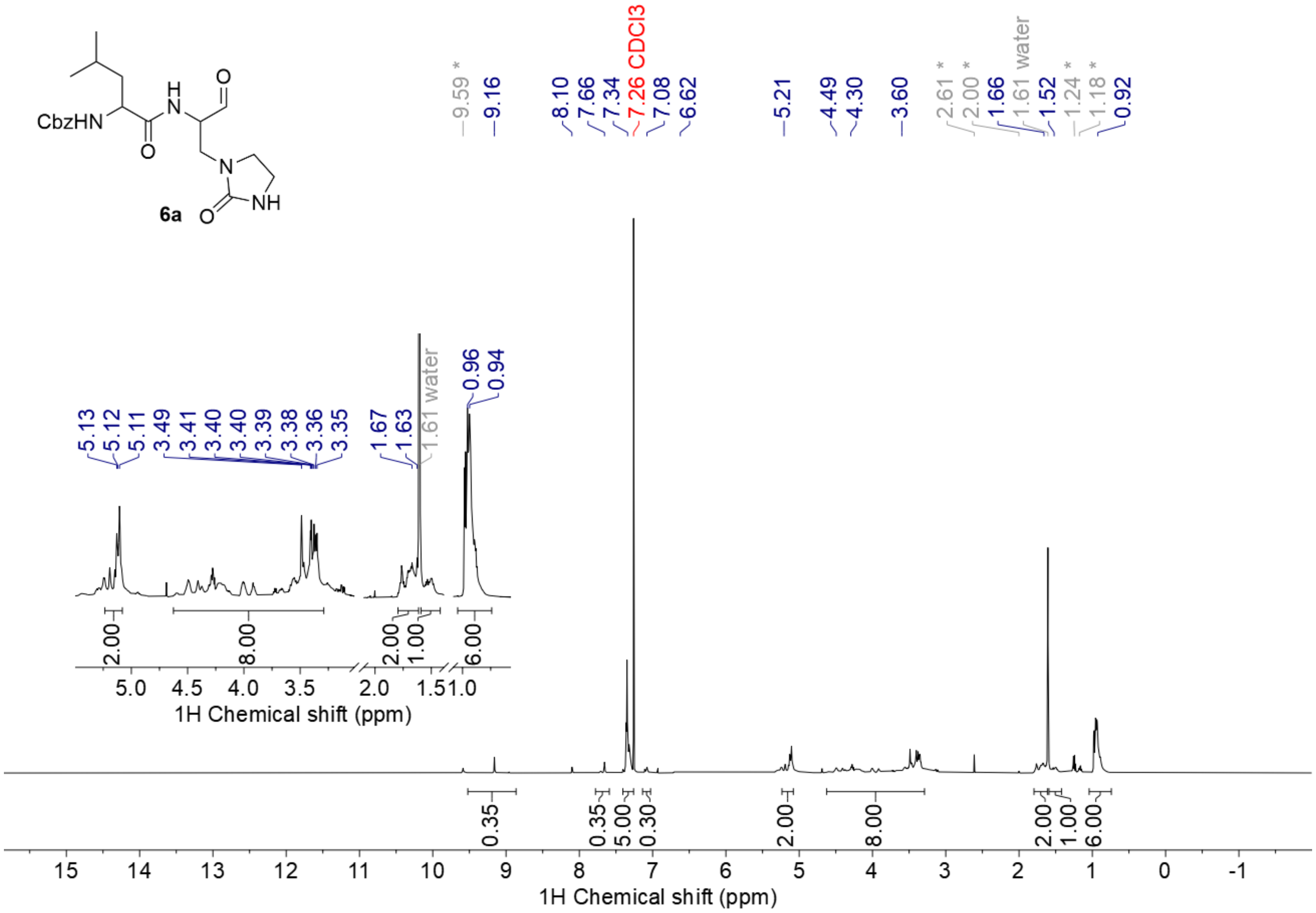

Figure SI37. ^{1}H NMR (700 MHz, $CDCl_3$) of compound **GC373-OxIm 6a**; * – impurities. (See the comment on the stability of the compound at r.t. in the section above.)

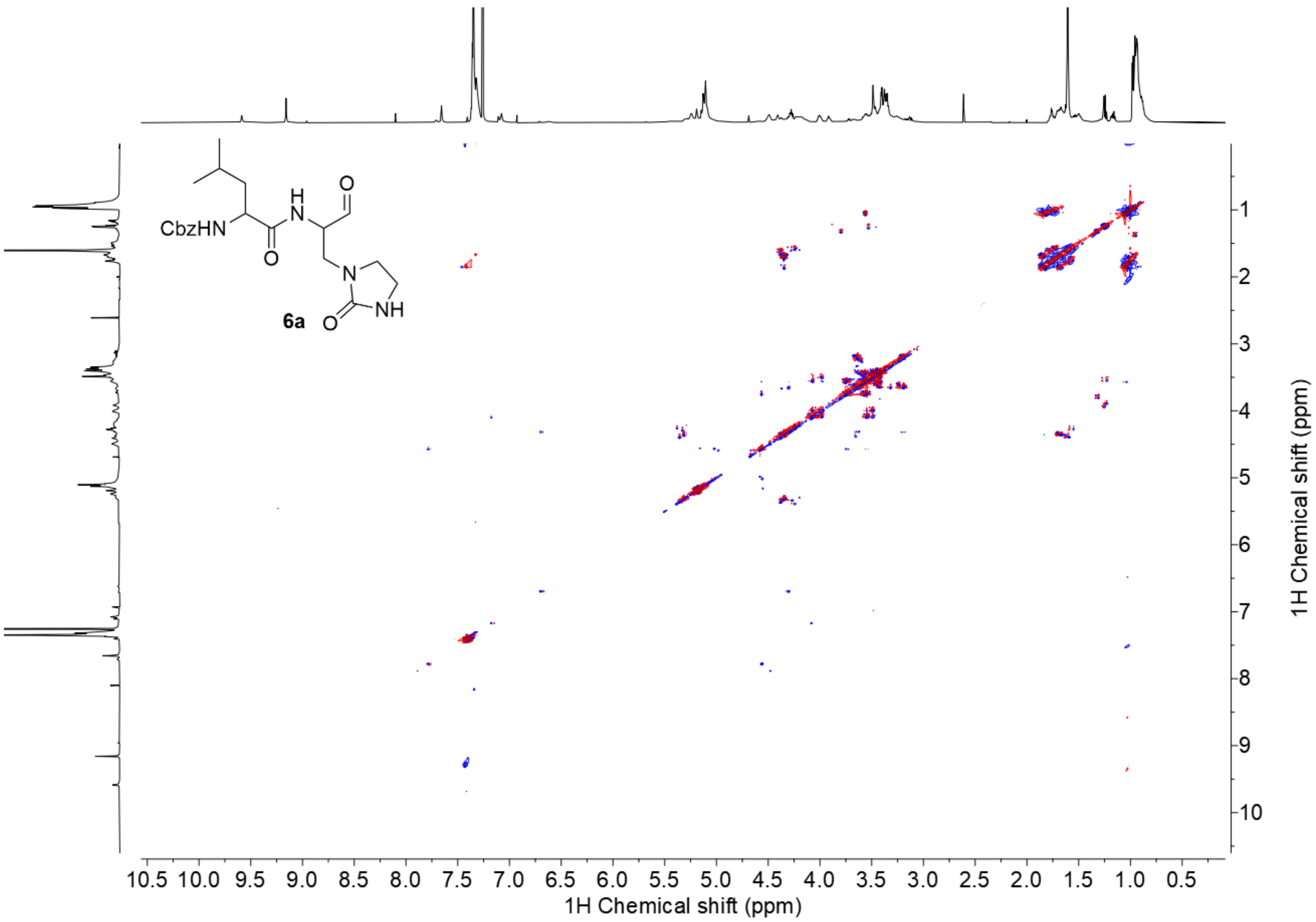

Figure SI38. ^{1}H-^{1}H COSY NMR (^{1}H 700 MHz, $CDCl_3$) of compound **GC373-OxIm 6a**. (See the comment on the stability of the compound at r.t. in the section above.)

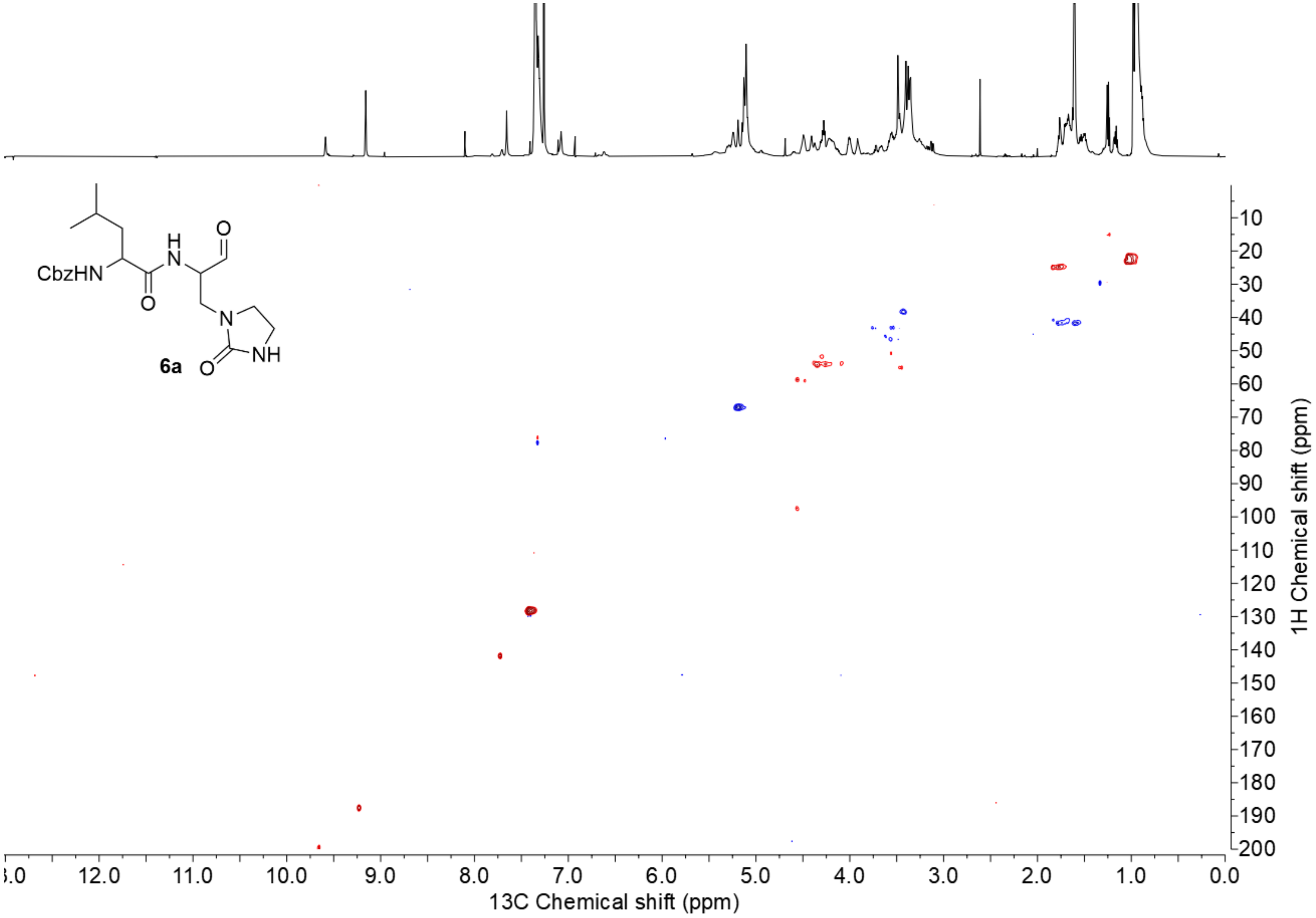

Figure SI39 ^{1}H-^{13}C HSQC-ED NMR (^{1}H 700 MHz, $CDCl_3$) of compound **GC373-OxIm 6a**. (See the comment on the stability of the compound at r.t. in the section above.)

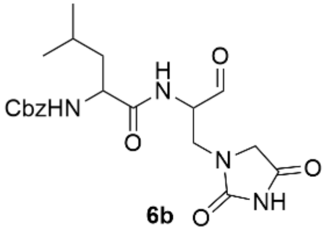

6b

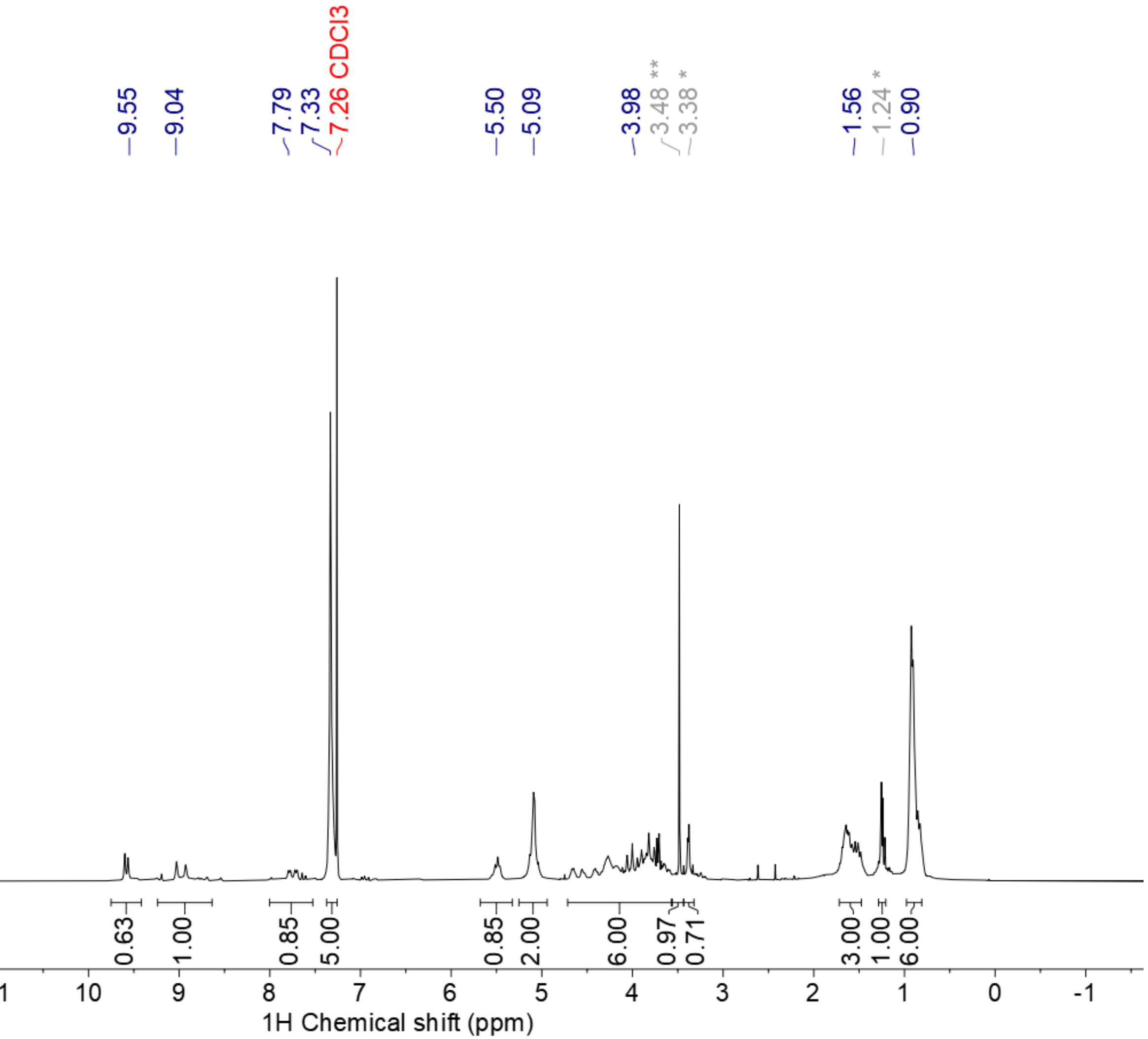

Figure SI40. ^{1}H NMR (300 MHz, $CDCl_3$) of compound **GC373-Hyd 6b**; * – impurities, ** – methanol. (See the comment on the stability of the compound at r.t. in the section above.)

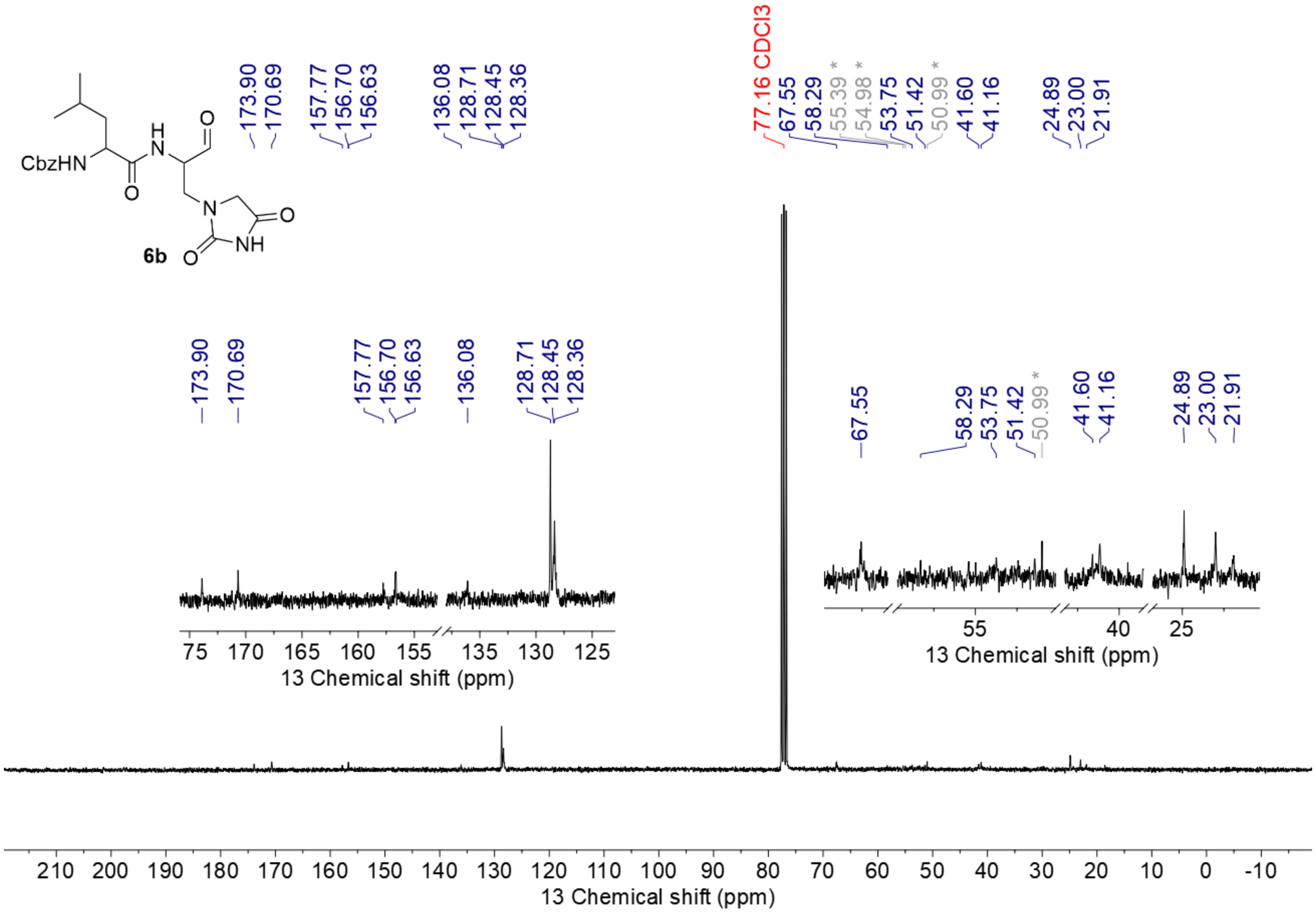

Figure SI41. ^{13}C NMR (75 MHz, $CDCl_3$) of compound **GC373-Hyd 6b**; * – impurities. (See the comment on the stability of the compound at r.t. in the section above.)

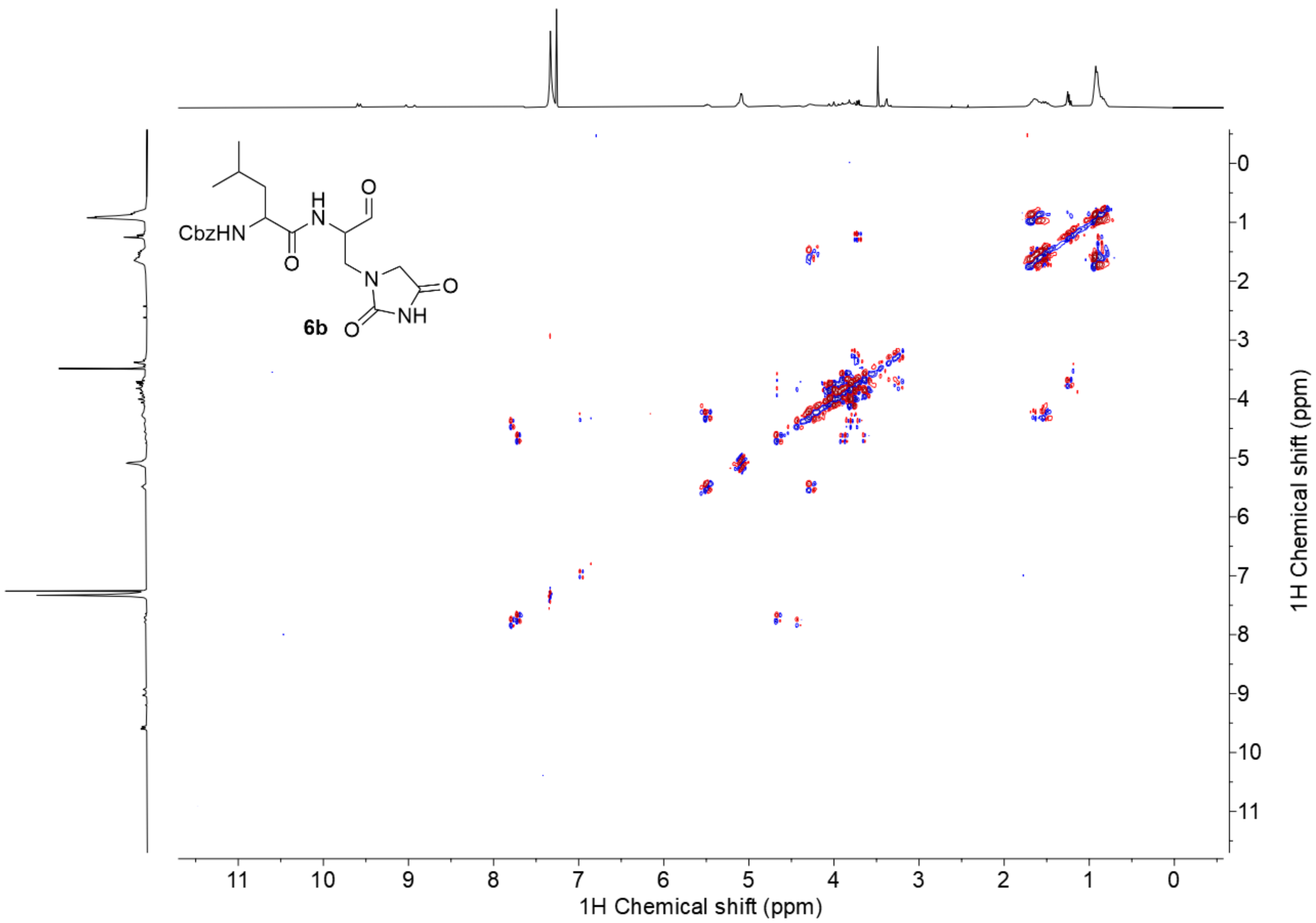

Figure SI42. ^{1}H-^{1}H COSY NMR (^{1}H 300 MHz, $CDCl_3$) of compound **GC373-Hyd 6b**. (See the comment on the stability of the compound at r.t. in the section above.)

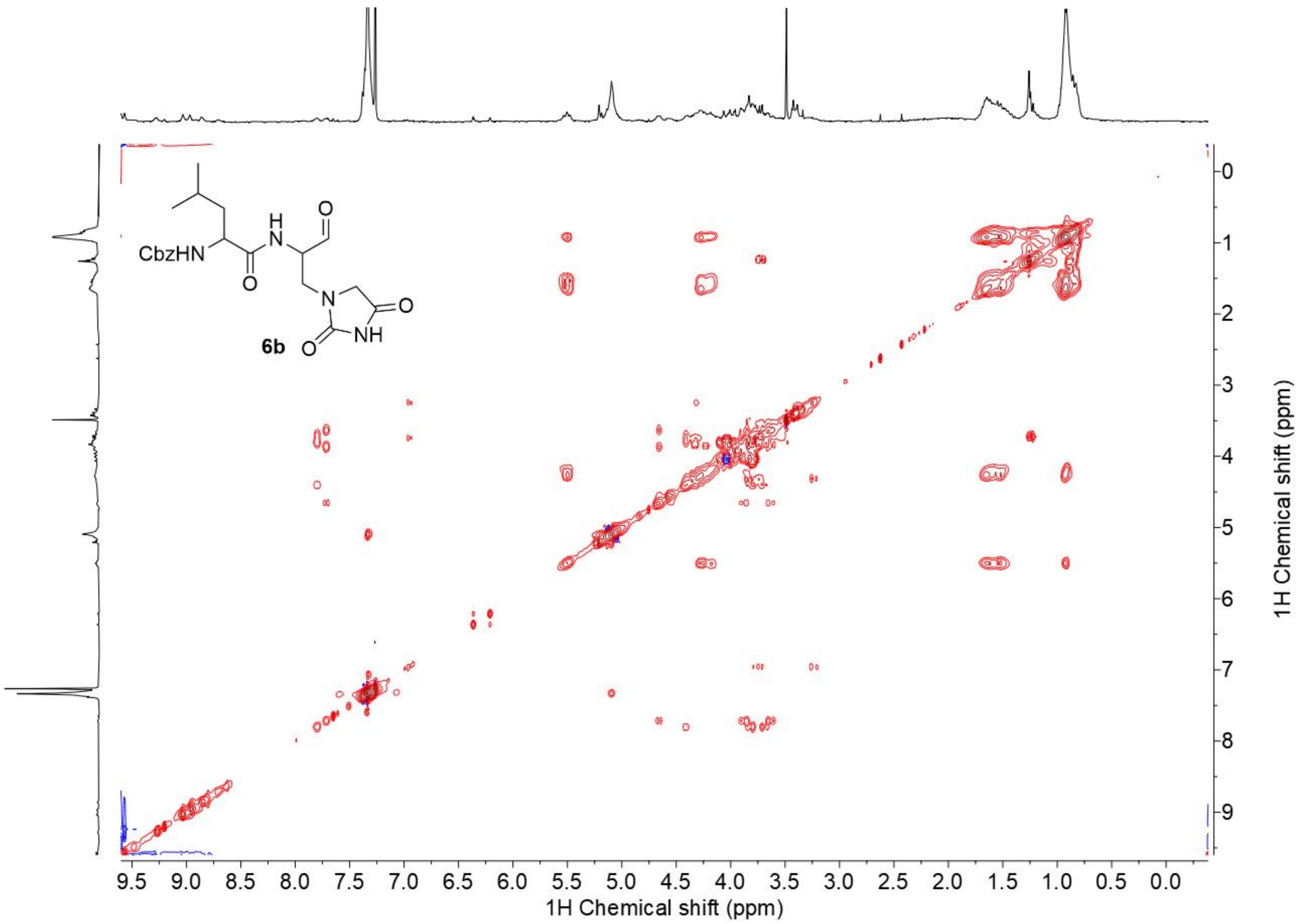

Figure SI43. ^{1}H-^{1}H TOCSY NMR (^{1}H 300 MHz, $CDCl_3$) of compound **GC373-Hyd 6b**. (See the comment on the stability of the compound at r.t. in the section above.)

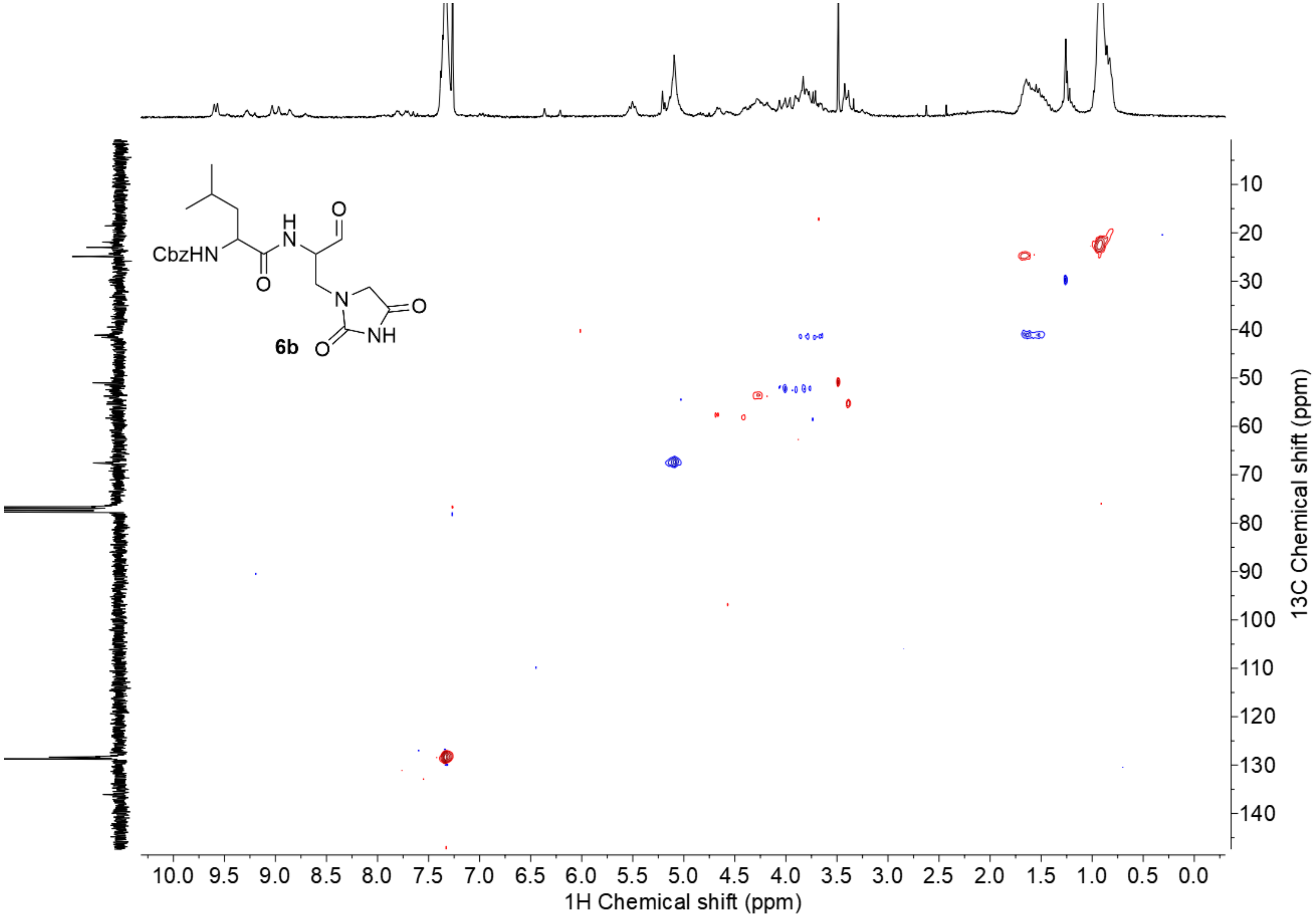

Figure SI44. ^{1}H-^{13}C HSQC-ED NMR (^{1}H 300 MHz, $CDCl_3$) of compound **GC373-Hyd 6b**. (See the comment on the stability of the compound at r.t. in the section above.)

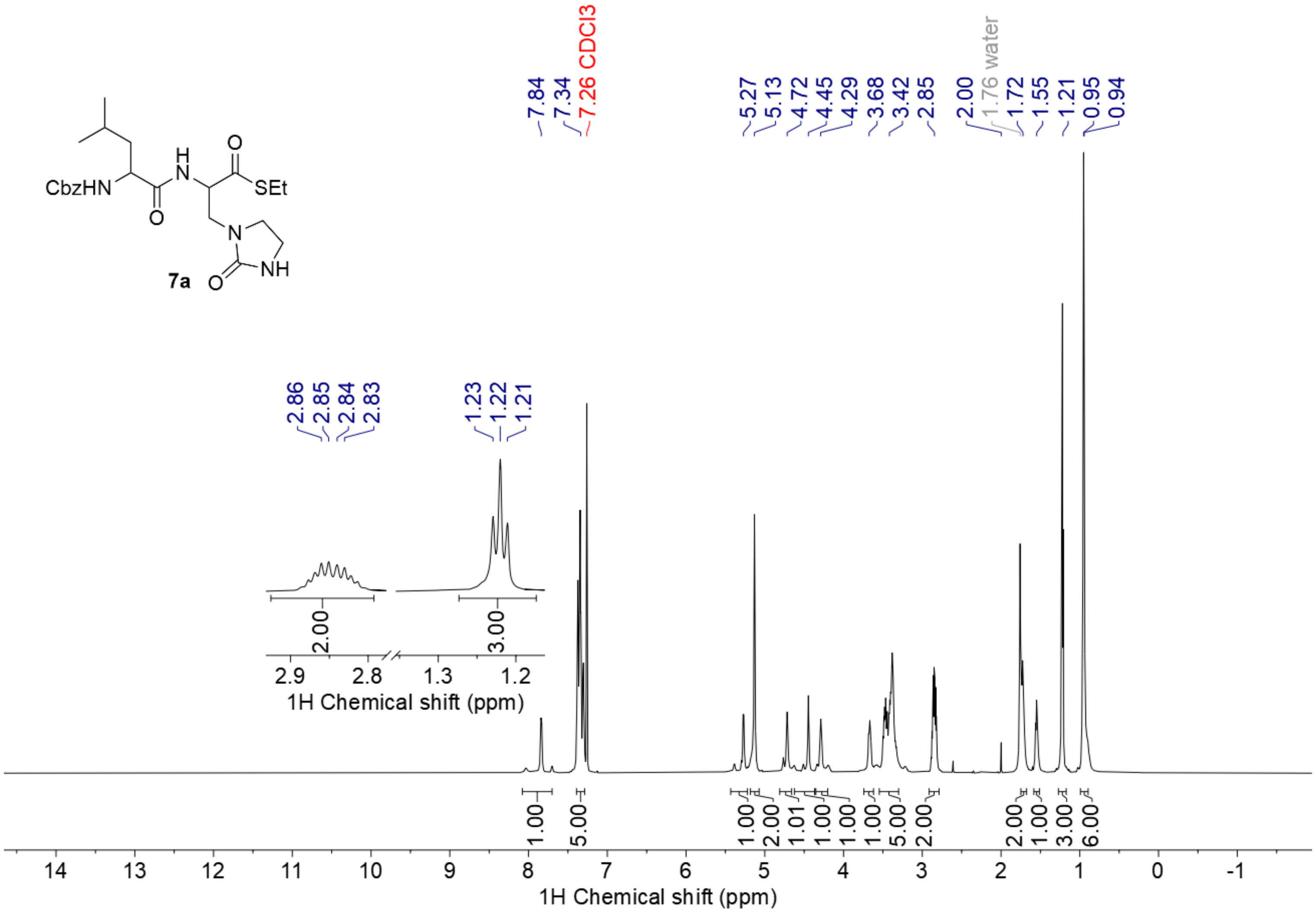

Figure SI45. ^{1}H NMR (800 MHz, $CDCl_3$) of compound **7a**.

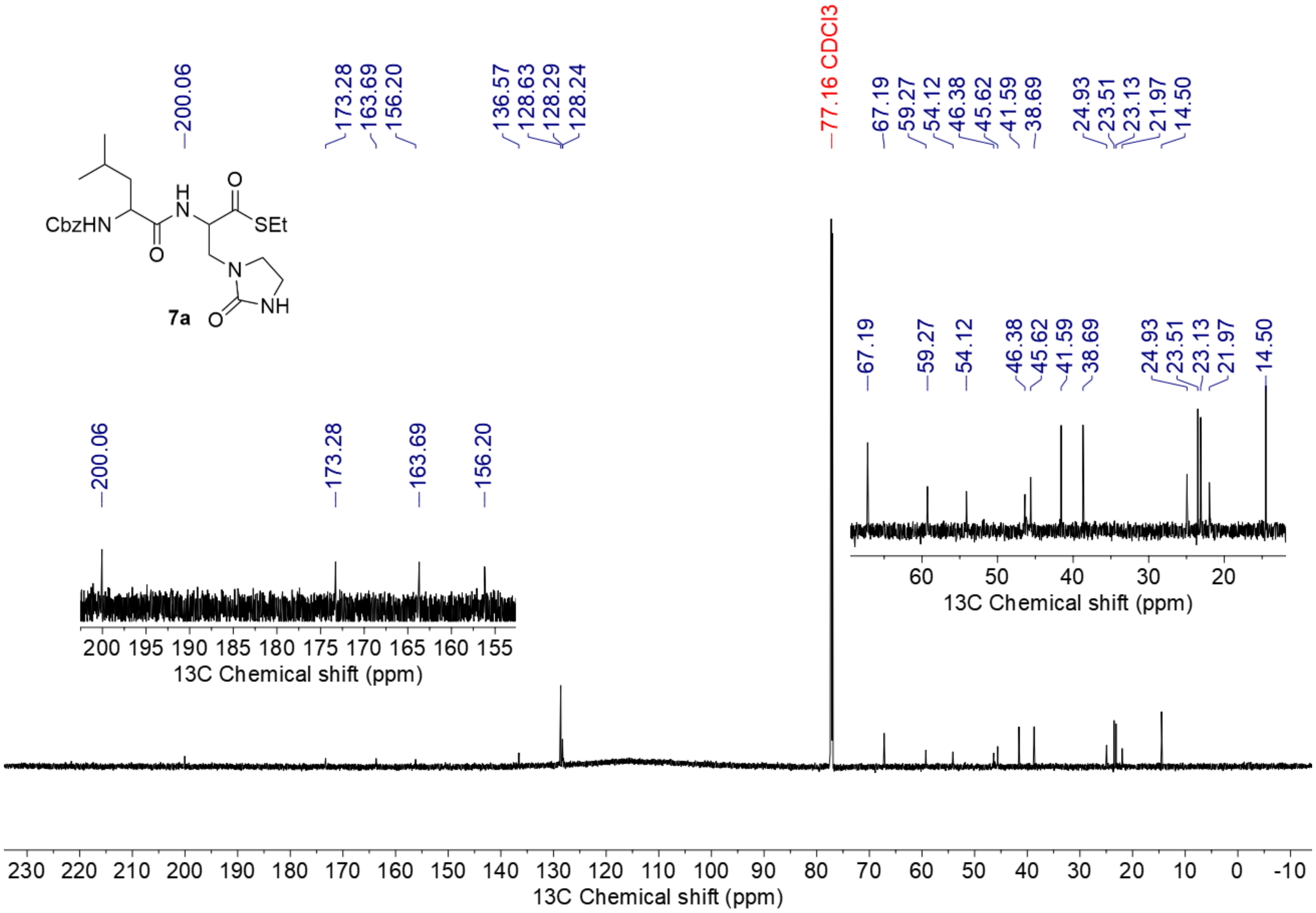

Figure SI46. ^{13}C NMR (201 MHz, $CDCl_3$) of compound **7a**.

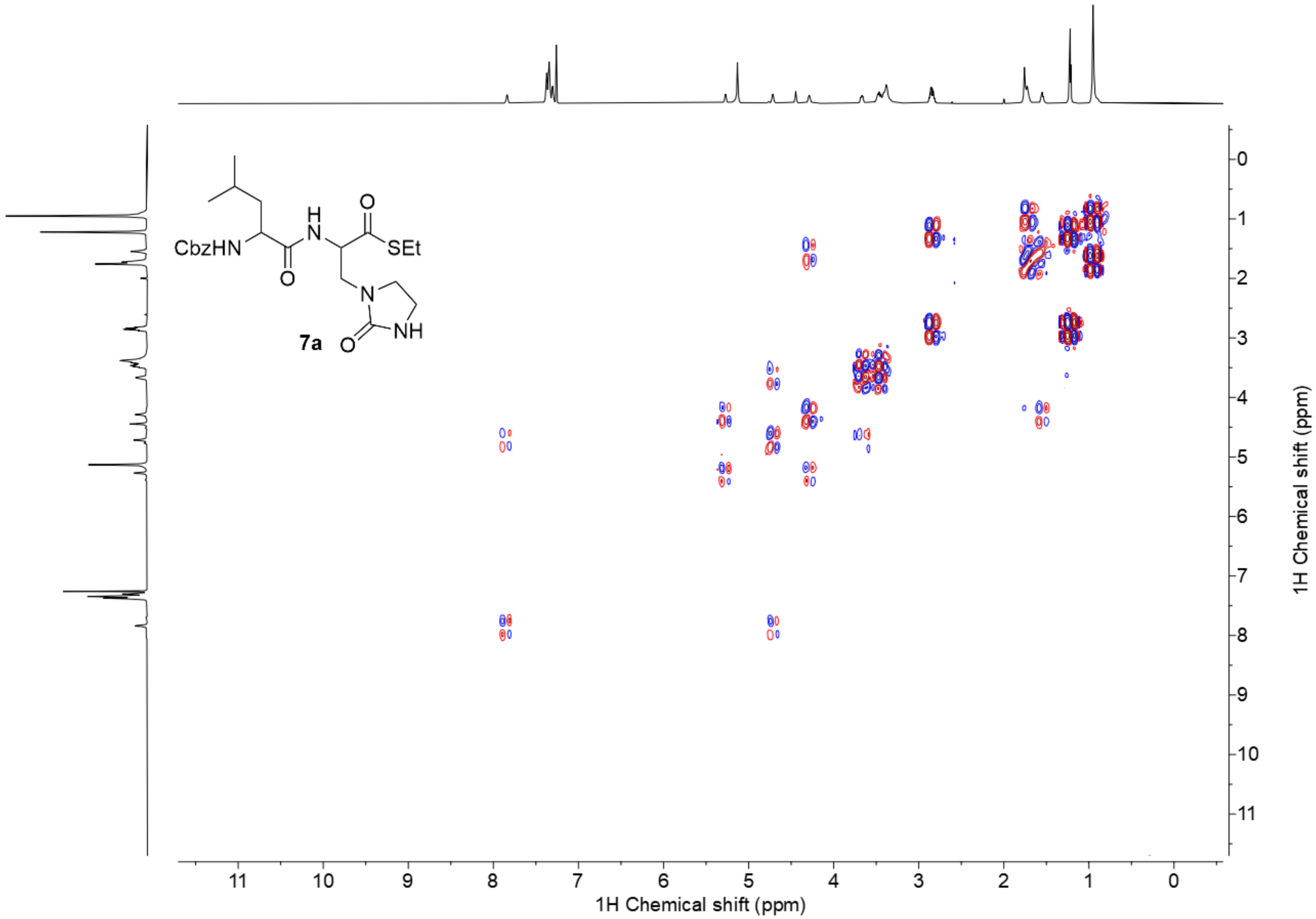

Figure SI47. ^{1}H-^{1}H COSY NMR (^{1}H 800 MHz, $CDCl_3$) of compound **7a**.

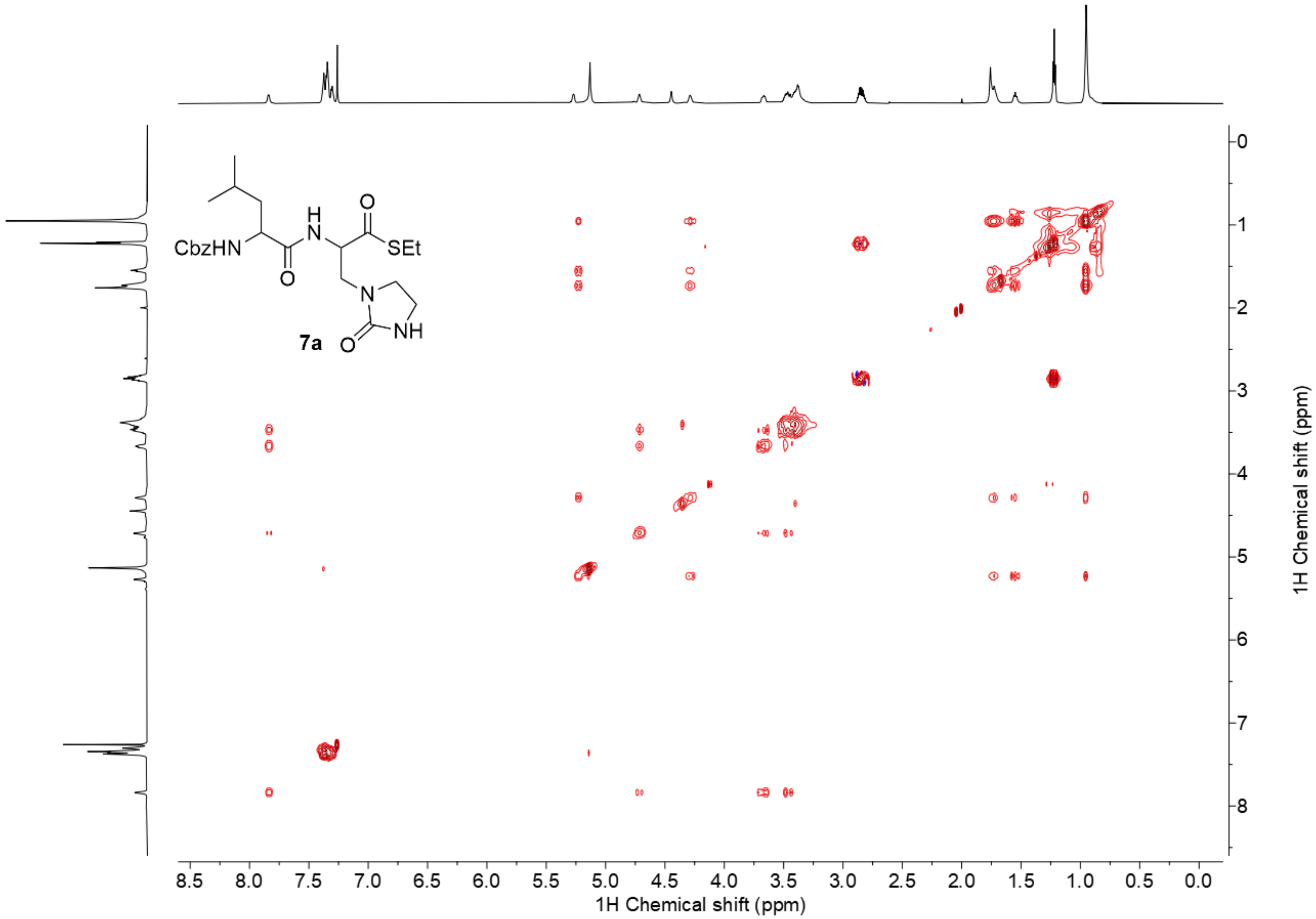

Figure SI48. ^{1}H-^{1}H TOCSY NMR (^{1}H 300 MHz, $CDCl_3$) of compound **7a**.

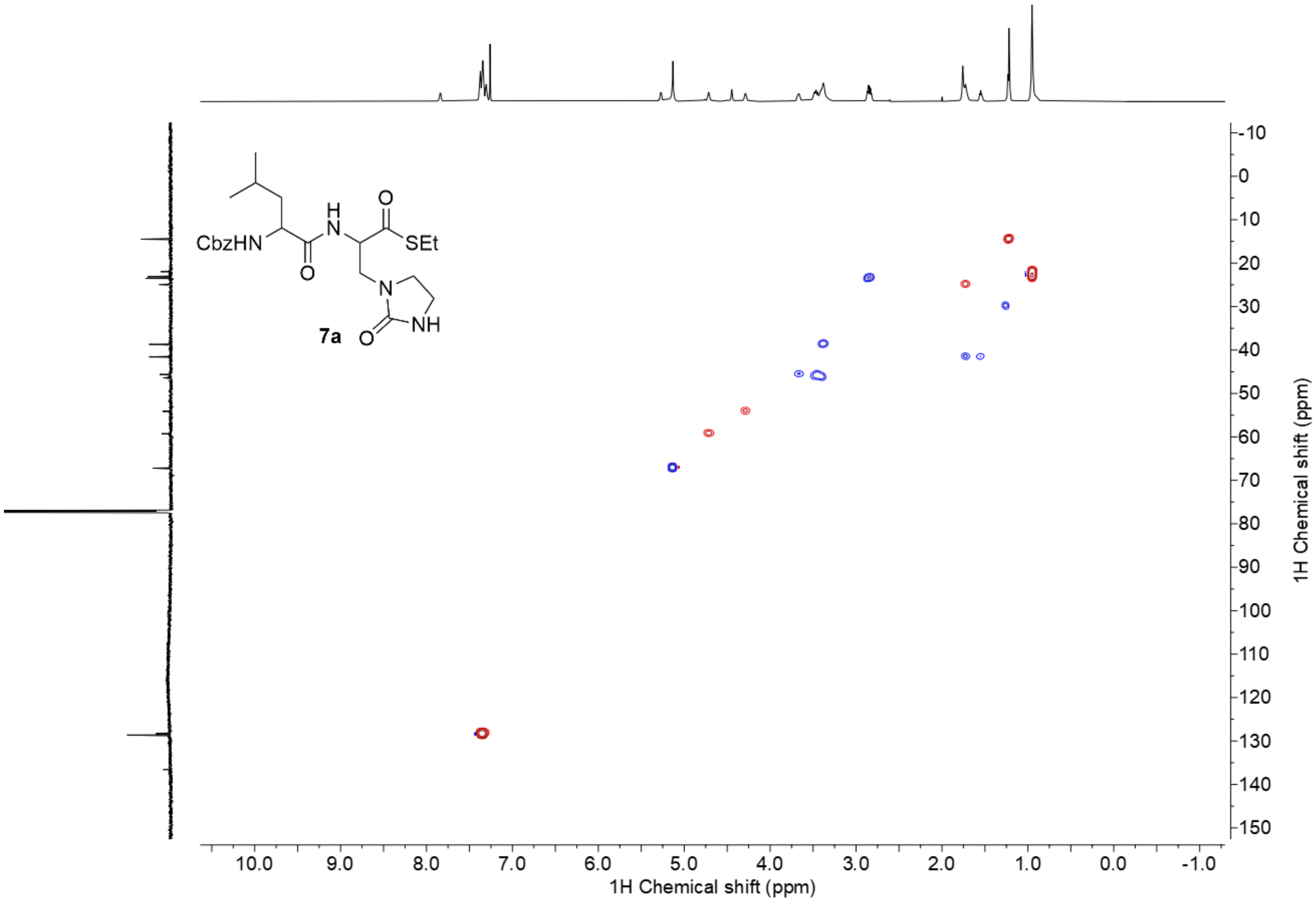

Figure SI49. ^{1}H-^{13}C HSQC-ED NMR (^{1}H 800 MHz, $CDCl_3$) of compound **7a**.

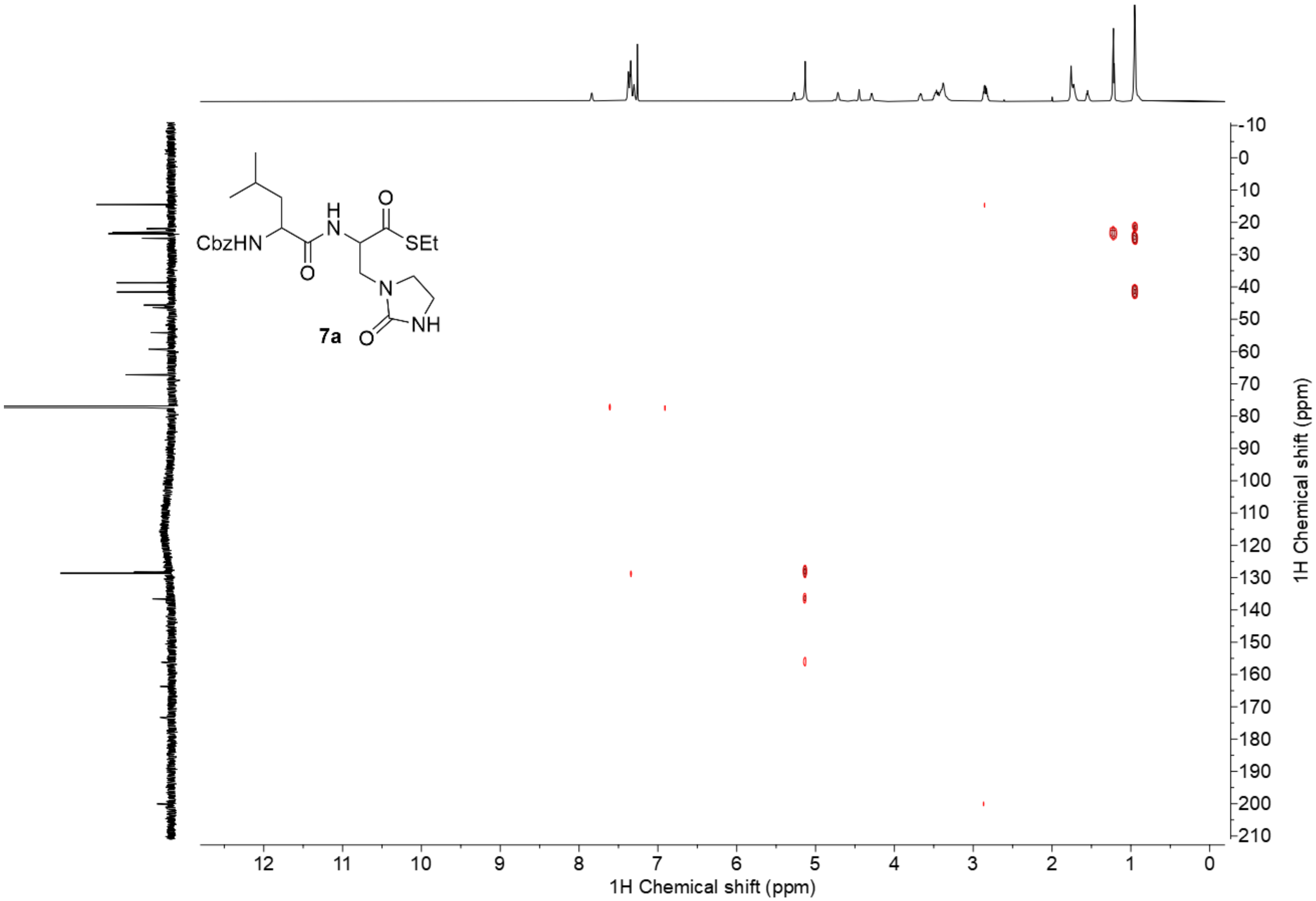

Figure SI50. ^{1}H-^{13}C HMBC NMR (^{1}H 800 MHz, $CDCl_3$) of compound **7a.**

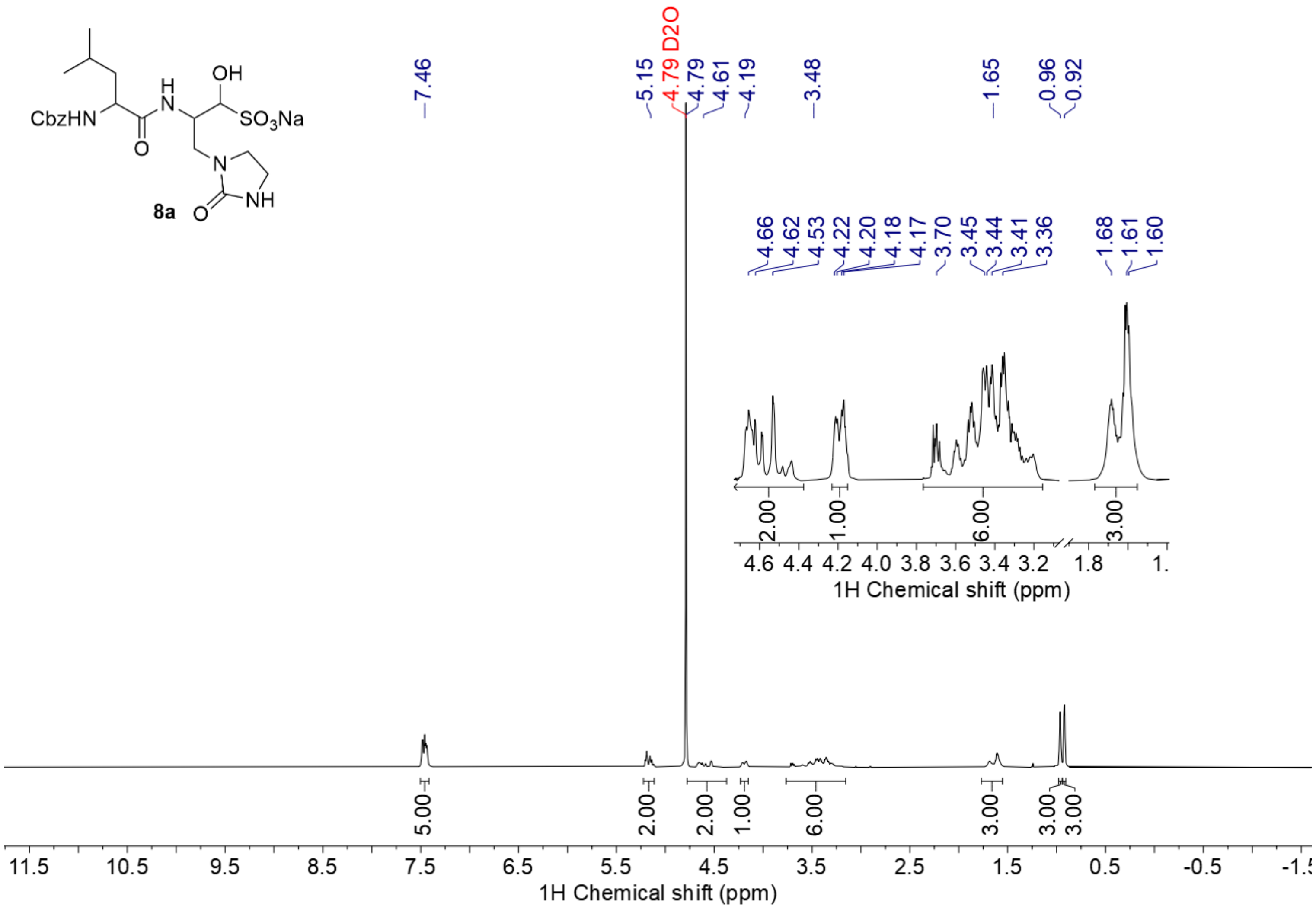

Figure SI51. ^{1}H NMR (800 MHz, D_2O) of compound **8a**.

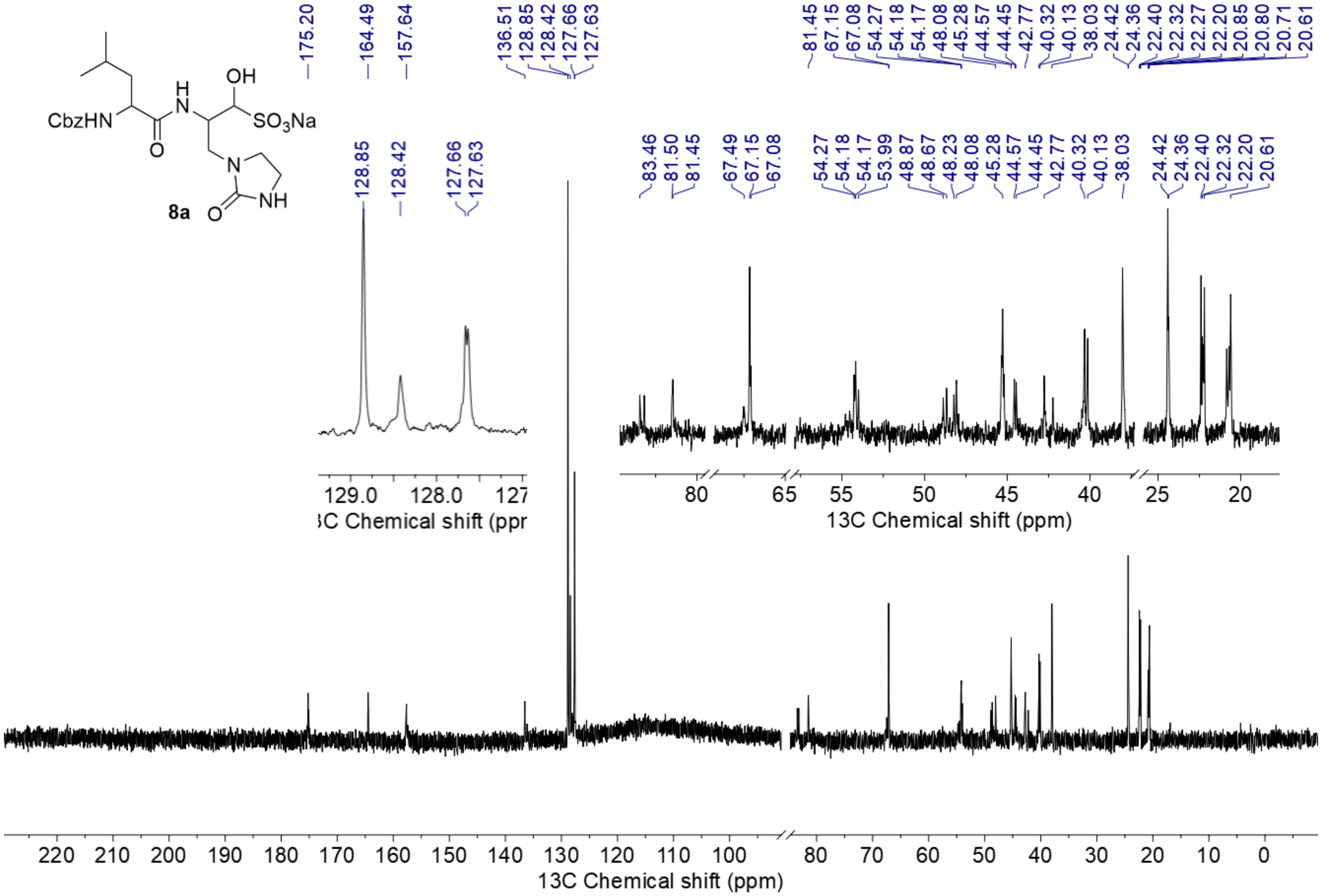

Figure SI52. ^{13}C NMR (201 MHz, D_2O) of compound **8a**.

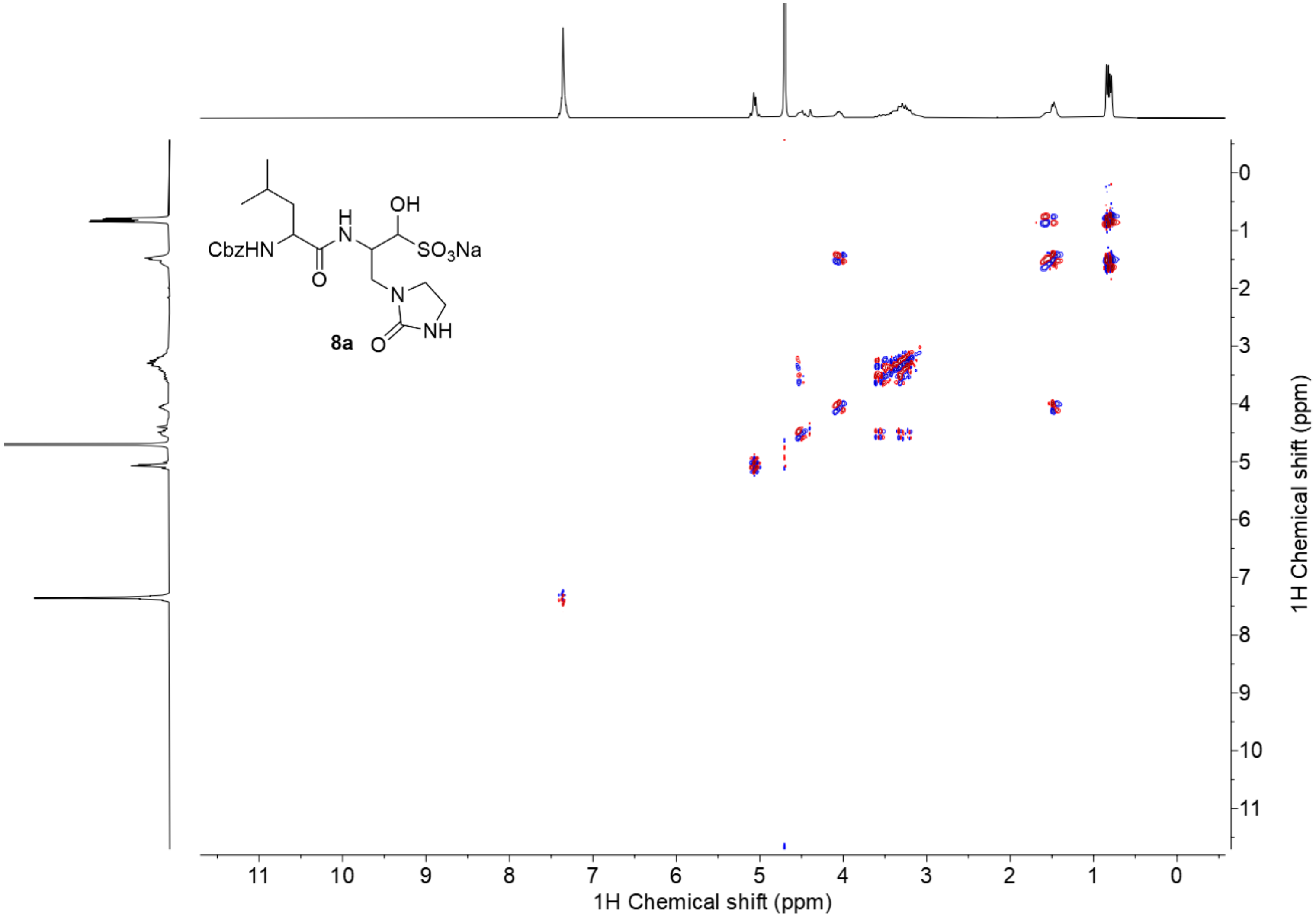

Figure SI53. ^{1}H-^{1}H COSY NMR (^{1}H 300 MHz, D_2O) of compound **8a**.

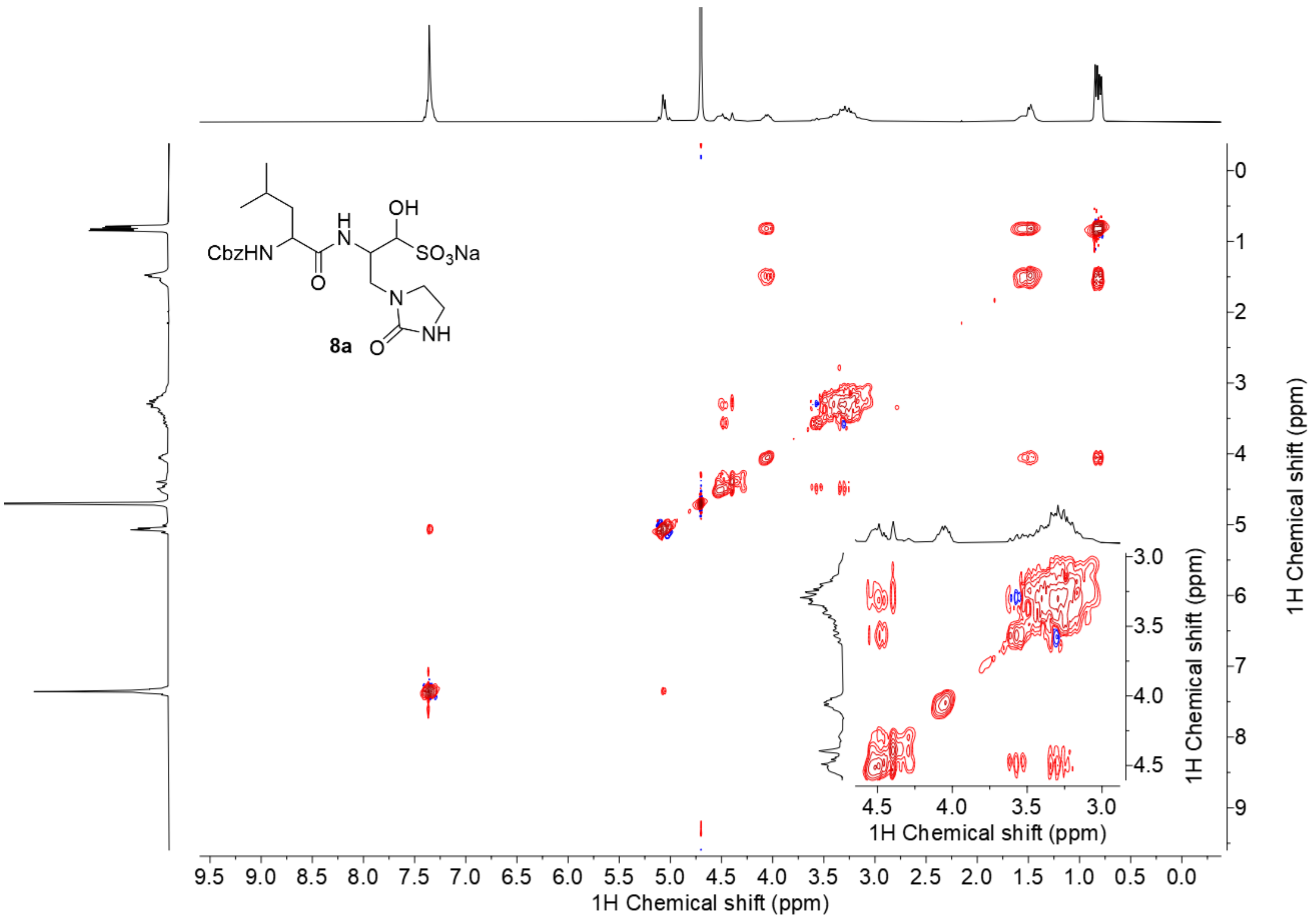

Figure SI54. ^{1}H-^{1}H TOCSY NMR (^{1}H 300 MHz, D_2O) of compound **8a**.

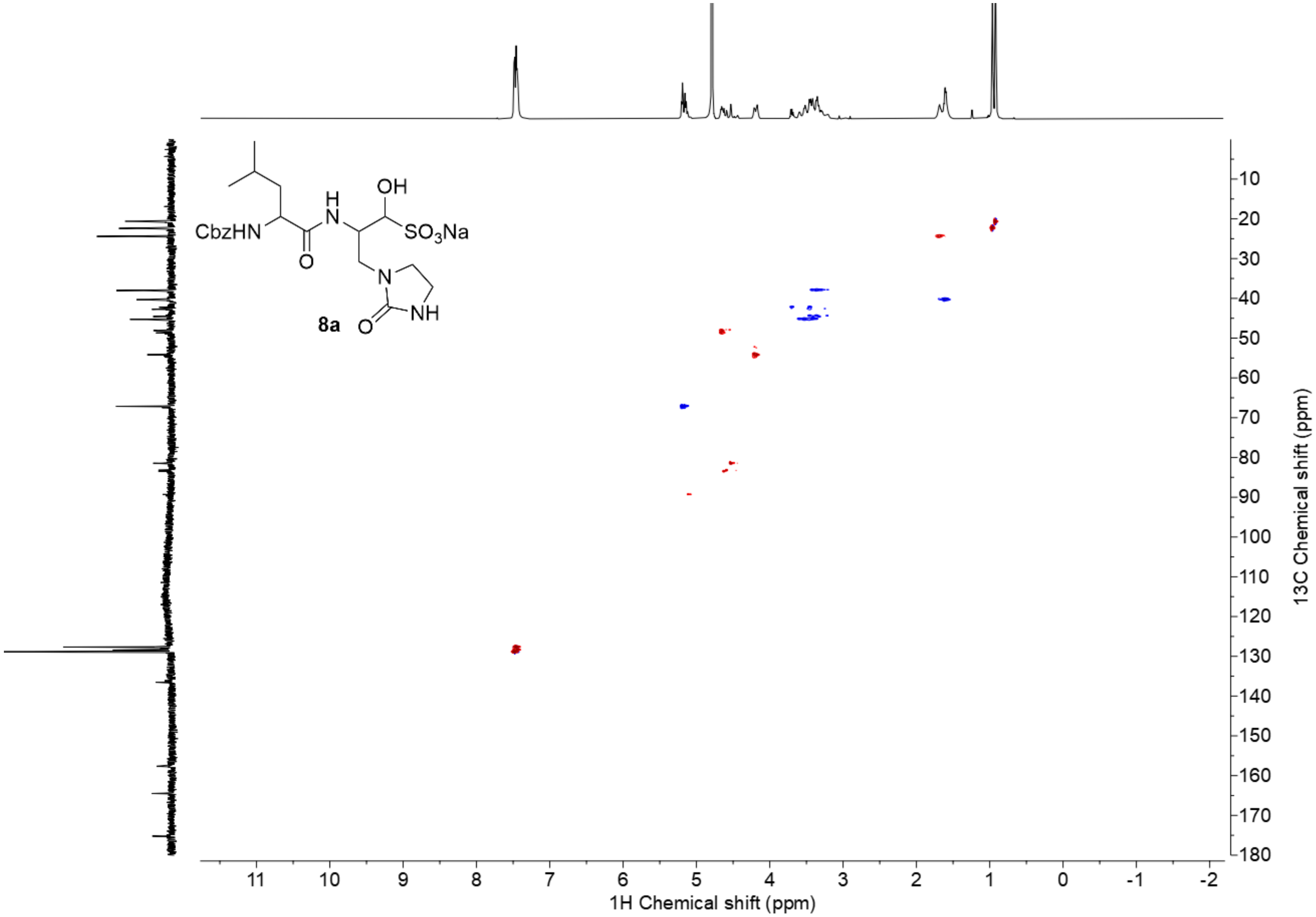

Figure SI55. ^{1}H-^{13}C HSQC-ED NMR (^{1}H 800 MHz, D_2O) of compound **8a**.

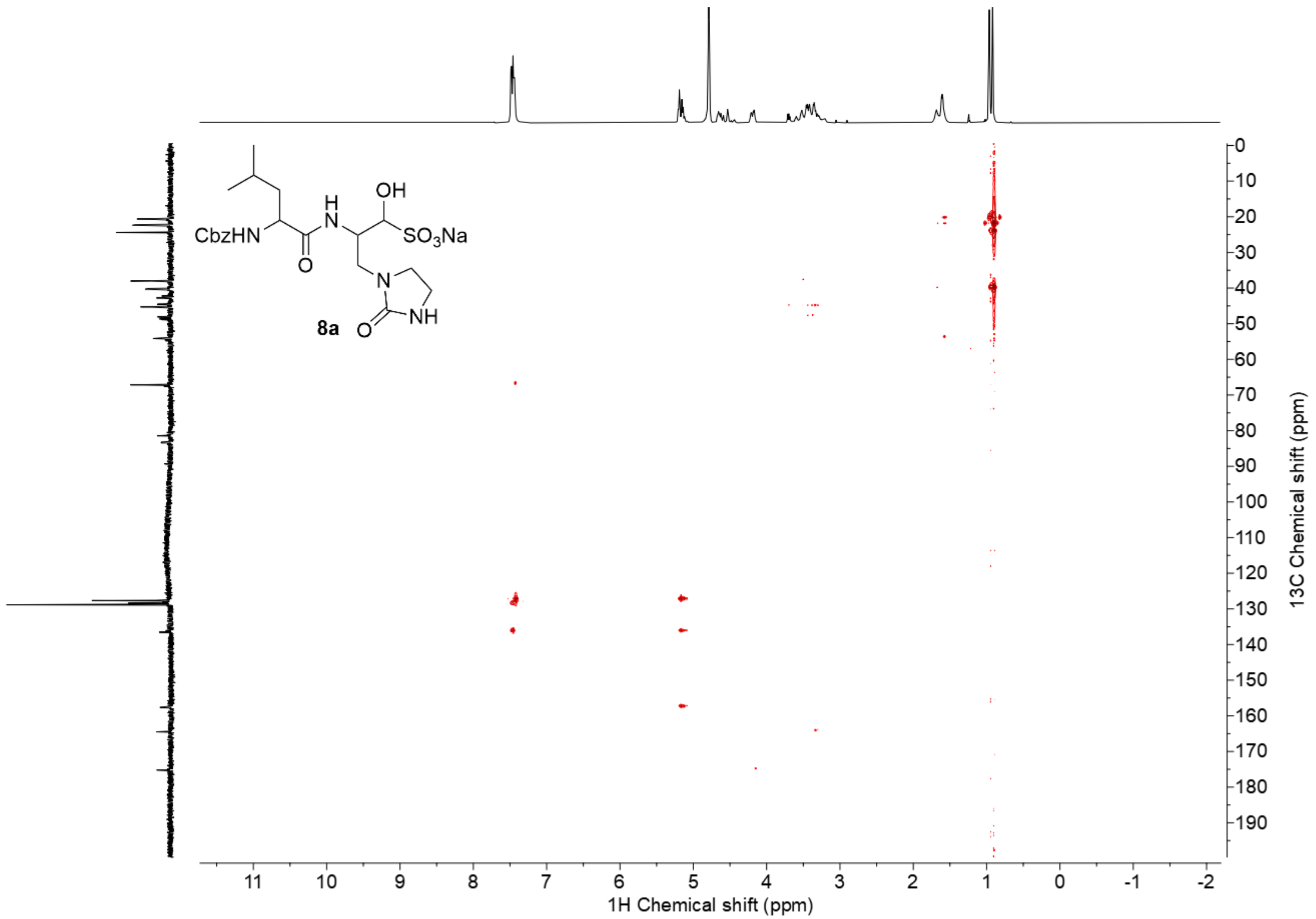

Figure SI56. ^{1}H-^{13}C HMBC NMR (^{1}H 800 MHz, D_2O) of compound **8a.**

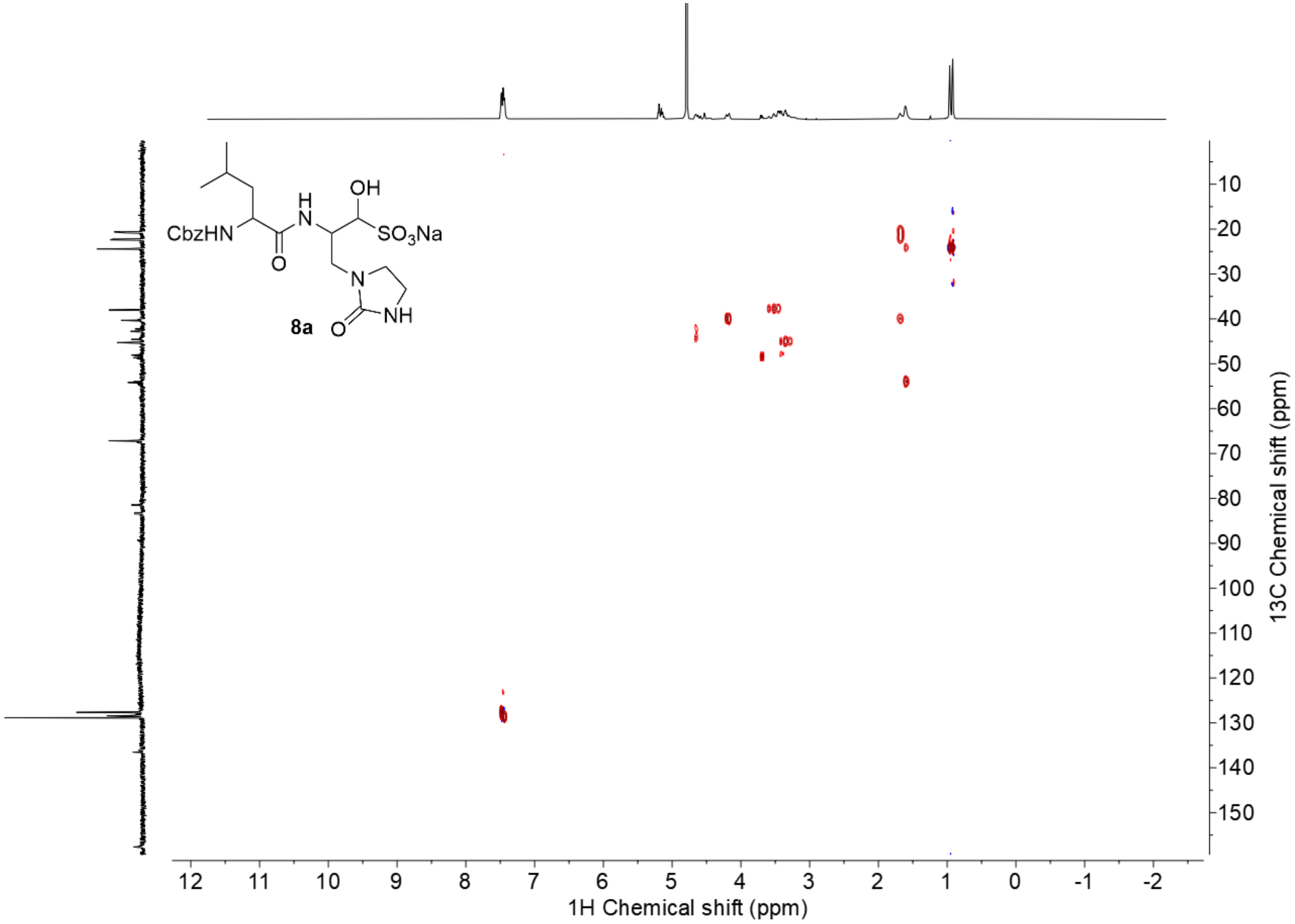

Figure SI57. ^{1}H-^{13}C H2BC NMR (^{1}H 800 MHz, D_2O) of compound **8a.**

Kinetic Experiments (Figures)

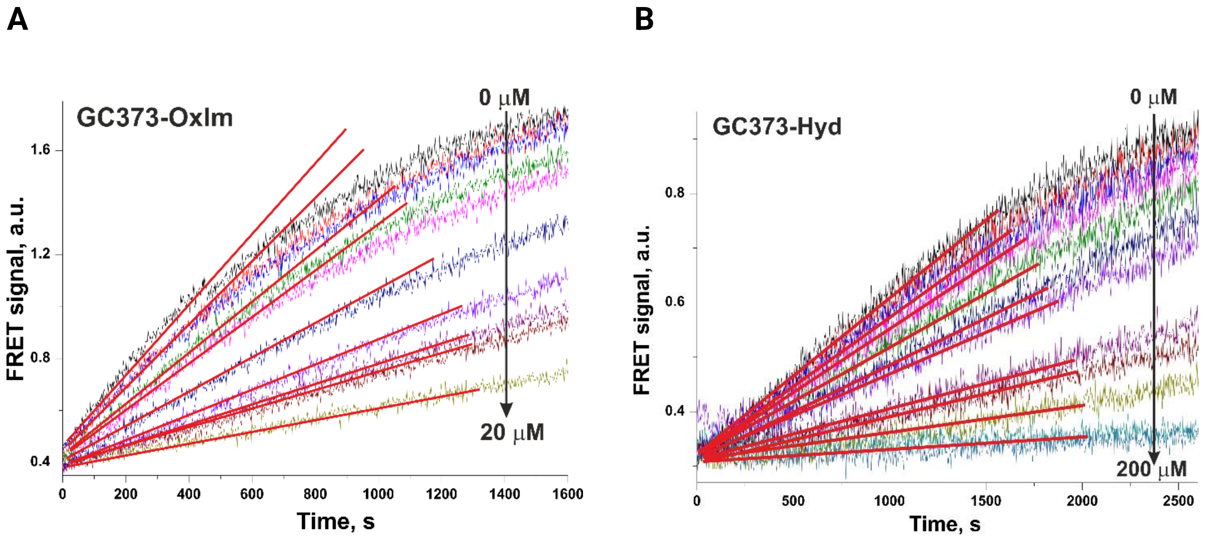

Figure SI58. Kinetic fluorescence curves characterizing the process of FRET-S hydrolysis (30 μM) in the presence of M^{pro} (150 nM) and different concentrations of **GC373-OxIm** (from 0 to 20 μM) (A) and **GC373-Hyd** (from 0 to 200 μM) (B).

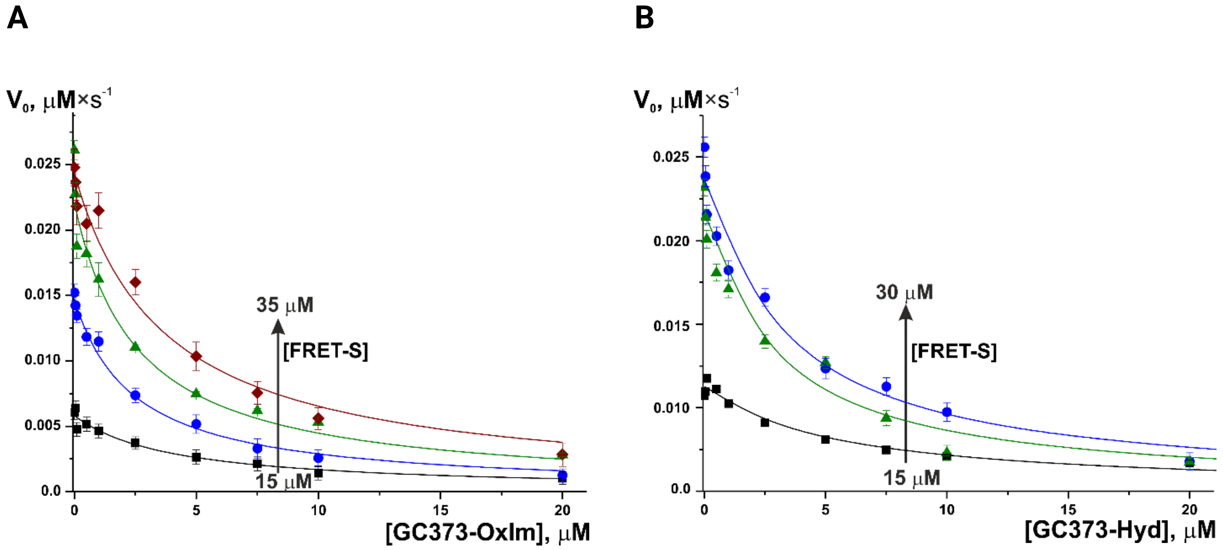

Figure SI59. Dependence of the initial rate of substrate hydrolysis on the inhibitor concentration at a constant enzyme concentration (150 nM) for **GC373-OxIm** (15, 20, 30, 35 μM) (A) and **GC373-Hyd** (15, 20, 30 μM) (B).

References

1. Daina, A.; Michielin, O.; Zoete, V. SwissADME: A Free Web Tool to Evaluate Pharmacokinetics, Drug-Likeness and Medicinal Chemistry Friendliness of Small Molecules. *Sci. Rep.* **2017**, *7*, 42717, doi:10.1038/srep42717.
2. Ali, J.; Camilleri, P.; Brown, M.B.; Hutt, A.J.; Kirton, S.B. Revisiting the General Solubility Equation: In Silico Prediction of Aqueous Solubility Incorporating the Effect of Topographical Polar Surface Area. *J. Chem. Inf. Model.* **2012**, *52*, 420–428, doi:10.1021/ci200387c.
3. Delaney, J.S. ESOL: Estimating Aqueous Solubility Directly from Molecular Structure. *J. Chem. Inf. Comput. Sci.* **2004**, *44*, 1000–1005, doi:10.1021/ci034243x.
4. Daina, A.; Zoete, V. A BOILED-Egg To Predict Gastrointestinal Absorption and Brain Penetration of Small Molecules. *ChemMedChem* **2016**, *11*, 1117–1121, doi:10.1002/cmdc.201600182.
5. Ertl, P.; Rohde, B.; Selzer, P. Fast Calculation of Molecular Polar Surface Area as a Sum of Fragment-Based Contributions and Its Application to the Prediction of Drug Transport Properties. *J. Med. Chem.* **2000**, *43*, 3714–3717, doi:10.1021/jm000942e.
6. Lipinski, C.A.; Lombardo, F.; Dominy, B.W.; Feeney, P.J. Experimental and Computational Approaches to Estimate Solubility and Permeability in Drug Discovery and Development Settings1. *Adv. Drug Deliv. Rev.* **2001**, *46*, 3–26, doi:10.1016/S0169-409X(00)00129-0.
7. Ghose, A.K.; Viswanadhan, V.N.; Wendoloski, J.J. A Knowledge-Based Approach in Designing Combinatorial or Medicinal Chemistry Libraries for Drug Discovery. 1. A Qualitative and Quantitative Characterization of Known Drug Databases. *J. Comb. Chem.* **1999**, *1*, 55–68, doi:10.1021/cc9800071.
8. Veber, D.F.; Johnson, S.R.; Cheng, H.-Y.; Smith, B.R.; Ward, K.W.; Kopple, K.D. Molecular Properties That Influence the Oral Bioavailability of Drug Candidates. *J. Med. Chem.* **2002**, *45*, 2615–2623, doi:10.1021/jm020017n.
9. Egan, W.J.; Merz, Kenneth M.; Baldwin, J.J. Prediction of Drug Absorption Using Multivariate Statistics. *J. Med. Chem.* **2000**, *43*, 3867–3877, doi:10.1021/jm000292e.
10. Muegge, I.; Heald, S.L.; Brittelli, D. Simple Selection Criteria for Drug-like Chemical Matter. *J. Med. Chem.* **2001**, *44*, 1841–1846, doi:10.1021/jm015507e.
11. Banerjee, P.; Kemmler, E.; Dunkel, M.; Preissner, R. ProTox 3.0: A Webserver for the Prediction of Toxicity of Chemicals. *Nucleic Acids Res.* **2024**, *52*, W513–W520, doi:10.1093/nar/gkae303.
12. Makhin, A.P.; Miturich, V.S.; Vavilov, M.V.; Lyakhovich, M.S.; Andrianova, A.A.; Zagitova, R.I.; Shmygarev, V.I.; Fadeeva, A.A.; Yatskin, O.N.; Belozerova, O.A.; et al. Improved Synthesis of Two Quisqualic Acid Analogs Containing Hydantoin and Imidazolidinone Moieties. *Chem. Heterocycl. Compd.* **2024**, *60*, 262–268, doi:10.1007/s10593-024-03331-1.
13. Mancuso, A.J.; Huang, S.-L.; Swern, D. Oxidation of Long-Chain and Related Alcohols to Carbonyls by Dimethyl Sulfoxide "Activated" by Oxalyl Chloride. *J. Org. Chem.* **1978**, *43*, 2480–2482, doi:10.1021/jo00406a041.
14. Parikh, J.R.; Doering, W. v. E. Sulfur Trioxide in the Oxidation of Alcohols by Dimethyl Sulfoxide. *J. Am. Chem. Soc.* **1967**, *89*, 5505–5507, doi:10.1021/ja00997a067.
15. Tidwell, T.T. Oxidation of Alcohols by Activated Dimethyl Sulfoxide and Related Reactions: An Update. *Synthesis* **2002**, *1990*, 857–870, doi:10.1055/s-1990-

27036.
16. Dess, D.B.; Martin, J.C. Readily Accessible 12-I-5 Oxidant for the Conversion of Primary and Secondary Alcohols to Aldehydes and Ketones. *J. Org. Chem.* **1983**, *48*, 4155–4156, doi:10.1021/jo00170a070.
17. Dess, D.B.; Martin, J.C. A Useful 12-I-5 Triacetoxyperiodinane (the Dess-Martin Periodinane) for the Selective Oxidation of Primary or Secondary Alcohols and a Variety of Related 12-I-5 Species. *J. Am. Chem. Soc.* **1991**, *113*, 7277–7287, doi:10.1021/ja00019a027.
18. Meyer, S.D.; Schreiber, S.L. Acceleration of the Dess-Martin Oxidation by Water. *J. Org. Chem.* **1994**, *59*, 7549–7552, doi:10.1021/jo00103a067.
19. Fukuyama, T.; Lin, S.C.; Li, L. Facile Reduction of Ethyl Thiol Esters to Aldehydes: Application to a Total Synthesis of (+)-Neothramycin A Methyl Ether. *J. Am. Chem. Soc.* **1990**, *112*, 7050–7051, doi:10.1021/ja00175a043.
20. Tokuyama, H.; Yokoshima, S.; Yamashita, T.; Shao-Cheng, L.; Leping, L.; Fukuyama, T. Facile Palladium-Mediated Conversion of Ethanethiol Esters to Aldehydes and Ketones. *J. Braz. Chem. Soc.* **1998**, *9*, 381–387, doi:10.1590/S0103-50531998000400011.
21. Tokuyama, H.; Yokoshima, S.; Lin, S.-C.; Li, L.; Fukuyama, T. Reduction of Ethanethiol Esters to Aldehydes. *Synthesis* **2002**, *2002*, 1121–1123, doi:10.1055/s-2002-31969.
22. Vuong, W.; Khan, M.B.; Fischer, C.; Arutyunova, E.; Lamer, T.; Shields, J.; Saffran, H.A.; McKay, R.T.; van Belkum, M.J.; Joyce, M.A.; et al. Feline Coronavirus Drug Inhibits the Main Protease of SARS-CoV-2 and Blocks Virus Replication. *Nat. Commun.* **2020**, *11*, 4282, doi:10.1038/s41467-020-18096-2.
